# A Systematic Review and Meta-Analysis of the Prevalence and Risk Factors of Depression in Type 2 Diabetes Patients in China

**DOI:** 10.3389/fmed.2022.759499

**Published:** 2022-05-10

**Authors:** Xiaobo Liu, Yuxi Li, Li Guan, Xia He, Huiling Zhang, Jun Zhang, Juan Li, Dongling Zhong, Rongjiang Jin

**Affiliations:** ^1^School of Health Preservation and Rehabilitation, Chengdu University of Traditional Chinese Medicine, Chengdu, China; ^2^Department of Rehabilitation, Fushun County People's Hospital, Zigong, China; ^3^Affiliated Rehabilitation Hospital of Chengdu University of Traditional Chinese Medicine /Sichuan Province Rehabilitation Hospital, Chengdu, China

**Keywords:** type 2 diabetes mellitus, depression, prevalence, risk factors, China

## Abstract

**Background:**

The prevalence of type 2 diabetes mellitus (T2DM) is increasing in China. Depression in patients with T2DM interferes with blood glucose management, leads to poor treatment outcomes, and has a high risk of dementia and cardiovascular event. We conducted this systematic review and meta-analysis to evaluate the prevalence of depression in patients with T2DM in China and explore potential risk factors associated with depression in T2DM.

**Methods:**

We conducted a literature search in MEDLINE/PubMed, EMBASE, the Cochrane Library, the Chinese Biomedical Literature Database (CBM), the China National Knowledge Infrastructure (CNKI), the Chinese Science and Technology Periodical Database (VIP), and the Wanfang Database from their inception to February 25, 2022 to include population-based, cross-sectional surveys that investigated the prevalence of depression in Chinese T2DM patients and studied possible risk factors. Gray literature and reference lists were also manually searched. We used the Agency for Healthcare Research and Quality methodology checklist to assess the risk of bias in the included studies. Two reviewers screened studies, extracted data, and evaluated the risk of bias independently. The primary outcome was the pooled prevalence of depression in Chinese T2DM patients, and the secondary outcomes included potential risk factors for depression in T2DM patients. R (version 3.6.1) and Stata (version 12.0) software were used for data synthesis.

**Results:**

We included 48 reports that identified 108,678 subjects. Among the included reports, 4 were rated as low risk of bias, 40 moderate risks of bias, and 4 high risks of bias. The prevalence of depression in T2DM patients in China was 25.9% (95% CI 20.6%−31.6%). The prevalence of depression was higher in women (OR = 1.36, 95% CI 1.19–1.54), subjects ≥60 years (OR = 1.56, 95% CI 1.14–2.14), with a primary school or lower education (*vs*. middle or high school education (OR = 1.49, 95% CI 1.16 – 1.92); *vs*. college degree or higher education (OR = 1.84, 95% CI 1.16 – 2.92), with a duration of T2DM ≥ 10 years (OR = 1.68, 95% CI 1.11–2.54), with complications (OR = 1.90, 95% CI 1.53–2.36), insulin users (OR = 1.46, 95% CI 1.09–1.96) and individuals living alone (OR = 2.26, 95% CI 1.71–2.98). T2DM patients with current alcohol use had a lower prevalence of depression (OR = 0.70, 95% CI 0.58–0.86). Prevalence varied from 0.8 to 52.6% according to different instruments used to detect depression.

**Conclusion:**

The prevalence of depression in T2DM patients is remarkable in China. Potential risk factors of depression in T2DM patients included women, age ≥ 60 years, low educational level, complications, duration of diabetes ≥ 10 years, insulin use, and living alone. High-quality epidemiological investigations on the prevalence of depression in Chinese T2DM patients are needed to better understand the status of depression in T2DM.

**Systematic Review Registration:**

https://www.crd.york.ac.uk/PROSPERO/, identifier: CRD42020182979.

## Introduction

Type 2 diabetes mellitus (T2DM) is a common chronic metabolic disease, accounting for 90%−95% of all cases (1). The highest proportions occur in low- and middle-income countries (2). According to the International Diabetes Federation (IDF) (3) and summary data by Ma (4), China has the largest diabetes population and is one of the countries with a sharp increase in diabetes prevalence worldwide, which is mainly attributed to T2DM (4). The latest statistics show that the overall prevalence of T2DM in China is 9.1% (5).

Depression refers to persistent sadness and a loss of interest or pleasure in previously rewarding or enjoyable activities (6). It prevails in T2DM patients, and numerous studies have suggested a bidirectional relationship between T2DM and depression (7–9). The association between the two conditions may cause a shared etiology, including alterations of the hypothalamic-pituitary-adrenal axis, inflammation, hippocampal structural alterations, and weight gain (7, 10). Nouwen et al. (11) report that T2DM patients have a 24% increased risk of developing depression compared to healthy individuals based on global data. Comorbid depression and T2DM appear to have an additive impact on patients, which results in poor compliance (12), worse quality of life (13), higher risk of dementia (14), and cardiovascular events (15, 16).

Globally, 28% of T2DM patients have depression of different degrees (17), and 14.5% have major depressive disorder (18). In developing countries, epidemiological studies show that depression in patients with T2DM ranges from 34 to 54% (19–21). Since China has a large T2DM population, the number of patients with T2DM and comorbid depression could be huge. Numerous cross-sectional surveys have investigated the prevalence of depression in Chinese T2DM patients, but the results vary. Although several studies have reported diverse risk factors associated with the prevalence of depression in T2DM patients, such as gender, age, educational level, complications, and lifestyle, the risk factors are inconsistent (22–24). A systematic review (SR) of the prevalence of depression in Chinese patients with T2DM has been conducted. This SR included 26 studies published up to 2016 (25). Since more surveys have been carried out in recent years, we conducted this SR and meta-analysis to update the current epidemiological data about the prevalence of depression in patients with T2DM in China and explore the potential risk factors associated with depression in patients with T2DM.

## Materials and Methods

### Study Registration

This SR adhered to the Preferred Reporting Items for Systematic reviews and Meta-Analysis (PRISMA) (26) and the Meta-analysis Of Observational Studies in Epidemiology (MOOSE) guidelines (27) (Supplementary File 1). The protocol was registered at the International Prospective Register of Systematic Reviews https://www.crd.york.ac.uk/PROSPERO/ (registration ID: CRD42020182979) and had been published (28).

### Search Strategy

We searched MEDLINE/PubMed, EMBASE, the Cochrane Library, the Chinese Biomedical Literature Database (CBM), the China National Knowledge Infrastructure (CNKI), the Chinese Science and Technology Periodical Database (VIP), and Wanfang Database from their inception to February 25, 2022, to collect the studies that reported the prevalence of depression in patients with T2DM in China. We combined Medical Subject Headings and free text words related to China, diabetes, and depression, to search the aforementioned electronic databases. Supplementary File 2 shows the search strategies of all databases. We manually searched gray literature, reference lists of included articles and relevant SRs for additional eligible studies. Relevant websites were also searched for useful data and we consulted experts for possible relevant studies.

### Study Selection

After filtering duplicate records, two independent reviewers (HLZ and JZ) screened the titles and abstracts of the remaining records for potentially eligible studies. Then, full texts were obtained and checked independently by two reviewers (XBL and XH). The studies were eligible if they met the following criteria:

Study characteristics: (1) Cross-sectional studies selecting subjects from the general population or community; (2) Reported the prevalence of depression in Chinese T2DM patients or/and investigated possible risk factors associated with depression in T2DM, regardless of sample size; (3) Published in English or Chinese.

Participant characteristics: (1) Chinese adults (≥18 years) diagnosed with T2DM with no restrictions on gender; (2) Diagnosis of T2DM based on self-reported physician's diagnosis, medical records, or glucose testing (fasting plasma glucose ≥ 7.0 mmol/L and/or 2-hour postprandial plasma glucose ≥ 11.1 mmol/L)(29); (3) Depression was evaluated with scales that have been verified with good validity and reliability in the Chinese population, or patients diagnosed as major depressive disorder by operationalized criteria such as the Diagnostic and Statistical Manual of Mental Disorders (DSM) or the International Statistical Classification of Diseases and Related Health Problems 10th Revision (ICD-10).

Type of outcome measurements: (1) The primary outcome was the pooled prevalence of depression in Chinese patients with T2DM; (2) Secondary outcomes contained potential risk factors of depression in patients with T2DM in China, such as gender, age, educational level, duration of T2DM, complications, insulin use, lifestyle, etc.

Hospital-based studies, randomized controlled trials, case studies, qualitative studies, systematic reviews, protocols, editorials, and conference abstracts were excluded. We also excluded duplicates. Studies were also excluded when the full text or data were unavailable by all useful approach.

### Data Extraction

Two reviewers (XBL and XH) independently extracted data using a predefined data extraction Microsoft Excel spreadsheet. The extracted data were:

(1) Study characteristics: title, journal, published year(s), survey period, geographical region, method of data collection, diagnostic criteria of diabetes, and instruments used to define depression, source of funding, and information regarding the risk of bias assessment.

(2) Participants' information at study level: mean age, gender, duration of diabetes, educational level, lifestyle (smoking status, alcohol use, living status), complications, insulin use, marital status (normal/abnormal [separated, divorced, widowed]).

(3) Critical data: sample size, the total number of subjects with depression, the number of subjects with depression according to age, gender, educational level, lifestyle, and other risk factors affecting prevalence.

We contacted the corresponding authors by e-mail for any incomplete information and data. The extracted data were cross-checked, and a third reviewer (JL) was invited to arbitrate disagreements.

### Risk of Bias Assessment

Two reviewers (YXL and DLZ) assessed the risk of bias of the included studies independently by the Agency for Healthcare Research and Quality (AHRQ) methodology checklist, which is devised for cross-sectional/prevalence studies (30). The AHRQ methodology checklist consists of 11 items. Each item is scored as “1” when answered “Yes” and “0” when answered “Unclear” or “No.” Studies are rated as high, moderate, and low risk of bias when quality is scored 0–3, 4–7, and 8–11, respectively. The results of the AHRQ methodology checklist were cross-checked, and disagreements were resolved by team discussion.

### Statistical Analysis

All statistical analyses were performed using R (vision 3.6.1) and Stata (vision 12.0) software. The pooled prevalence of depression in T2DM patients was calculated by the metaprop command in R (vision 3.6.1) software, with a 95% confidence interval (CI). We used odds ratio (OR) for categorical data (e.g., female and male, smoker and non-smoker) to explore potential risk factors. The statistical heterogeneity among studies was assessed by Cochran's *Q* test and the *I*^2^ statistic. A *P*-value <0.1 for the *Q* test and *I*^2^ >50% was set as the threshold for statistically significant heterogeneity. Since heterogeneity was expected, the random-effect model was used to pool all outcomes to give a more conservative estimate of prevalence. We used Kappa statistics to assess inter-rater agreement between reviewers for study inclusion and assessment of the risk of bias (31).

### Subgroup Analysis

Subgroup analyses were conducted according to age, residence, duration of T2DM, educational level, complications, insulin use, current smoking status, alcohol use, marital status, living status, and instruments used to detect depression.

### Publication Bias

Funnel plots and *Egger's* test were used to assessed the publication bias for primary outcome.

### Sensitivity Analysis

Sensitivity analysis was performed by excluding studies one by one to investigate the robustness of the results and pooling the studies' prevalence with a low, moderate, and high risk of bias, respectively, to explore the impact of studies' risk of bias on results. When asymmetric funnel plots indicated publication bias, we used the trim and fill method by metatrim packages of Stata (vision 12.0) software to quantify the impact of publication bias on results.

### Meta-Regression Analysis

Univariable meta-regression analysis was conducted to investigate potential sources of heterogeneity based on characteristics of study level (instruments used to detect depression, risk of bias, publication year, and sample size). Metareg command in R (vision 3.6.1) software was used for analyses.

## Results

### Included Studies and Characteristics

**Figure 1:**
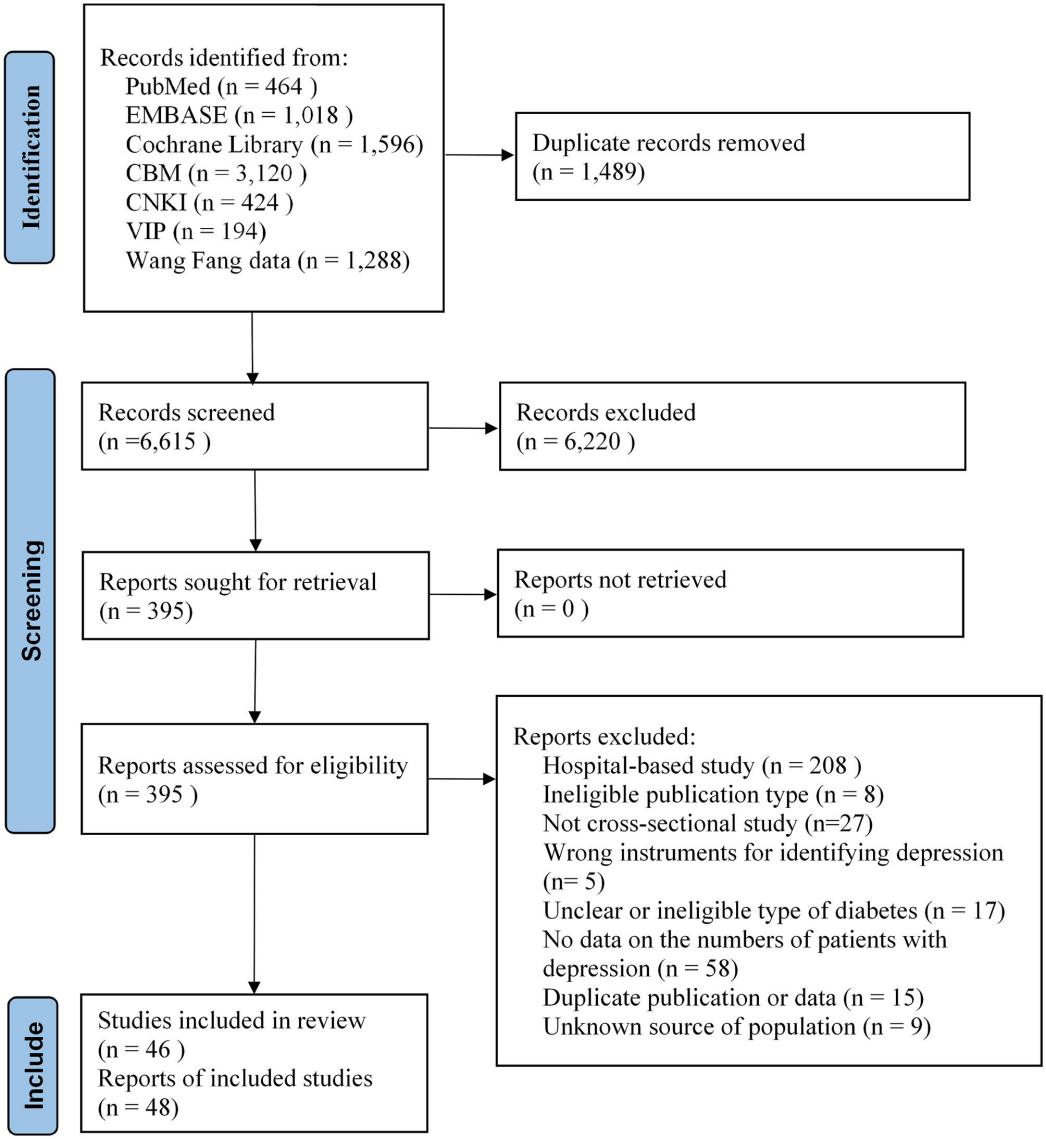
PRISMA flow diagram of the selection process.

A total of 8,104 articles were identified from the database and gray literature searches. After filtering 1,489 duplicates, we reviewed the titles and abstracts and identified 395 possible eligible records. Then, we checked the full texts and reference lists, and 48 reports (22–24, 32–76) fulfilled the eligible criteria. The list of excluded records with justification are provided in Supplementary File 3. Among the included reports, the sample of Sun et al. (22) and Zheng et al. (47) was a separate part of the sample of Zhang et al. (52) and Sun et al. (49). We used these as supplemental data for subgroup analysis. Inter-rater agreement between reviewers for study selection was excellent (Kappa statistics = 0.78). A PRISMA flow diagram of the selection process is presented in Figure 1.

The included studies contained 108,678 adult subjects. The ages of subjects ranged from 18 to 79 years. Three studies (23, 43, 49) were analyzed based on cross-sectional data from large national survey, and the rest were based on the population of different provinces and cities. Most of the studies evaluated depression status with the Self-rating Depression Scale, the Center for Epidemiologic Studies Depression Scale, the Patient Health Questionnaire-9, and the Geriatric Depression Scale. The prevalence of depression in T2DM patients reported in these studies ranged from 0.8% to 52.6%. Mezuk et al. (43) reported the lowest prevalence (0.8%), which included both diagnosed and undiagnosed T2DM in 10 provinces in China and those diagnosed with depression according to the DSM-IV. Zhang et al. (40) reported the highest prevalence (52.6%), which included elderly (≥60 years) T2DM patients and evaluated depression with the Beck depression inventory. Detailed characteristics of the included studies are shown in Table 1.

**Table 1:** Characteristics of included studies.

**References**	**Survey period**	**Sample size (n)**	**Gender (M/F)**	**Residence**	**Age mean (SD) / range**	**Duration of T2DM** **mean (SD) / range**	**Number of depression N (%)**	**Instruments used to detect depression**
Chou and Chi (32)	1996	246	102/144	-	70 ± 7.1	-	64 (26.0%)	GDS-15 ≥ 8
Xu (33)	2006.04-2006.12	217	83/134	-	61.4 ± 8.8	-	46 (21.2%)	CESD-15 ≥ 16
Huang et al. (34)	2007.08-2007.11	323	146/177	Urban area; rural area	68.63 ± 6.48	-	83 (25.7%)	CESD-15 ≥ 16
Sun and Dong (35)	-	440	189/251	Urban area	58 ± 8	5.0 ± 4.5	96 (21.8%)	SDS ≥ 50
Chen et al. (36)	2002	150	54/96	-	59 ± 10	-	29 (19.3%)	SDS ≥ 50
Liu et al. (37)	2009.06-2009.09	619	-	Urban area	61.36 ± 10.93	7.65 ± 6.31	273 (44.1%)	SDS ≥ 53
Qian et al. (38)	-	110	46/64	-	-	-	35 (31.8%)	HAMD-14 ≥7
Yang et al. (39)	2010.08-2010.09	120	56/64	Urban area; rural area	55 ± 14	6.2 ± 4.3	52 (43.3%)	SDS ≥ 50
Zhang (40)	2010.08-2010.11	154	81/73	-	68.0 ± 5.3	8.9 ± 6.8	81 (52.6%)	BDI ≥ 5
Wang (41)	-	360	160/200	-	61.4 ± 8.26	-	110 (30.6%)	CESD-15 ≥ 16
Liu et al. (42)	2009.07-2010.03	667	319/348	-	61.81 ± 10.75	7.20 ± 6.34	295 (44.2%)	SDS ≥ 53
Mezuk et al. (43)	2004-2008	26,509	10,386/16,123	Rural area; urban area	-	-	206 (0.8%)	CIDI-SF
Wang et al. (44)	2012.04-2012.05	755	340/415	-	-	-	117 (15.5%)	SDS ≥ 53
Xie (45)	-	1,606	1,012/594	-	59.53 ± 13.79	-	605 (37.7%)	SDS ≥ 50
Xu et al. (46)	-	100	-	Rural area	-	-	42 (42.0%)	SDS ≥ 50
Zheng et al. (47)	2012.03-2012.11	2,511	0/2511	Urban area	60.2 ± 8.5	5.26 ± 4.6	228 (9.1%)	PHQ-9 ≥ 5
Wang et al. (48)	-	865	403/462	Urban area	70.13 ± 20.33	9.00 ± 7.05	304 (35.1%)	SDS ≥ 53
Sun et al. (49)	2011-2012	49,077	19,678/29,399	Rural area; urban area	-	-	3,110 (6.3%)	PHQ-9 ≥ 5
Sun et al. (22)	2013.08-2013.12	893	370/523	-	63.9 ± 10.2	5.6 ± 5.1	389 (43.6%)	SDS ≥ 50
Li et al. (50)	2014	704	244/460	Urban area	65.7 ± 10.3	-	305 (43.3%)	SDS ≥ 50
Ning et al. (51)	2006.02-2006.05	489	206/283	Urban area; rural area	51 ± 10.6	-	69 (14.1%)	SDS ≥ 50
Zhang et al. (52)	2012.11-2013.01	979	402/577	Rural area; urban area	62.9 ± 10.2	5.9 ± 5.1	401 (41.0%)	SDS ≥ 50
Huang et al. (53)	2013.06-2014.06	468	-	-	-	-	231 (49.4%)	SDS ≥ 50
Li et al. (54)	2014.03-2014.12	272	118/154	Urban area	71.0 ± 6.1	37.8 ± 8.5	49 (18.0%)	PHQ-9 ≥ 10
Ni and Liu (55)	2012	3,280	1,478 /1,802	Urban area	70.16 ± 10.04	-	614 (18.7%)	PHQ-9 ≥ 5
Yang et al. (56)	2016.03-2016.04	242	122/120	-	-	-	112 (46.3%)	SDS ≥ 50
Liu et al. (23)	2008-2011	2,399	1,134/1,263	Urban area; rural area	60.1	-	938 (39.1%)	CESD-10 ≥ 10
Li et al. (24)	2012.01-2013.08	1,221	535/686	Rural area	-	-	386 (31.6%)	PHQ-9 ≥ 5
Lee et al. (57)	2010.03-2012.08	696	290/406	Rural area	68.2 ± 9.5	8.9 ± 6.6	117 (16.8%)	GDS-15>7
Li et al. (58)	-	109	-	Rural area	-	-	54 (49.5%)	PHQ-9 ≥ 7
Tang et al. (59)	2017	967	410/557	-	67.97 ± 5.52	-	187 (19.3%)	SDS ≥ 50
Fu et al. (60)	-	1,203	518/685	Urban area	70.48 ± 10.16	-	587 (48.8%)	SDS ≥ 53
Ren (61)	2011.09-2011.12	975	426/549	-	52.71 ± 12.83	5.74 ± 3.13	211 (21.6%)	SDS ≥ 50
Sun et al. (62)	2016.10-2017.10	280	113/167	-	70.56 ± 6.68	-	75 (26.8%)	GDS ≥ 11
Zhang et al. (63)	2016.01-2016.04	337	214/123	-	55.5 ± 11.0	-	16 (4.7%)	PHQ-9 ≥ 5
Zhang and Zhang (64)	-	196	-	-	-	-	101 (51.5%)	HAMD-24 ≥ 8
Xiu et al. (65)	2009.07-2009.11	2,626	973/1,653	Urban area; rural area	71.21 ± 7.41	-	285 (10.9%)	GDS-15 ≥ 6
Zhang et al. (66)	2019.06-2019.08	224	104/120	-	67.3 ± 6.6	-	44 (19.6%)	ZSDS ≥ 53
Zhang et al. (67)	2019.02-2019.06	319	147/172	-	74.3 ± 6.7	-	144 (45.1%)	GDS-15 ≥ 6
Xu et al. (68)	2014.03-2017.05	676	-	-	-	-	15 (2.2%)	PHQ-9 ≥ 10
Abdulai et al. (69)	2015.07-2017.09	2,776	-	Rural area	18–79	-	178 (6.4%)	PHQ-2 ≥ 3
Kong et al. (70)	2019.06-2019.10	291	137/154	-	69–72	-	63 (21.6%)	GDS-15 ≥ 6
Gao et al. (71)	-	718	347/371	-	-	-	161 (22.4%)	PHQ-8 ≥ 5
Pan et al. (72)	2018.11-2019.04	1,370	538/832	Urban area; rural area	65.1 ± 7.7	-	370 (27.0%)	PHQ-9 ≥ 5
Wu et al. (73)	2019.01-2019.12	308	161/147	Urban area	62.9 ± 7.6	-	129 (41.9%)	SDS ≥ 53
Yang and Wu (74)	2021.03-2021.06	389	0/389	-	72.72 ± 6.09	6–15	54 (13.9%)	GDS-15 ≥ 5
Liu et al. (75)	2017-2019	1,684	-	Urban area; rural area	-	-	107 (6.4%)	DASS-21
Ji et al. (76)	2018.05-2018.08	162	74/88	Urban area	69.0 ± 7.2	10.5 ± 8.0	30 (18.5%)	PHQ-9 ≥ 5

*-, No report; CESD, Center for Epidemiologic Studies Depression Scale; SDS, Self-Rating Depression Scale; HAMD, Hamilton Depression Rating Scale; CIDI-SF, Chinese version of the computerized Composite International Diagnostic Inventory- short form; PHQ, Personal Health Questionnaire; GDS, Geriatric Depression Scale; DASS-21, the 21-item Depression Anxiety Stress Scales; UD, undiagnosed diabetes*.

### Risk of Bias

Among the included reports, the AHRQ scores ranged from two to nine. Four reports were rated as low risk of bias, 40 moderate risks of bias, and 4 high risks of bias. Agreement of the risk of bias assessment was excellent (Kappa statistics = 0.8). Supplementary File 4 provides detailed scores of all included studies.

## Meta-Analysis

### Prevalence of Depression in Patients With T2DM in China

**Figure 2:**
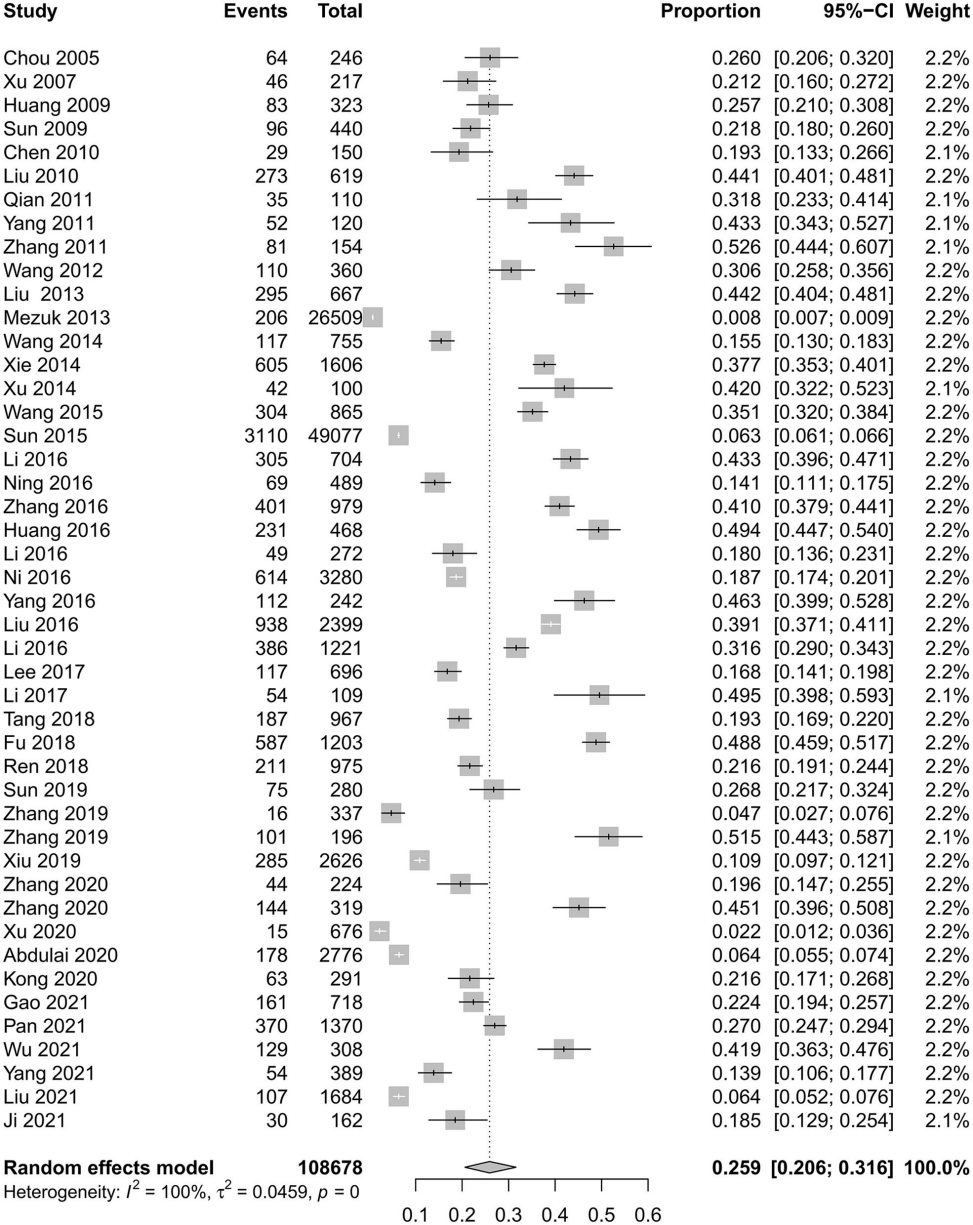
Forest plot of the prevalence of depression in patients with T2DM in China.

**Figure 3:**
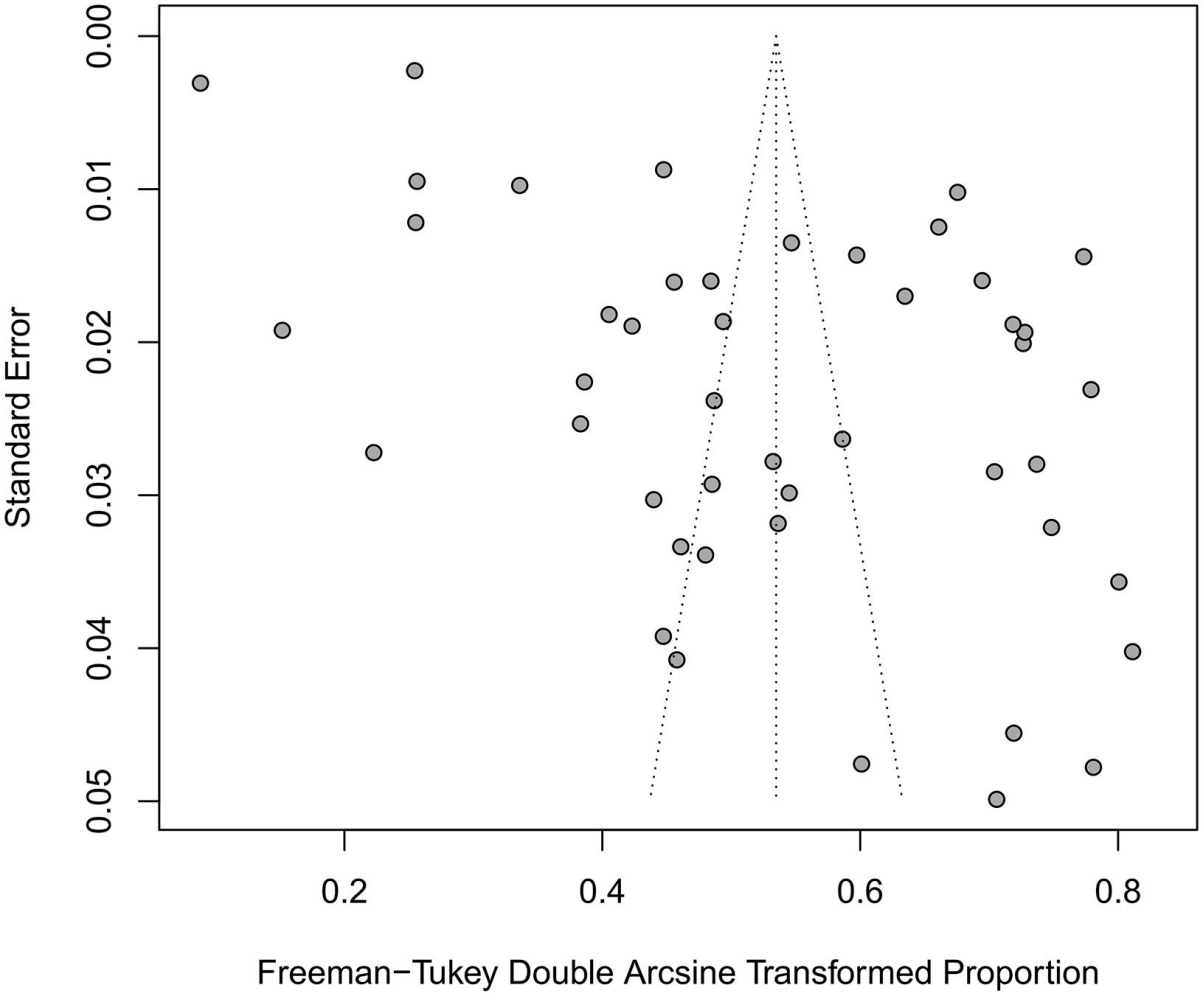
Funnel plot of the prevalence of depression in patients with T2DM in China.

The prevalence of depression in patients with T2DM in China was 25.9% (95% CI 20.6%−31.6%), with statistically significant heterogeneity between studies (*I*^2^ = 99.7%, *P* < 0.0001) (Figure 2). Asymmetric funnel plot indicated publication bias might exist (Figure 3) (*Egger's test, P* < 0.0001).

### Prevalence of Depression in Patients With T2DM According to Gender

Nineteen studies reported the prevalence of depression in patients with T2DM according to gender. Overall, the pooled prevalence of depression in women with T2DM was higher than in men (33.4% [95% CI 24.6%−42.2%; *I*^2^ = 99.2%, *P* < 0.0001] *vs*. 28.0% [95% CI 18.0%−39.1%; *I*^2^ = 99.3%, *P* < 0.0001]). The OR in women compared with men was 1.36 (95% CI 1.19–1.54; *I*^2^ = 64.3%, *P* < 0.0001).

### Prevalence of Depression in Patients With T2DM According to Age

We dichotomized the reported age groups into <60 years and ≥60 years. Overall, the data of subjects ≥60 years was extracted from 18 studies, and <60 years from 7 studies. The pooled prevalence of depression in patients with T2DM was higher in subjects aged ≥60 years compared with those aged <60 years (29.5% [95% CI 24.6%−35.4%; *I*^2^ = 96.1%, *P* < 0.0001] *vs*.23.7% [95% CI 15.7%−31.8%; *I*^2^ = 95.2%, *P* < 0.0001]). The prevalence of depression in patients with T2DM aged ≥60 years was significantly higher than those aged <60 years (OR = 1.56; 95% CI 1.14–2.14; *I*^2^ = 78.5%, *P* < 0.0001).

### Prevalence of Depression in Patients With T2DM According to Educational Level

We divided education into three levels (primary school or lower education level; middle or high school; a college degree or higher education level). The prevalence of depression in patients with T2DM with primary school or lower education level was 34.0%, middle or high school education 24.2%, and a college degree or above education level 24.6%. In these studies, the prevalence of depression in patients with T2DM increased significantly with low education level (Table 2).

**Table 2:** Pooled prevalence of depression in patients with T2DM according to educational level.

**Educational level band**	**Number of studies (n)**	**Number of** **subjects (n)**	**Pooled prevalence of depression in T2DM (95% CI)**	**Heterogeneity**		**Odds ratio for depression in T2DM (95 % CI)**	**Heterogeneity**	
				***I*** ^***2***^	***P***		***I*** ^***2***^	***P***
Primary school or lower education	10	2,984	34.0% (26.9%−41.8%)	92.7%	<0.0001	1.0	_	_
Middle or high school education	9	3,246	24.2% (16.8%−33.5%)	97.0%	<0.0001	1.49 (1.16–1.92)^*^	51.7%	0.0431
College degree or higher education	11	1,349	24.6% (17.7%−34.1%)	91.6%	<0.0001	1.84 (1.16–2.92)^#^	70.7%	0.0012

* *Primary school or lower education vs. Middle or high school education*.

# *Primary school or lower education vs. College degree or higher education*.

### Prevalence of Depression in Patients With T2DM According to Residence

Eleven studies included urban residence. The pooled prevalence of depression in patients with T2DM in urban residents was 27.6% (95% CI 20.2%−36.4%; *I*^2^ = 99.0%, *P* < 0.0001). Six studies included rural residents. The pooled prevalence of depression in patients with T2DM in rural residents was 33.3% (95% CI 15.6%−50.9%; *I*^2^ = 99.6%, *P* < 0.0001). Due to incomparable data, the OR was not calculated.

### Prevalence of Depression Between Undiagnosed and Diagnosed T2DM Patients

Seven studies included undiagnosed T2DM patients (those without a history of physician-diagnosed diabetes or use of an antidiabetic drug but with a fasting plasma glucose of 7.0 mmol/L or higher, or a 2-hour postprandial plasma glucose of 11.1 mmol/L or higher). The prevalence of depression in undiagnosed T2DM was 7.3% (95% CI 2.6%−19.1%; *I*^2^ = 99.7%, *P* < 0.0001). The pooled results from 44 included studies showed prevalence of depression in diagnosed patients with T2DM was 27.1% (95%CI 21.4–33.1%; *I*^2^ = 99.6%, *P* < 0.0001).

### Prevalence of Depression in T2DM Patients According to the Duration of T2DM

Seven studies reported the prevalence of depression in patients with T2DM according to the duration of diabetes. The duration of diabetes from these studies was divided into three groups: <5 years, 5–10 years, ≥10 years. The prevalence of depression in T2DM with <5 years was 26.3%; in T2DM with 5–10 years was 28.9%; in T2DM with ≥10 years was 29.2%. The prevalence of depression in T2DM patients increased significantly with longer duration of T2DM in these studies (Table 3).

**Table 3:** Pooled prevalence of depression in patients with T2DM according to duration of T2DM.

**Duration of T2DM band**	**Number of studies (n)**	**Number of** ** subjects** ** (n)**	**Pooled prevalence of depression in T2DM (95% CI)**	**Heterogeneity**		**Odds ratio for depression in T2DM (95 % CI)**	**Heterogeneity**	
				***I*** ^***2***^	***P***		***I*** ^***2***^	***P***
<5 years	7	1,122	26.3% (19.2%−36.1%)	88.9%	<0.0001	1.00	_	_
5–10 years	5	404	28.9% (16.9%−44.7%)	90.4%	<0.0001	1.12 (0.83–1.51)	0.0%	0.6720
≥10 years	6	786	29.2% (16.7%−51.2%)	96.3%	<0.0001	1.68 (1.11–2.54)	55.0%	0.0642

### Prevalence of Depression in Patients With T2DM According to Insulin Use

The pooled prevalence of depression in patients with T2DM was higher in subjects using insulin compared with those not using insulin (49.5% [95% CI 44.0 %−55.0%; *I*^2^ = 51.8%, *P* = 0.0654] *vs*. 38.6% [95% CI 30.4%−46.7%; *I*^2^ = 91.9%, *P* < 0.0001). The OR in insulin users compared with non-insulin users was 1.46 (95% CI 1.09–1.96; *I*^2^ = 51.4%, *P* = 0.0673).

### Prevalence of Depression in Patients With T2DM According to Complications

The pooled prevalence of depression in patients with T2DM with complications was higher in subjects without complications (47.3% [95% CI 39.8%−54.8%; *I*^2^ = 84.7%, *P* < 0.0001] *vs*. 31.1% [95% CI 23.2%−38.9%; *I*^2^ = 92.8%, *P* < 0.0001]), with an OR of 1.90 (95% CI 1.53–2.36; *I*^2^ = 39.4%, *P* = 0.1429).

### Prevalence of Depression in Patients With T2DM According to Current Smoking Status

The pooled prevalence of depression in patients with T2DM with current smoking status was 24.4% (95% CI 16.4%−34.8%; *I*^2^ = 91.4%, *P* < 0.0001) and in non-smokers 25.8% (95% CI 16.7%−37.6%; *I*^2^ = 99.1%, *P* < 0.0001). There was no difference in prevalence of depression between current smoking T2DM and non-smoker (OR=0.85, 95% CI 0.60–1.20; *I*^2^ = 78.0%, *P* = 0.0001).

### Prevalence of Depression in Patients With T2DM According to Current Alcohol Use

The pooled prevalence of depression in patients with T2DM with current alcohol use was 21.6% (95% CI 13.8%−30.4%; *I*^2^ = 86.2%, *P* < 0.0001), while prevalence of depression in non-drinkers was 23.5% (95% CI 14.9%−35.1%; *I*^2^ = 99.1%, *P* < 0.0001). The OR for T2DM with current alcohol use vs. non-drinkers was significantly lower (OR=0.70, 95% CI 0.58–0.86; *I*^2^ = 15.7%, *P* = 0.3129).

### Prevalence of Depression in Patients With T2DM According to Marital Status

The pooled prevalence of depression in patients with T2DM who had abnormal marital status (separated, divorced, widowed) was 38.5% (95% CI 27.4%−49.6%; *I*^2^ = 91.4%, *P* < 0.0001); for normal marital status was 31.4% (95% CI 23.6%−39.1%; *I*^2^ = 97.2%, *P* < 0.0001). The OR in T2DM with abnormal marital status compared with normal marital status was 1.39 (95% CI 0.90–2.14; *I*^2^ = 83.5%, *P* < 0.0001).

### Prevalence of Depression in Patients With T2DM According to Living Status

The pooled studies prevalence of depression in patients with T2DM who were living alone was higher compared to those not living alone (50.3% [95% CI 40.1%−60.5%; *I*^2^ = 62.1%, *P* = 0.0418] *vs*.29.4% [95% CI 19.7%−41.3%; *I*^2^ = 94.8%, *P* < 0.0001]). There was significant difference in prevalence of depression between patients with T2DM who were living alone and those not living alone (OR = 2.26, 95% CI 1.71–2.98; *I*^2^ = 0.0%, *P* = 0.7581).

### Prevalence of Depression in Patients With T2DM According to Different Instruments Used to Detect Depression

The included studies used various instruments to detect depression; there were different cut-off points even for the same instrument. Table 4 presents the pooled prevalence of depression in patients with T2DM according to the various instruments and cut-off points.

**Table 4:** Pooled prevalence of depression in patients with T2DM according to instruments used to evaluated depression.

**Methods used to define depression**	**Studies (n)**	**Subjects (n)**	**Pooled prevalence (%)**	**95% CI (%)**	***I*** ***^2^ (%)***	***P*** **Value for** ***I***^***2***^
**SDS**						
≥50	12	7,240	32.5%	25.6%−39.8%	97.6%	<0.0001
≥53	7	4,641	35.6%	24.7%−46.5%	97.9%	<0.0001
**PHQ-9**						
≥5	7	55,556	20.3%	10.8%−31.9%	99.6%	<0.0001
≥10	2	948	10.0%	0.00%−25.4%	97.7%	<0.0001
PHQ-2	1	2,776	6.4%	-	-	-
PHQ-8 ≥ 5	1	718	22.4%	-	-	-
CESD-20 ≥ 16	3	900	26.0%	21.0%−31.3%	68.7%	0.0411
CESD-10 ≥ 10	1	2,399	39.1%	-	-	-
**GDS-15**						
≥5	1	389	13.9%	-	-	-
≥6	3	3,236	22.0%	8.3%−58.3%	99.3%	<0.0001
≥8	2	942	21.1%	12.1%−30.1%	88.4%	0.0033
GDS-30 ≥ 11	1	280	26.8%	-	-	-
HAMD-14 ≥ 7	1	110	31.8%	-	-	-
HAMD-24 ≥ 8	1	196	51.5%	-	-	-
BDI ≥ 5	1	154	52.6%	-	-	-
CIDI-SF	1	26,509	0.8%	-	-	-
DASS-21	1	1,684	6.4%	-	-	-

*CESD, Center for Epidemiologic Studies Depression Scale; SDS, Self-Rating Depression Scale; HAMD, Hamilton Depression Rating Scale; the CIDI-SF, Chinese version of the computerized Composite International Diagnostic Inventor- short form; PHQ, Personal Health Questionnaire; GDS, Geriatric Depression Scale; UD, undiagnosed diabetes; DASS-21, the 21-item Depression Anxiety Stress Scales*.

### Sensitivity Analysis

**Figure 4:**
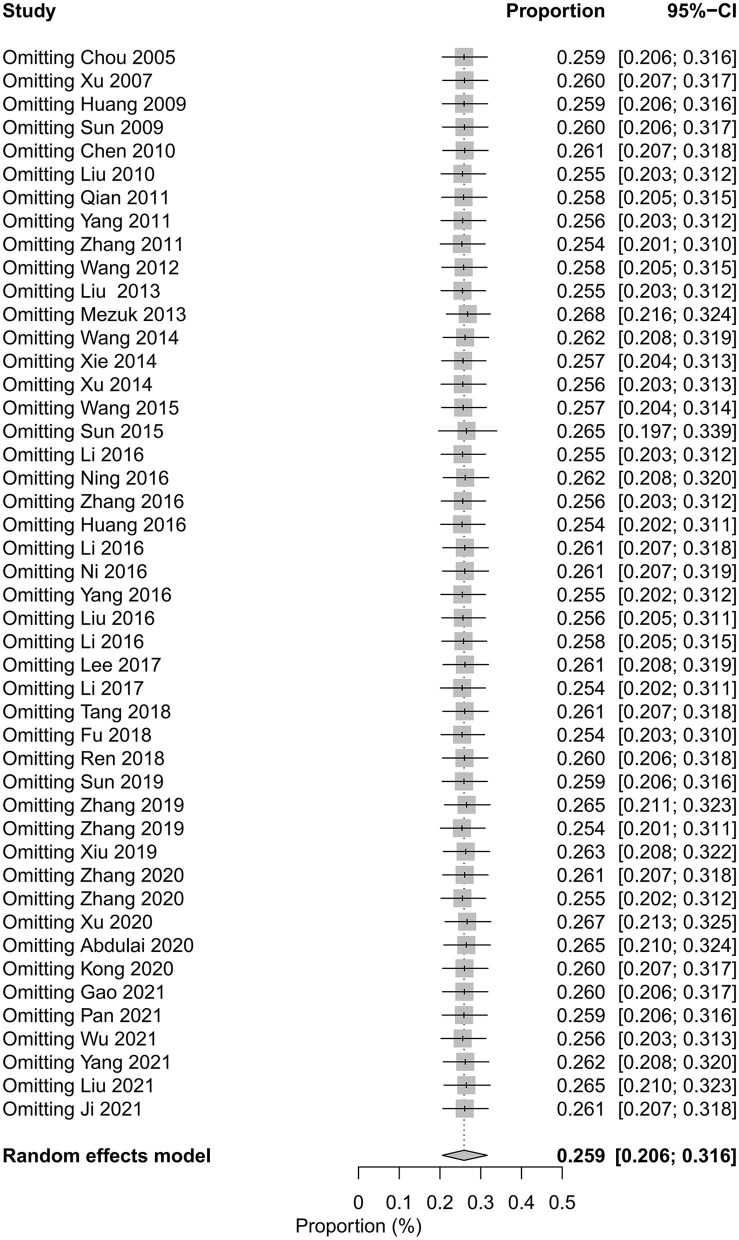
Sensitivity analysis by excluding studies one by one.

**Figure 5:**
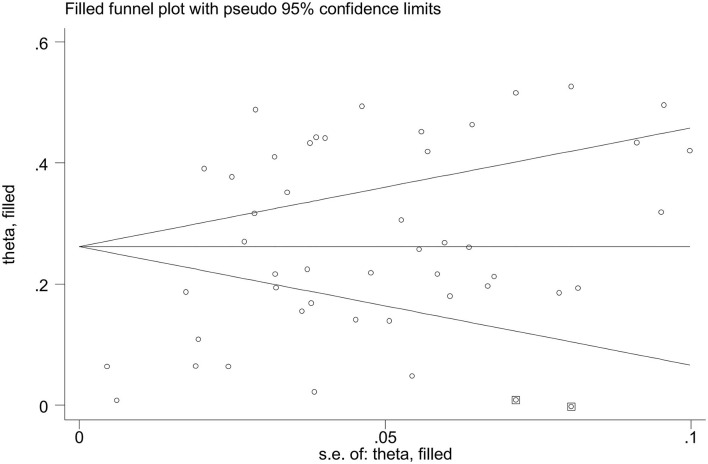
Funnel plot after applying trim and fill method.

The pooled prevalence of depression in patients with T2DM varied from 25.4%−26.8% after excluding studies one by one (Figure 4). We applied the trim and fill method, after filling 2 estimated missing studies, the funnel plot was symmetrical around the adjusted effect size (Figure 5). The adjusted results showed that the prevalence of depression in T2DM patients was 26.2% (95% CI 21.9%−30.5%).

We categorized the prevalence of studies with low, moderate, and high risk of bias, respectively, and the results showed that the prevalence of depression in patients with T2DM was 6.9% (95% CI 2.3%−13.6%; *I*^2^ = 99.9%, *P* < 0.0001), 27.0% (95% CI 22.5%−31.8%; *I*^2^ = 98.7%, *P* < 0.0001), and 40.4% (95% CI 23.9%−56.9%; *I*^2^ = 97.6%, *P* < 0.0001).

### Meta-Regression Analysis

The results of univariate meta-regression showed the risk of bias of included studies and instruments used to define depression were the source of heterogeneity (Table 5).

**Table 5:** Result of univariable meta-regression.

	**Number of data points**	**Regression coefficient (95% CI)**	**Standard error**	***P***
Publication year	46	−0.0086 (−0.0235 to 0.0063)	0.0076	0.2553
**Risk of bias**				
Low	4	1	-	-
Moderate	38	−0.1379 (−0.2828 to 0.0071)	0.0739	0.0623
High	4	−0.4179 (−0.6106 to −0.2252)	0.0983	<0.0001
**Scales**				
BDI	1	1	-	-
GDS	7	−0.3202 (−0.6255 to −0.0149)	0.1558	0.0398
SDS	19	−0.1942 (−0.4877 to 0.0993)	0.1498	0.1948
PHQ	11	−0.3924 (−0.6910 to −0.0939)	0.1523	0.0100
HAMD	2	−0.1081 (−0.4592 to 0.2431)	0.1792	0.5464
CIDISF	1	−0.7228 (−1.1202 to −0.3255)	0.2027	0.0004
CESD	4	−0.2413 (−0.5601 to 0.0775)	0.1626	0.1379
DASS21	1	−0.5559 (−0.9539 to −0.1579)	0.2031	0.0062
**Sample size**				
≥1000	11	1	-	-
<1000	35	0.1191 (−0.0063 to 0.2445)	0.0640	0.0627

## Discussion

This SR and meta-analysis synthesized data from 48 population-based cross-sectional surveys that reported the prevalence of depression in T2DM patients in China. The results showed that 25.9% of T2DM patients in China suffered from different degrees of depression. Prevalence varied strikingly, from 7.3% to 50.3%, according to the demographic features in the study. A higher prevalence of depression existed in patients with T2DM who were women, aged ≥60 years, with primary school or lower education level and duration of diabetes ≥10 years, using insulin, with complications, living alone and living in rural areas. The prevalence of depression in undiagnosed T2DM was distinctly low. Alcohol users with T2DM had a lower prevalence of depression; other demographic features, like current smoking status and abnormal marriage, were unrelated to depression in patients with T2DM.

A global statistic calculated by Khaledi et al. (17) pooled 248 studies up to 2018. They reported that the global prevalence of depression in patients with T2DM was 28%. In their report, they included patients identified with depression through self-report or validated instruments. Another meta-analysis (18) showed that 14.5% patients with T2DM globally were diagnosed as major depressive disorder based on operationalized criteria, such as the DSM and ICD-10. Wang et al. (25) included 26 studies published up to 2016, 66,475 subjects, and reported that 28.9% of T2DM patients in China suffered from depression. In our study, we included 48 sudies with 108,678 T2DM subjects. The prevalence of depression in patients with T2DM was 25.9% in China. For the reason of such high prevalence of depression in T2DM, the researchers inferred that the link between depression and diabetes was associated with Gene-Environment interaction. The patients may share biological pathways contributing to pathogenesis of depression and T2DM, such as dysregulation of hypothalamic–pituitary–adrenal axis, overactivation of innate immunity, and inflammatory response (10, 77, 78).

Our study showed that the prevalence of depression in patients with undiagnosed T2DM (7.3%) was notably lower than diagnosed T2DM (27.1%), which was consistent with previous studies. A meta-analysis (79) found, compared with individuals without diabetes, undiagnosed diabetes have a slight risk of depression (OR = 1.27), while diagnosed diabetes have a higher risk of depression (OR = 1.80). Another study showed that the risk of depression does not differ between individuals with undiagnosed diabetes and individuals with normal glucose metabolism (80). The evidence mentioned above suggested awareness of diabetes may induce depression in patients with T2DM; therefore, early psychosomatic management is necessary.

We found that women with T2DM had a higher prevalence of depression than men. Many epidemiological studies pointed out the gender gap in the prevalence of depression—women are about twice as likely to suffer from depression than men during their lifetime (81, 82), which may be contributed to environmental, hormonal, genetic factors. In addition, among patients with T2DM, depressive symptoms are related to higher HbA1c levels (83), and the depressive symptoms was positively associated with HbA1c levels in female patients with T2DM (84). The above factors might lead to the gender difference in the prevalence of depression in T2DM patients observed in our and some other relevant meta-analyses (17, 19, 20, 25).

Few meta-analyses focus on the influence of educational characteristics on depression in patients with T2DM. Several large observational studies discovered the same trend with our study (85, 86) that a low educational level was a risk factor for depression in T2DM patients. A low educational level may lead to a low socioeconomic status (87), such as insufficient social support and medical resources, and poor perception of health, which may further lead to poor blood glucose control and a high risk of depression in patients with T2DM.

We found the risk of depression in elderly patients ≥60 years was 1.56 times higher than those <60 years. However, Khaledi et al. (17) reported that depression was more common in T2DM patients under 65 years than in the elderly. Their study pooled global data from cross-sectional, case-control, and cohort studies that reported the current or lifetime prevalence of depression. In our study, we included cross-sectional studies and reported the current prevalence of depression. The epidemiological data of China showed that the middle-aged and elderly patients were the mainstream population with T2DM, and the prevalence of T2DM increased rapidly with age (65-74 years old, 14.1%; 55–64 years old, 11.0%; over 75 years old, 11.0%) (5, 88). Therefore, the majority of patients with T2DM included in our studies were around 60 years old, though our inclusion criterion was population with T2DM aged over 18 years. We presumed that, with the decline of health level and prolonged duration of T2DM, elderly patients with T2DM are more likely to suffer from multiple diseases and diabetes complications, which may contribute to a high risk of depression.

Our studies found that patients with a duration of T2DM more than 10 years had a higher prevalence of depression. Wang et al. (25) reported that a one-year increment in diabetes duration led to a 1.1-fold increase in the risk of depression. Previous studies found long duration of diabetes was associated with a high risk of microvascular diseases (89, 90), such as nephropathy, retinopathy, neuropathy, and macrovascular complications (91). Meanwhile, the duration of T2DM varies inversely with quality of life, especially in patients with diabetes duration of more than 10 years (13).

Complications cause a greater medical burden and worse health for T2DM patients. Together with Wang et al. (25) (RR = 2·08), and Hussain et al. (19) (OR = 2.33), we (OR = 1.90) found that diabetes complications increased almost two-fold risk of depression. Rees et al. (92) reported that severe non-proliferative or proliferative diabetic retinopathy was independently associated with depressive symptoms. Due to inadequate raw data, we failed to investigate the association between types and numbers of complications and depression. More studies are needed to explore the relationship between diabetes complications and depression.

Our results showed that T2DM using insulin had higher risk of depression than non-insulin user. Consistent with our result, a previous meta-analysis (93) confirmed that insulin therapy increased the risk of depression in T2DM patients comparing with non-insulin users or oral antidiabetic drug users. Insulin is usually prescribed to treat diabetic patients with poor glycemic control, especially those with advanced diabetes (94). Besides, T2DM patients with insulin therapy commonly suffered from negative physical side effects, including hypoglycaemia, weight gain and pain. Some patients even experienced psychological barriers associated with insulin injections, such as they felt that insulin injections made their life less flexible and symbolized a worsening condition, and they concerned about other people's negative reactions (95). In other words, due to insufficient physical and psychological adjustment, T2DM patients using insulin are more likely to suffer from depression (96).

Regarding lifestyle, we found several notable factors that affected depressive symptoms in T2DM patients. We observed that T2DM patients living alone had a more than two-fold higher risk for depression. Researchers concluded that living alone was supposed to be an independent risk factor for depression (97–99). Subjects living alone were more likely to experience insomnia (100), have low social support, and social isolation. Besides, due to the one-child policy during 1980–2016, and the rapidly aging population in China, living alone in the elderly, also called empty-nest, became prevalent (101). Besides, we found that alcohol users had a significantly lower prevalence of depression among T2DM patients. Previous studies have shown that moderate alcohol consumption brought better mental health conditions and lower levels of depression (102, 103). A recent pooled analysis also suggested that (104) low-to-moderate alcohol intake was associated with a significantly reduced risk of long-term depressive symptoms. However, due to limited data in our studies and difference in patients' health conditions, we should be cautious about this finding. Future studies are needed to investigate the relationship between moderate alcohol consumption and the mental condition of T2DM patients.

The results of sensitivity analysis and meta-regression in our study showed that the risk of bias might be the source of heterogeneity, and studies with low risk of bias were associated with lower prevalence. We noticed that most of included studies were rated as high risk of bias on items involving explanation of patient exclusions, assessments undertaken for quality assurance purposes, handling of missing data, and assessment and control of confounding. Thus, researchers should pay attention to these issues to reduce the risk of bias of cross-sectional studies. In addition, results of subgroup analysis suggested that the prevalence were varied according to different instruments used to detect depression, and sensitivity analysis showed that instruments were the source of heterogeneity. Researchers found that most of depression screening instruments showed moderate–good sensitivity and specificity in detecting depression in patients with T2DM, but often produced high false positive rate (105). The symptoms of T2DM and its long-term complications including tiredness, lethargy, lack of energy, sleeping difficulties and appetite changes, may overlap with symptoms in depression. Therefore, the specific depression screening tools for patients with T2DM should be developed, which may provide more accurate prevalence of depression in patients with T2DM.

### Limitations

There are some limitations existed in the present study. Firstly, various identification instruments of depression were used in cross-sectional studies, and the results of subgroup analysis showed that the prevalence were varied from different instruments, which may have impact on the pooled prevalence. Second, we calculated ORs using raw data from the studies rather than adjusting for potential underlying differences among study participants, the residual confounding was inevitable. Third, we found significant heterogeneity existed, to explore the source of heterogeneity, we conducted the meta-regression, and the results showed that different instruments used to detect depression and the risk of bias were the source of heterogeneity. Fourth, since we included the cross-sectional studies, and extracted the characteristics at study level to explored the potential risk factors, the ecological fallacy may exist.

## Conclusion

In conclusion, this SR and meta-analysis suggested a pooled prevalence of depression in patients with T2DM in China was 25.9%. Potential risk factors of depression in patients with T2DM might include female gender, age ≥60 years, low educational level, complications, diabetes duration ≥10 years, insulin use, and living alone. High-quality epidemiological surveys of the prevalence of depression in T2DM in China are needed to better understand the status of depression for T2DM.

## Data Availability Statement

The original contributions presented in the study are included in the article/Supplementary Material, further inquiries can be directed to the corresponding author/s.

## Author Contributions

XL, YL, and LG wrote the manuscript. RJ, JL, and DZ conceptualized the study and provide methodological support. YL and LG designed the search strategy and performed searches. HZ, JZ, XL, and XH selected studies. XL and XH extracted the data. YL and DZ assessed the risk of bias of included studies. All authors contributed to the article and approved the submitted version.

## Funding

This work was supported by the National Natural Science Foundation of China (Number: 81873356), National Key R&D Program of China (Number: 2019YFC1710302), and Sichuan Province Science and Technology Support Program in Sichuan (Number: 2014SZ0154).

## Conflict of Interest

The authors declare that the research was conducted in the absence of any commercial or financial relationships that could be construed as a potential conflict of interest.

## Publisher's Note

All claims expressed in this article are solely those of the authors and do not necessarily represent those of their affiliated organizations, or those of the publisher, the editors and the reviewers. Any product that may be evaluated in this article, or claim that may be made by its manufacturer, is not guaranteed or endorsed by the publisher.

## Supplementary Material

Supplementary File 1 PRISMA and MOOSE checklist.

Supplementary File 2 Search strategy of all databases.

Supplementary File 3 List of excluded records by screening full text.

Supplementary File 4 Risk of bias assessment results of included studies.

Supplementary File 5 Forest plot and funnel plot of subgroup analysis.
