# Supplementary material for: A Systematic Review and Meta-Analysis of the Prevalence and Risk Factors of Depression in Type 2 Diabetes Patients in China
Source: Front Med (Lausanne). 2022 May 10;9:759499. doi: 10.3389/fmed.2022.759499 (PMC9127805; doi:10.3389/fmed.2022.759499)
Supplement: Supplementary File 2 — Search strategy of all databases. [file Data_Sheet_2.docx]

**Table S1 Search strategy of PubMed**

| **Number** | **Search terms** |
| --- | --- |
| #1 | China[MESH] |
| #2 | China[TW] OR Chinese[TW] |
| #3 | #1 OR #2 |
| #4 | Depressive Disorder[MESH] |
| #5 | Depressive Disorder, Major[MESH] |
| #6 | Depression[MESH] |
| #7 | depression[TIAB] OR depressions[TIAB] OR Depressive[TIAB] OR Depressed[TIAB] OR Melancholia[TIAB] OR Melancholias[TIAB] OR MDD[TIAB] OR MDE[TIAB] OR dysthymic disorder[TIAB] |
| #8 | #4 OR #5 OR #6 OR #7 |
| #9 | Diabetes Mellitus[MESH] |
| #10 | Diabetes Mellitus[TIAB] OR Diabetes[TIAB] OR Diabetes Insipidus[TIAB] OR Diabetic[TIAB] OR MODY[TIAB] OR glucose intolerance[TIAB] |
| #11 | # 9 OR #10 |
| #12 | #3 AND #8 AND #11 |

**Table S2 Search strategy of EMBASE**

| **Number** | **Search terms** |
| --- | --- |
| #1 | 'diabetes mellitus'/exp |
| #2 | 'diabetes mellitus':ab,ti OR diabetes:ab,ti OR 'diabetes insipidus':ab,ti OR diabetic:ab,ti OR mody:ab,ti OR 'glucose intolerance':ab,ti |
| #3 | #1 OR #2 |
| #4 | 'depression'/exp |
| #5 | depression:ab,ti OR 'major depression':ab,ti OR 'depression disorder':ab,ti OR depressions:ab,ti OR depressive:ab,ti OR depressed:ab,ti OR melancholia:ab,ti OR melancholias:ab,ti OR dysthymia:ab,ti OR 'dysthymic disorder':ab,ti OR mdd:ab,ti OR mde:ab,ti |
| #6 | #4 OR #5 |
| #7 | 'china'/exp |
| #8 | 'chinese'/exp |
| #9 | china:ab,ti OR chinese:ab,ti |
| #10 | #7 OR #8 OR #9 |
| #11 | #3 AND #6 AND #10 |

**Table S3 Search strategy of the Cochrane Library**

| **Number** | **Search terms** |
| --- | --- |
| #1 | MeSH descriptor: [Diabetes Mellitus] explode all trees |
| #2 | ("diabetes mellitus"):ti,ab,kw OR (diabetes):ti,ab,kw OR ("diabetes insipidus"):ti,ab,kw OR ("diabetic"):ti,ab,kw OR ("glucose intolerance"):ti,ab,kw OR (MODY):ti,ab,kw |
| #3 | #1 OR #2 |
| #4 | MeSH descriptor: [Depressive Disorder] explode all trees |
| #5 | MeSH descriptor: [Depressive Disorder, Major] explode all trees |
| #6 | MeSH descriptor: [Depression] explode all trees |
| #7 | (depression):ti,ab,kw OR (depressions):ti,ab,kw OR (depressive):ti,ab,kw OR (depressed):ti,ab,kw OR (melancholia):ti,ab,kw OR (melancholias):ti,ab,kw OR (dysthymic disorder):ti,ab,kw OR (dysthymia):ti,ab,kw OR (MDD):ti,ab,kw OR (MDE):ti,ab,kw |
| #8 | #4 OR #5 OR #6 OR #7 |
| #9 | MeSH descriptor: [China] explode all trees |
| #10 | (China):ti,ab,kw OR (Chinese):ti,ab,kw |
| #11 | #9 OR #10 |
| #12 | #3 AND #8 AND #11 |

**Table S4 Search strategy of the China National Knowledge Internet (CNKI)**

| **Number** | **Search terms** |
| --- | --- |
| #1 | (篇关摘: 抑郁) OR (篇关摘: 抑郁症) OR (篇关摘: 抑郁障碍) OR (篇关摘: 忧郁) OR (篇关摘: 恶劣心境) |
| #2 | (篇关摘: 糖尿病) OR (篇关摘: 消渴症) OR (篇关摘:尿崩症) |
| #3 | (主题: 流行病学) OR (主题: 患病率) OR (主题: 发病率) |
| #4 | #1 AND #2 AND #3 |

**Table S5 Search strategy of the Wanfang Database**

| **Number** | **Search strategy** |
| --- | --- |
| #1 | 主题:(流行病学)+主题:(患病率)+主题:(发病率) |
| #2 | 主题:(抑郁)+主题:(抑郁症)+主题:(抑郁障碍)+主题:(恶劣心境)+全部:(忧郁) |
| #3 | 主题:(糖尿病)+主题:(尿崩症)+主题:(消渴症) |
| #4 | #1 AND #2 AND #3 |

**Table S6 Search strategy of the Chinese Biomedical Literature Database (CBM)**

| **Number** | **Search terms** |
| --- | --- |
| #1 | "抑郁"[不加权:扩展] OR "抑郁症"[不加权:扩展] |
| #2 | "抑郁"[常用字段:智能] OR "抑郁症"[常用字段:智能] OR "抑郁障碍"[常用字段:智能] OR "恶劣心境"[常用字段:智能] OR "忧郁"[常用字段:智能] |
| #3 | #2 OR #1 |
| #4 | "糖尿病"[不加权:扩展] |
| #5 | "糖尿病"[常用字段:智能] OR "消渴症"[常用字段:智能] OR "尿崩症"[常用字段:智能] |
| #6 | #5 OR #4 |
| #7 | "流行病学研究"[不加权:扩展] OR "流行病学方法"[不加权:扩展] OR "流行病学"[不加权:扩展] |
| #8 | "流行病学"[常用字段:智能] OR "患病率"[常用字段:智能] OR "发病率"[常用字段:智能] |
| #9 | #8 OR #7 |
| #10 | #9 AND #6 AND #3 |

**Table S7 Search strategy of the Chinese Science and Technology Periodical Database (VIP)**

| **Number** | **Search terms** |
| --- | --- |
| #1 | U=(糖尿病 OR 消渴症 OR 尿崩症) |
| #2 | U=(抑郁 OR 抑郁症 OR 抑郁障碍 OR 忧郁 OR 恶劣心境) |
| #3 | U=(流行病学 OR 患病率 OR 发病率) |
| #4 | #1 AND #2 AND #3 |
