# Supplementary material for: A Systematic Review and Meta-Analysis of the Prevalence and Risk Factors of Depression in Type 2 Diabetes Patients in China
Source: Front Med (Lausanne). 2022 May 10;9:759499. doi: 10.3389/fmed.2022.759499 (PMC9127805; doi:10.3389/fmed.2022.759499)
Supplement: Supplementary File 3 — List of excluded records by screening full text. [file Data_Sheet_3.docx]

**List of excluded records (*n*=347)**

**Hospital-based population (*n* = 208)**

1. Bai JB, Pan LM. Depression and Quality of Life in Diabetic Patients. *Internal Medicine Journal* (2010) 5(3). Epub 20101130.

2. Chang ER. Investigation on Mental Health Status and Psychological Nursing of Diabetic Patients. *Journal of Qilu Nursing* (2005) 11(5). Epub 20051230.

3. Chen HG. Investigation and Analysis of Depression in Diabetic Patients. *Hainan Medical Journal* (2010) 21(16):93-4.

4. Chen QH, Lou BY, Li WG, Zhang GL. Investigation and Analysis of Anxiety and Depression in Type 2 Diabetes Patients. *Occupation and Health* (2005) 21(5). Epub 20050730.

5. Chen WJ, Chen JX, Peng DX, Liu CT. Investigation of Depression and Its Related Factors in Type 2 Diabetes Patients. *Journal of Nursing Science* (2005) (13):64-6.

6. Chen X, Tan ZQ. Investigation of Anxiety and Depression in Type 2 Diabetes Mellitus and Its Effect on Diabetes Treatment. *Journal of Clinical Research* (2010) 27(8):1454-5. doi: 10.3969/j.issn.1671-7171.2010.08.021.

7. Chen YY, Wu Y. Prevalence and Related Risk Factors of Depression in Patients with Type 2 Diabetic Peripheral Neuropathy. *Psychological Doctor* (2017) 23(21):163-4.

8. Chen ZX. The Incidence and Influencing Factors of Anxiety and Depression in Patients with Type 2 Diabetes and Pre-Diabetes [dissertation thesis]: Nanchang University;Medical College of Nanchang University (2016).

9. Cheng L, Wang DH, Zhao RR, Pang K. Gastrointestinal Symptoms and Its Influencing Factors in Patients with Type 2 Diabetes. *Journal of Nursing Science* (2016) 31(23). Epub 20170605.

10. Cheng N, Lu LS, Kang M, Song Q, Yang XM, Shi WY. Relative Factors of Diabetes Mellitus Patients Combined with Anxiety and Depression. *Chinese Journal of Difficult and Complicated Cases* (2011) 10(2). Epub 20111114.

11. Cheng TYL, Boey KW. Coping, Social Support, and Depressive Symptoms of Older Adults with Type Ii Diabetes Mellitus. *Clinical Gerontologist* (2000) 22(1):15-30. doi: 10.1300/J018v22n01_03.

12. Cong JY, Zhao Y, Xu QY, Zhong CD, Xing QL. Health-Related Quality of Life among Tianjin Chinese Patients with Type 2 Diabetes: A Cross-Sectional Survey. *Nurs Health Sci* (2012) 14(4):528-34. Epub 2012/09/11. doi: 10.1111/j.1442-2018.2012.00734.x.

13. Cun LJ. Psychological Problems and Nursing Countermeasures of 256 Patients with Type 2 Diabetes Mellitus. *Medical Innovation of China* (2010) 7(16). Epub 20100829.

14. Dong J. Clinical Analysis of Depression and Type 2 Diabetes *Chinese Journal of Urban and Rural Enterprise* (2013) 28(01):29-31.

15. Duan ZJ. Clinical Study of Type 2 Diabetes Patients with Depression and Related Factors [dissertation thesis]: Kunming Medical College (2009).

16. Duan ZJ. The Relationship between Fasting Blood Glucose Level and Depression in Newly Diagnosed Type 2 Diabetes Patients. *Modern Diagnosis and Treatment* (2010) 21(4).

17. Duan ZJ, Zhu X, Yang R. Clinical Study on Depressive Symptoms and Risk Factors in Type 2 Diabetic Patients. *Journal of Logistics University of PAP(Medical Sciences)* (2013) 22(03):174-7+81.

18. Fan HG. Depression and Related Factors in Patients with Type 2 Diabetes Mellitus. *Modern Practical Medicine* (2015) 27(5):606-8. doi: 10.3969/j.issn.1671-0800.2015.05.031.

19. Fan WY. Analysis of Type 2 Diabetes Mellitus Patients with Depression. *Chinese Nursing Research* (2005) 19(3).

20. Fung ACH, Tse G, Cheng HL, Lau ESH, Luk A, Ozaki R, et al. Depressive Symptoms, Co-Morbidities, and Glycemic Control in Hong Kong Chinese Elderly Patients with Type 2 Diabetes Mellitus. *Front Endocrinol (Lausanne)* (2018) 9:261. Epub 2018/06/14. doi: 10.3389/fendo.2018.00261.

21. Gao H, Niu YM, Gao QH. The Relationship between the Psychological Disturbance and Metabolic Control in the Adults with Type 2 Diabetes. *Journal of Heze Medical College* (2010) (1). Epub 20100715.

22. Gao H, Tan KL, Zhou Y, Duan MJ, Zhou C. Investigation on Depression-Causing Factors in Older Adults with Type 2 Diabetes. *Journal of Nursing Science* (2009) 24(6). Epub 20090815.

23. Gao HY, Xu N, Huang XX. Investigation and Analysis of Depression in Diabetic Patients. *Journal of Qiqihar Medical University* (2007) 28(8). Epub 20131012.

24. Gen FQ, Shi DJ, Wu CL, Wang GY. Survey and Care to the Common Psychological Concerns among Type 2 Diabetes. *Modern Nurse* (2013) (9):89-91.

25. Guo H, Chen YZ. Clinical Perspectives on Diabetes and Depression. *Chinese Journal of Practical Nervous Diseases* (2011) 14(11):69-70.

26. Guo YS, Du XX, Sun L, Mao Q, Liu LF. Investigation and Analysis of Mental Health Status of Type 2 Diabetes Patients. *Medical Journal of Chinese People's Health* (2009) 21(22). Epub 20100330.

27. Han Y, Wu Y, Li XN, Cui Q, Yang P, Shen MY. Correlation Evaluation between Quality of Life and Emotion in Patients with Type 2 Diabetes Mellitus. *Changzhou practical medicine* (2015) 31(4). Epub 20160204.

28. He CH, Sha DX, Zhong S. Mental Health State of Type 2 Diabetes. *Journal of Psychiatry* (2003) 16(1). Epub 20031231.

29. He M, Zhu HX, Cai Y, Yan Y. Survey of the Psychological Health Status of Home Caregivers for Elderly Diabetic Patients in Xujiahui Community in Shanghai. *Shanghai Medical & Pharmaceutical Journal* (2020) 41(10). Epub 20200908.

30. Hou NN, Sun JZ, Liu Y, Li XJ, Liu ZS, Sun XD. Assessment of Depression in Patients with T2dm. *Chinese Journal of Diabetes* (2013) 21(9):797-800. doi: 10.3969/j.issn.1006-6187.2013.09.009.

31. Hsu YL, Su DH, Kuo SC. Health Literacy and Depression in Women with Type 2 Diabetes Mellitus. *Clinics (Sao Paulo)* (2020) 75:e1436. Epub 2020/06/04. doi: 10.6061/clinics/2020/e1436.

32. Hu LZ, Li RH, Wang QL. The Onset and Relative Risk Factors Associated with the Depression and Type 2 Diabetes *Henan Journal of Preventive Medicine* (2012) 23(4):327-8.

33. Huang YH, Liu JY, He H, Zhang L. Prevalence of Depression and Type 2 Diabetes and Investigation of Risk Factors. *Sichuan Journal of Physiological Sciences* (2011) 33(04):170-2.

34. Ji L, Zhang Y, Zhang Q, Zheng H, Sun W, Zhu X, et al. Self-Reported Depressive Symptoms Might Be Associated with Sudomotor Dysfunction in Chinese T2dm Patients. *Exp Clin Endocrinol Diabetes* (2019). Epub 2019/11/15. doi: 10.1055/a-1025-3724.

35. Ji M, Ren D, Dunbar-Jacob J, Gary-Webb TL, Erlen JA. Self-Management Behaviors, Glycemic Control, and Metabolic Syndrome in Type 2 Diabetes. *Nurs Res* (2020) 69(2):E9-e17. Epub 2020/02/29. doi: 10.1097/nnr.0000000000000401.

36. Ji SL, Shi YL, Liu HW, Song L. Study on Influencing Factors of Quality of Life in 191 Patients with Type 2 Diabetes Mellitus. *Journal of Practical Diabetology* (2008) 4(5). Epub 20090130.

37. Jia Y, Wang JQ, Teng XY. Study on the Correlation between Psychological Health Level and Self-Management in Patients with Type 2 Diabetes Mellitus. *Shanghai Nursing* (2004) 4(4):1-3. doi: 10.3969/j.issn.1009-8399.2004.04.001.

38. Jiang SR, Yang JC, Li X. Investigation of Mental Health Status and Related Factors in Newly Diagnosed Type 2 Diabetic Patients. *Journal of Practical Diabetology* (2013) 9(3). Epub 20131226.

39. Junmei Y, Yeung R, Luk A, Wong R, Kong A, Ozaki R, et al. The Relationship between Negative Emotions and Quality of Life in High Risk Patients with Type 2 Diabetes in Hong Kong. *Diabetologia* (2014) 57(1):S429-S30. doi: 10.1007/s00125-014-3355-0.

40. Lee S, Chiu A, Tsang A, Chow CC, Chan WB. Treatment-Related Stresses and Anxiety-Depressive Symptoms among Chinese Outpatients with Type 2 Diabetes Mellitus in Hong Kong. *Diabetes Res Clin Pract* (2006) 74(3):282-8. Epub 2006/05/17. doi: 10.1016/j.diabres.2006.03.026.

41. Li BX, Zhou X, Liang XX, Chang WL, Zhao SX, Zhang YL. The Alternation of Bone Metabolism Index and Bone Mineral Density of Elderly Male Type 2 Diabetes Mellitus Combined with Depression Patients. *Chinese Journal of the Frontiers of Medical Science(Electronic Version)* (2015) 7(12):17-20. doi: 10.3969/j.issn.1674-7372.2015.12.005.

42. Li G, Tian WZ, Cao YL, Yong SM. Clinical Characteristics and Risk Factors of Type 2 Diabetes Mellitus with Depressive Disorder. *Ningxia Medical Journal* (2013) 35(09):848-9.

43. Li LM, Chen P. Analysis of Depression and Its Influencing Factors in Patients with Type 2 Diabetic Peripheral Neuropathy. *Modern Nurse* (2011) (02):115-7.

44. Li LN, Wang Y, Li FQ. Investigation of Depression and Treatment Compliance in Elderly Diabetic Patients. *Journal of Nurses Training* (2012) 27(24). Epub 20130520.

45. Li N, Ju ML, Huan HM, Chen MJ. Prevalence and Risk Factors of Comorbid Depression in Community Elderly Patients with Type 2 Diabetes Mellitus. *Journal of Neuroscience and Mental Health* (2017) 17(5):343-5,50. doi: 10.3969/j.issn.1009-6574.2017.05.010.

46. Li N, Ju ML, Huan HM, Chen MJ. Correlation between Depressive Symptoms and Diabetes Self-Management in Elderly Patients with Type 2 Diabetes in the Community. *Laboratory Medicine and Clinic* (2018) 15(7):979-81. doi: 10.3969/j.issn.1672-9455.2018.07.028.

47. Li RN, Zhao HY, Wang DD, Wang Y, Liu RT, Zhang QF, et al. Risk Factors for Comorbid Depression in Type 2 Diabetes. *Chinese Journal of Gerontology* (2018) 38(09):2055-7.

48. Li SL, Li Y. Study on the Relationship between Type 2 Diabetes Mellitus with Depression and Insulin Resistance. *Clinical Focus* (2009) 24(20):1799-800.

49. Li XJ, Hou NN, Song YP, Liu K, Sun JZ, Liu Y, et al. Relationship between Depression in Type 2 Diabetic Patients with Personality Characteristics and Social Support. *Journal of Modern Medicine & Health* (2012) 28(7). Epub 20120924.

50. Li XL, Chen J. Investigation on Life Satisfaction and Mental Health Status of Diabetes Patients in Community. *Hebei Journal of Traditional Chinese Medicine* (2009) 31(8). Epub 20091215.

51. Li YF, Hou RN, Cui CY, Zhang YJ, Liu YQ, Ma HW, et al. The Research on Relations among Self-Esteem, Anxiety and Depression of Patients with Type 2 Diabetes Mellitus. *Journal of Nursing Administration* (2017) 17(8). Epub 20180309.

52. Li YH, Liu S, Pan ZX, Li F, Chen YP. Analysis of Prevalence of Depression for Patients Newly Diagnosed with Type 2 Diabetes and Its Independent Risk Factors. *China Medical Herald* (2013) 10(05):53-5.

53. Li YL, Xu Q. Study on Self-Management Behavior of Type 2 Diabetes Patients with Depression. *Chinese General Practice Nursing* (2011) 9(11). Epub 20110820.

54. Li YZ, Li RX, Lin LK. Study on Depression of Elderly Patients with Chronic Diseases in Community Clinics in Shenzhen. *China Health Care & Nutrition* (2012) (z2):84-5.

55. Li ZZ, Qin GJ, Chen SS, Wan YM. Differences in Psychological Disorders between Type 1 and Type 2 Diabetes. *Medicine & Philosophy* (2009) 30(22):68,73.

56. Liao PS. A Clinical Study Ofthe Risk Factors in of Depression in Type 2 Diabetics Mellitus. *Journal of Kunming Medical University* (2011) 32(07):63-8.

57. Lin F. Clinical Investigation of Elderly Type 2 Diabetic Patients Complicated by Depression. *Journal of Modern Medicine & Health* (2012) 28(09):1432-3.

58. Lin K. Clinical Observation of Depression in Elderly Patients with Type 2 Diabetes Mellitus. *Chinese Journal of Gerontology* (2004) 24(7).

59. Liu AN, Li HP, Zhou LH, Zheng HY, Shen DY, Zeng Z. Analysis of Quality of Life of Patients with Type 2 Diabetes and Influencing Factors. *Journal of Nursing(China)* (2010) (3). Epub 20121127.

60. Liu AN, Zheng HY, Li HP, Wu WQ. The Research in Pat Ients with Type 2 Diabetes on the Impact of Psychological Factors. *Medicine & Philosophy* (2010) (6B). Epub 20101015.

61. Liu H, Bi Y, Zhu DL, Miu XP. Correlation Analysis of Depression and Treatments in Type 2 Diabetes Mellitus in Jiangsu Province. *Chinese Journal of Diabetes* (2014) 6(10):717-20.

62. Liu H, Gao J, Zhao BG, Zhang YW, Yang RH. The Relationship between Negative Mood and Attitude and Glucose Metabolism in Elderly Patients with Type 2 Diabetes. *Chinese Journal of Clinical Healthcare* (2013) 16(5). Epub 20140326.

63. Liu KX, He QJ, Li T. Analysis of Anxiety and Depression in Patients with Type 2 Diabetic Retinopathy at Different Stages. *Journal of Medical Information* (2014) (26). Epub 20200430.

64. Liu XN, Zhang YL. Forecast and Verification for Risk Factors of Type 2 Diabetes Patients with Depression. *Hebei Medicine* (2013) 19(2):232-4. doi: 10.3969/j.issn.1006-6233.2013.02.28.

65. Liu YJ, Song XF, Wang AH, Wang L, Song W, Xu ZR. Depression in Type 2 Diabetes. *Chinese Journal of Diabetes* (2004) (02):50-2.

66. Long HZ, Sun HF, Wu SX, Yang XH, Wu H, Wu YQ, et al. The Clinical Risk Factors Analysis of Depression in Type-2 Diabetes Mellitus. *World Chinese Medicine* (2015) 10(10):1607-10.

67. Long LL. Study on the Relationship between Depression and Quality of Life of Type 2 Daibetes Mellitus. *China Practical Medicine* (2010) (3).

68. Lu YY, Liu XY, Chen H, Feng BL, Wang CD, Lu K. Study on the Emotional Disorder and Constitution of Traditional Chinese Medicine in Typeⅱ Diabetic Patients for 323 Cases. *Chinese Medicine Modern Distance Education of China* (2016) (2). Epub 20160930.

69. Luo YT, Jin SY, Ma JL, Jiang KW, Yao ZF, Tao M. Interaction Analysis of Daily Life and Psychological Status of Patients with Type 2 Diabetes Mellitus. *Zhejiang Clinical Medical Journal* (2019) (6). Epub 20200315.

70. Lv ZJ. Investigation and Nursing of Anxiety and Depression in Diabetic Patients. *Journal of North China University of Science and Technology* (2007) 9(5):703-4. doi: 10.3969/j.issn.1008-6633.2007.05.085.

71. Ma LN, Yang N, Li Y, Huang YL. The Analysis on Characteristics of Depression Combined with Type2 Diabetes Mellitus. *Progress in Modern Biomedicine* (2012) 12(32). Epub 20130520.

72. Ma LX, Han WK. Investigation on Depression of Type 2 Diabetes Mellitus. *Chinese clinical new medicine* (2007) 007(005).

73. Ma YR. Analysis on 63 Cases Diabetes Complicated with Symptoms of Anxiety and Depression. *Chinese Nursing Research* (2001) 15(2):103-4. doi: 10.3969/j.issn.1009-6493.2001.02.034.

74. Mao QH, Xing DK. Anxiety and Depression of Patients with Type 2 Diabetes in a Certain Area and Its Correlation with Quality of Life. *Journal of Preventive Medicine of Chinese People's Liberation Army* (2017) 35(1). Epub 20170809.

75. Mao ZJ, Fu AD, Li YF, Jiang XF. Status Analysis and Nursing Countermeasures of Type 2 Diabetes Patients with Depression. *Chinese Journal of Modern Traditional and Western Medicine* (2005) 003(006).

76. Meng FJ, Suo XH, Zhang QG. Depression and Its Effect on Therapeutic Effect in Patients with Type 2 Diabetes. *Chinese Journal of Tissue Engineering Research* (2004) (06):1008-9.

77. Mou X, Zhou DY. The Related Factors of the Depression in the T2dm Patients. *Chinese Archives of Traditional Chinese Medicine* (2008) 26(6):1298-300. doi: 10.3969/j.issn.1673-7717.2008.06.072.

78. Ning B, Kang S, Quan DM, Cui YT, Chen HM, Liao XZ. Investigation of Depressive Symptoms in Type 2 Diabetes Mellitus. *Chinese Journal of Clinical Psychology* (1996) 4(3). Epub 19961231.

79. Peng CB, Xu YC, Zhu DJ, Hu Q, Li XF, Ke ZP. Elevated Serum Homocysteine Levels in Patients with Type 2 Diabetes Mellitus Complicated with Depression. *Chinese Journal of Diabetes* (2010) (5). Epub 20110303.

80. Peng L, Wang YT, Li J, Wang J. Evaluation of Depression and Related Risk Factors in Patients with Type 2 Diabetes Mellitus. *Chinese Journal of Misdiagnostics* (2012) 12(7):1614-5.

81. Peng YH, Shi XC, Chen X, Zheng HF. Emotional Disorder,Social Support and Life Quality of Type 2 Diabetes Mellitus Patients. *Chinese Rural Health Service Administration* (2015) (7). Epub 20160204.

82. Q. Y. Investigation and Analysis of Depression and Anxiety in Patients with Type 2 Diabetes Mellitus [dissertation thesis]: Liaoning University of Traditional Chinese Medicine (2010).

83. Qi XY, Cao YL. Related Analys Is of Depression in Type-2 Diabetes Mellitus. *Chronic Pathematology Journal* (2009) 11(05):103-4.

84. Qian F, Wang XH, Shan K, Lu JK. Analysis of Quality of Life of Elderly Patients with Type 2 Diabetes and Infl Uencing Factors. *Journal of Hunan University of Chinese Medicine* (2011) 31(12). Epub 20120427.

85. Qian YM, Wang BJ. Analysis of Risk Factors Associated with Depression in Type 2 Diabetes Mellitus. *Chinese Journal of Misdiagnostics* (2010) (1). Epub 20110130.

86. Qiu BH, Huang YM, Luo Y, Tang Y. A Survey of Depression and Related Factors in Type 2 Diabetes Patients in Community. *Chinese Community Doctors* (2020) 36(24):177-8. doi: 10.3969/j.issn.1007-614x.2020.24.087.

87. Rao YT, Zhang F, Lizha J, Liu JB. Investigate of the Mental Health of Patients with T2dm and High-Risk Groups in Urumqi Community. *Journal of Xinjiang Medical University* (2017) 40(5). Epub 20170921.

88. Ren LL, Feng ZZ. Study on the Influencing Factors of Depression in Type 2 Diabetes Patients. *Hebei Medicine* (2014) 36(2). Epub 20140701.

89. Ruan LM. Investigation of Depression in Diabetic Retinopathy. *China Journal of Chinese Ophthalmology* (2005) (01):45-6.

90. Shen HT, Song JP, Li J, Fu AJ, Shang SL, Liu Y, P., et al. Investigation and Analysis of Depressive Symptoms in Patients with Diabetic Peripheral Neuropathy. *Journal of Critical Care in Internal Medicine* (2015) (1). Epub 20151207.

91. Shen W. Correlation and Influencing Factors between Diabetes Mellitus and Depression. *Chinese Journal of Misdiagnostics* (2007) (23):5486-7.

92. Shen YX, Wu J, Zhao Y. Clinical Characteristics of Newly Diagnosed Young Patients with Type 2 Diabetes Mellitus. *Chinese Journal of Laboratory Diagnosis* (2019) (2). Epub 20200430.

93. Shi Y. Prevalence of Depression and Changes in Depression Scores after Glycemic Control in Patients with Type 2 Diabetes [dissertation thesis]: Xinjiang Medical University (2014).

94. Song HW. The Relationship between Anxiety and Depression and Glycosylated Hemoglobin in Diabetic Patients. *Henan Medical Research* (2018) 27(11). Epub 20190520.

95. Song X, Chen L, Zhang T, Xiang Y, Yang X, Qiu X, et al. Negative Emotions, Self-Care Activities on Glycemic Control in Adults with Type 2 Diabetes: A Cross-Sectional Study. *Psychol Health Med* (2020):1-10. Epub 2020/08/05. doi: 10.1080/13548506.2020.1799042.

96. Sun JZ, Hou NN, Wang YY, Liu Y, Li XJ. The Relationsh Ip between Type 2 Diabetic Pat Ients 'Quality of Life, Depression Symptom and Social Support. *China Journal of Health Psychology* (2012) 20(4). Epub 20120830.

97. Sun T. Prevalence and Risk Factors of Depression in Type 2 Diabetes Mellitus. *Chinese Primary Health Care* (2012) 26(03):124-5.

98. Sun XX, Shi FH, Ma J, Yang ML, Liu W, Wang LH, et al. Study on Glycemic Profiles and Emotional Scales in Diabetic Patients after the Outbreak of Covid-19. *Chinese Journal of Endocrinology and* (2020) 36(8). Epub 20201128.

99. Sun YH. Risk Factors of Depressive Mood Disorder in Patients with Type 2 Diabetes Mellitus [dissertation thesis]: Xinjiang Medical University (2010).

100. Sun YH, Ge JP, Chen XY. Factors Associated with Depressive Mood Disorder in Type 2 Diabetes. *Chinese Journal of Gerontology* (2013) 33(07):1614-7.

101. Tang W, Cai Y, Huang XP, Cui D, Jiang L, Liu C. Investigation of Depression and Anxiety in Middle-Aged and Elderly Patients with Type 2 Diabetes Mellitus. *Practical Geriatrics* (2009) 23(05):373-5.

102. Tang W, Cai Y, Huang XP, Duan Y, Zhang M, Cui D, et al. Survey of Depression Status in Patients with Type 2 Diabetes Mellitus and Analysis of Risk Factors. *Journal of Clinical Medicine in Practice* (2012) 16(05):125-8.

103. Tsai KW, Chiang JK, Lee CS. Undiagnosed Depression in Patients with Type 2 Diabetes and Its Associated Factors. *Tzu Chi Medical Journal* (2008) 20(1):44-8.

104. TUERDI ABLKM, YUSUPU ABB, AILI BHEGL. The Analysis of Depression in 2-Type Diabetes Mellitus. *Journal of Xinjiang Medical University* (2009) 32(08):1085-6+90.

105. Wan HP, Deng XR. Cognitive Impairment in Type 2 Diabetes Patients with Depression. *Chinese Journal of Practical Nervous Diseases* (2013) 16(04):7-9.

106. Wang AH, Hu YG, Wang JM. A Study on Depression and Anxiety in Patients with Diabetes Mellitus. *Shanghai Medical Journal* (2003) 26(8). Epub 20040430.

107. Wang AR, Tang TT, Zhou JY, Zou ZX, Wang MQ. A Study on the Correlation between Psychological Status and Quality of Life of Type 2 Diabetes Patients. *Sichuan Medical Journal* (2020) 41(4). Epub 20200908.

108. Wang C. Discussion on the Relationship between Senile Somatic Disorders and Depressive Psychological Disorders. *Chinese Journal of Practical Traditional and Western Medicine* (2004) 17(21). Epub 20041130.

109. Wang D, Shi L, Li L, Guo X, Li Y, Xu Y, et al. Subthreshold Depression among Diabetes Patients in Beijing: Cross-Sectional Associations among Sociodemographic, Clinical, and Behavior Factors. *J Affect Disord* (2018) 237:80-6. Epub 2018/05/25. doi: 10.1016/j.jad.2018.05.016.

110. Wang F, Sun YL, Dai JY, Zeng Q, Guo FF. Cross-Sectional Study and Risk Factors Ofdepression in Patients with Type 2 Diabetes Mellitus in Beijing. *Progress in Modern Biomedicine* (2018) 18(05):902-6.

111. Wang HL, Li YX, Kang YL. Psychological Investigation of 180 Cases of Diabetes *Medical Journal of Chinese People's Health* (2011) 23(2). Epub 20110525.

112. Wang HM, Zhang Y. Clinical Observation of Mental Disorders in 122 Patients with Type 2 Diabetes Mellitus. *Shandong Medical Journal* (2007) 47(10). Epub 20131012.

113. Wang J. Diabetes Mellitus Combined with Anxiety and Depression: An Analysis of 60 Cases. *Chinese medicine modern distance education of china [zhong guo zhong yi yao xian dai yuan cheng jiao yu]* (2010) 8(24). Epub 20110820.

114. Wang J, He M, Zhao X. Depressive Symptoms, Family Functioning and Quality of Life in Chinese Patients with Type 2 Diabetes. *Can J Diabetes* (2015) 39(6):507-12. Epub 2015/08/25. doi: 10.1016/j.jcjd.2015.06.001.

115. Wang J, Xiao LF, Fu ZR, Jiang YX, Ren P. Impact of Daily Periodicity Types on Glycemic Control and Depression for Patients with Type 2 Diabetes Mellitus. *Journal of Nursing Science* (2017) 32(21). Epub 20180428.

116. Wang P, Xin M, Miao N, Zhu J. A Study on the Relationship between Anxiety & Depression and Cognition & Coping Style in Patients with Type Ⅱ Diabetes. *Journal of Qilu Nursing* (2014) 20(7). Epub 20141027.

117. Wang XL, Liu HQ. Type 2 Diabetes Mellitus Complicated with Depression and Anxiety and Its Relationship. *Chinese Journal of Urban and Rural Enterprise* (2016) 31(09):118-9.

118. Wei J, Jiao K, Li XJ. Investigation and Analysis of the Types and Causes of Negative Emotions in Patients with Type 2 Diabetes Mellitus. *Practical Journal of Cardiac Cerebral Pneumal* (2016) 24(B04). Epub 20170323.

119. Wen XL, Yao GL. Survey on Depressive Disorder among Type 2 Diabetics. *China Journal of Health Psychology* (2012) 20(9):1319-20.

120. Weng XQ, Chen ZM, Yang B. Study of Depression and Anxiety in Patients with Type-2 Diabetes Mellitus. *Modern Preventive Medicine* (2012) 39(15). Epub 20121224.

121. Weng XQ, Chen ZM, Yang B. Association between Substance P Change and Depression and Anxiety in Patients with Type-2 Diabetes Mellitus. *Modern Preventive Medicine* (2013) 40(3). Epub 20130606.

122. Wu B. Investigation and Correlation Analysis of Dietary Quality and Depression in Patients with Type 2 Diabetes Mellitus [dissertation thesis]: Nanjing University of Traditional Chinese Medicine (2015).

123. Wu H, Pan CL, Lin TF, Xia XY, Chen YS. Correlation between Self-Management Behaviors and Family Members' Psychological Status in Patients with Type 2 Diabetes Mellitus. *Journal of Shantou University Medical College* (2017) 30(3). Epub 20180428.

124. Wu LY, Huang FL, Wang QP, Liu ZJ. Study on the Correlation between Depression after Type 2 Diabetes and Chinese Medicine Constitution. *JOURNAL OF NEW CHINESE MEDICINE* (2019) (6). Epub 20191110.

125. Wu QH. Clinical Observation on the Relationship between Type 2 Diabetes Complicated with Depression and Diabetic Nephropathy. *Zhejiang Clinical Medical Journal* (2008) 10(7):939. doi: 10.3969/j.issn.1008-7664.2008.07.060.

126. Wu WQ. Analysis of Quality of Life in Patients with Type 2 Diabetes Mellitus Complicated by Depression. *New Medicine* (2010) 20(6). Epub 20110820.

127. Wu YL. Serum Esrage Levels in Patients with Type 2 Diabetes Mellitus Complicated with Depression and Its Correlation with Tcm Liver-Qi Stagnation Syndrome [dissertation thesis]: Fujian University of Traditional Chinese Medicine (2011).

128. Wu ZX, Yue Q, editors. Investigation and Analysis of Depression and Anxiety in Patients with Type 2 Diabetes Mellitus. 2010.

129. Xia Y, Xu Y, Li J. Related Factors of Cognitive Impairment in Elderly Type 2 Diabetes Mellitus Patients *International Medicine and Health Guidance* (2016) 22(9):1258-61. doi: 10.3760/cma.j.issn.1007-1245.2016.09.025.

130. Xie YL, Su XQ, Zhang YW. Investigation and Analysis of Mood in Patients with Diabetic Peripheral Neuropathy. *Medical Information* (2018) 31(5). Epub 20181230.

131. Xie YL, Zhang YW, Su XQ. Investigation and Analysis the Emotion in 267 Patients with Type 2 Diabetes Mellitus. *Jiangxi Medical Journal* (2012) 47(12):1078-80.

132. Xu L, Ren J, Cheng M, Tang K, Dong M, Hou X, et al. Depressive Symptoms and Risk Factors in Chinese Persons with Type 2 Diabetes. *Arch Med Res* (2004) 35(4):301-7. Epub 2004/08/25. doi: 10.1016/j.arcmed.2004.04.006.

133. Xu L, Wang DQ, Ren JM, Cheng M, Tang KX, Dong M, et al. The Prevalence of Depression and Its Risk Factors in Type 2 Diabetics. *Chinese Journal of Diabetes* (2003) (01):49-53.

134. Xu L, Yang X. Investigation and Nursing of Anxiety and Depression in Elderly Patients with Type 2 Diabetes Mellitus. *West China Medical Journal* (2010) (12). Epub 20110710.

135. Xu W, Guan JX, Wu JL, Guo X, Hu Y, Zheng YX. Investigation of Treatment Compliance, Disease Development, and Depression and Anxiety in Patients with Type 2 Diabetes Mellitus in Community. *Hainan Medical Journal* (2019) 30(8). Epub 20191110.

136. Xu XM, Yang SY, Zhang Q, Zeng J. Randomized Controlled Study of the Relationship between T2dm and Anxiety and Depression. *Sichuan Mental Health* (2015) 28(6). Epub 20161207.

137. Xue RL, Duan WF, Shi YZ, Lou BY. Mood Disorders in Type 2 Diabetes Mellitus. *Chinese Journal of Tissue Engineering Research* (2002) 6(15). Epub 20021231.

138. Yan CM, Deng QN, Zhong WZ. Correlations among Metabolic Syndrome and Mild Cognitive Impairment. *Chinese Medical Journal* (2011) 91(45). Epub 20120518.

139. Yan SQ, Shen JF. Study on Risk Factors of Diabetes Depression. *Proceedings of the fifth National Conference on Neurology of Integrated Traditional Chinese and Western Medicine*; Chengdu, China(2004).

140. Yang HH. Correlation between Sleep Quality and Basic Physiological Indicators, Emotion and Cognition in Elderly Patients with Chronic Diseases [dissertation thesis]: Chengdu Medical College (2016).

141. Yang J, Li S, Zheng Y. Predictors of Depression in Chinese Community-Dwelling People with Type 2 Diabetes. *J Clin Nurs* (2009) 18(9):1295-304. Epub 2009/05/06. doi: 10.1111/j.1365-2702.2008.02703.x.

142. Yang J, Li SJ. Investigation and Analysis of Depression and Related Factors in Community Patients with Type 2 Diabetes Mellitus. *Chinese Journal of Nursing* (2009) (7). Epub 20091015.

143. Yang JH, Xiao XJ, Hua Y, Liu Y. A Survey of Prevalence of Constipation in Diabetic Patients in a Community of Shanghai. *Shanghai Medical & Pharmaceutical Journal* (2020) 41(22):54-7.

144. Yang K, Zhou W, Ye HY. A Study on the Correlation of Anxiety and Depression with Personality and Coping Style in Type 2 Diabetes Patients. *Sichuan Mental Health* (2008) 21(3). Epub 20081030.

145. Yang LP, Zhang XG, Gao J, Hu ZH, Zheng WJ, Yang J. Correlation between Diabetes Distress and Depressive Symptoms in Elderly Patients with Type 2 Diabetes in the Community. *Chinese Journal of Gerontology* (2017) 37(16):4099-100. doi: 10.3969/j.issn.1005-9202.2017.16.086.

146. Yang QQ, Shao D, Li J, Yang CL, Fan MH, Cao FL. Positive Association between Serum Levels of High-Sensitivity C-Reactive Protein and Depression/Anxiety in Female, but Not Male, Patients with Type 2 Diabetes Mellitus. *Biol Res Nurs* (2020) 22(2):178-87. Epub 2019/12/24. doi: 10.1177/1099800419894641.

147. Yang XH, Sun HF, Wu H, Wu SX, Wu YQ, Zhang ZJ, et al. Investigation on Tcm Syndromes of Type 2 Diabetes Mellitus Patients with Depression in Community. *Beijing Journal of Traditional Chinese Medicine* (2013) 32(09):687-9.

148. Yao L. Relationship between Depression and Hypoglycemic Events in Diabetic Patients. *Journal of Anhui Health Vocational & Technical College* (2010) (3). Epub 20101130.

149. Ye LX, Guo KQ, Liu XH, Du L, Wu G, Huang JH. The Effect of Anxiety and Depression Disorder on Cortisol Secretion in Newly Diagnosed Type 2 Diabetes Mellitus. *Chinese Journal of Diabetes* (2010) (4). Epub 20110303.

150. Ye SZ. Study on the Influencing Factors of Quality of Life in Patients with Type 2 Diabetes Mellitus. Zhejiang Journal of Traumatic Surgery (2014) 19(2). Epub 20141106.

151. Yi SW, Peng J. Investigation and Analysis of Anxiety, Depression and Self- Management in Patients with Type 2 Diabetes Mellitus. Journal of Zunyi Medical University (2018) 41(6). Epub 20190805.

152. Yu Z, Yang CH, Qiu LR, Xie XH, Cao LS. A Study of Self-Management of 168 Type 2 Diabetic Patients of Yi-People. *Practical Journal of Clinical Medicine* (2012) 9(5). Epub 20130131.

153. Zhang CX. The Relationship between Anxiety and Depression and Social Psychological Factors in Type 2 Diabetes Patients. *Chinese Mental Health Journal* (2006) 20(6).

154. Zhang CX, Chen YM, Chen WQ. Association of Psychosocial Factors with Anxiety and Depressive Symptoms in Chinese Patients with Type 2 Diabetes. *Diabetes Res Clin Pract* (2008) 79(3):523-30. Epub 2007/11/21. doi: 10.1016/j.diabres.2007.10.014.

155. Zhang CX, Tse LA, Ye XQ, Lin FY, Chen YM, Chen WQ. Moderating Effects of Coping Styles on Anxiety and Depressive Symptoms Caused by Psychological Stress in Chinese Patients with Type 2 Diabetes. *Diabet Med* (2009) 26(12):1282-8. Epub 2009/12/17. doi: 10.1111/j.1464-5491.2009.02840.x.

156. Zhang DY, Ma QH. Analysis of Risk Factors for Depression in Patients with Type 2 Diabetes Mellitus. *Journal of Practical Diabetology* (2018) 14(6). Epub 20190805.

157. Zhang HG, Wang Y, Wang XH. Investigation on the Status of Type 2 Diabetic Patients with Depression and Analysis on Its Related Risk Factors in Chaoyang Area. *Chinese Journal of New Clinical Medicine* (2010) 3(05):439-43.

158. Zhang J. Prevalence of Depression and Its Risk Factors in Type2 Diabetes Mellitus. *Chinese Journal of Endocrinology and Metabolism* (2011) 27(10).

159. Zhang J, Bi Y, Shen SM, Tong GY, Zhu DL, Cui SW, et al. Prevalence of Depression and Its Risk Factors in Type2 Diabetes Mellitus. *Chinese Journal of Endocrinology and Metabolism* (2011) 27(10). Epub 20120220.

160. Zhang JH. Prevalence of Depression and Its Risk Factors in Type2 Diabetes Mellitus. *For all Health* (2012) 6(15):1-3.

161. Zhang JH, Wu DH, Peng T. Research on Depression Syndrome and Other Related Factors among 101 Type-2 Diabetics Patients. *Chinese Journal of Behavioral Medicine and Brain Science* (2002) 11(4):384-5. doi: 10.3760/cma.j.issn.1674-6554.2002.04.013.

162. Zhang L, Li FH, Zhang W, Hou SC, Liu H, Ran RX. Depression and Related Factors in Newly Diagnosed Diabetic Patients. *Chinese Journal of Practical Nervous Diseases* (2016) 19(5). Epub 20161205.

163. Zhang L, Li XS, Li JR, Zhang JC, Qian HX. Investigation on Type 2 Diabetes Mellitus Complicated with Depression and Anxiety and Its Influence on Treatment and Prognosis. *Clinical Focus* (2012) 27(4). Epub 20120518.

164. Zhang MX. Analysis of Anxiety and Depression in Patients with Type 2 Diabetes Mellitus [dissertation thesis]: Soochow University (2013).

165. Zhang MX, Li WY, Wang WJ, Hu J. Analysis of Anxiety and Depression in Patients with Type 2 Diabetes Mellitus. *Journal of Hunan University of Chinese Medicine* (2016) 36(A01). Epub 20170323.

166. Zhang RX, Wang HL. Correlative Study on the Type 2 Diabetes with Disease Pain, Anxiety and Depression. *XINJIANG MEDICAL JOURNAL* (2015) (8). Epub 20161130.

167. Zhang W. The Preliminary Analysis of Depression Status and Risk Factors of Elder Type 2 Diabetes Patients *Chinese Nursing Management* (2010) (6). Epub 20100930.

168. Zhang W, Zheng W, Zhang L, Li FH. Investigation on Depression Status and Influencing Factors in Patients with Diabetic Complications. *Chinese Journal of Practical Nervous Diseases* (2016) 19(5). Epub 20161205.

169. Zhang WM. Chinese Medicine Dialectical Law Discussion of Diabetic Depression [dissertation thesis]: Guangzhou University of Chinese Medicine (2009).

170. Zhang WM, Wang SM, Liu YM, Wang YQ, Yun FY, Li SG. Clinical Study of Diabetes Mellitus Combined with Depression. *Journal of Guangzhou University of Traditional Chinese Medicine* (2013) 30(5). Epub 20140218.

171. Zhang XQ, Gao YL, Wei XZ. Survey on Health Locus of Control in Type 2 Diabetes Mellitus. *Modern Preventive Medicine* (2011) 38(19). Epub 20120220.

172. Zhang Y, Zhao TL, Sun D, Bai BH. Influencing Factors of Quality of Life in Patients with Type 2 Diabetes Mellitus. *Guide of China Medicine* (2014) 12(12). Epub 20141106.

173. Zhang YM, Zuo CF, Wang HJ, Wang YW, Song MQ. Study on the Relationship between Diabetes and Depression. *The Journal of Medical Theory and Practice* (2010) 23(09):1046-7+9.

174. Zhang YN, Sun LQ. Investigation and Analysis of Anxiety and Depression in Middle-Aged and Elderly Patients with Type 2 Diabetes Mellitus. *Shaanxi Medical Journal* (2013) 42(11):1546-7.

175. Zhang YR. Investigation on the Related Factors of Depression in Diabetic Patients. *Journal of Medical Forum* (2006) (10):62-3.

176. Zhao BL, Xu GF, Cheng W, Xu T, Chen SW, Zhang Y, et al. Related Study of Psychosocial and Biological Factors in Type 2 Diabetes Mellitus. *Journal of Clinical Psychiatry* (2006) 16(4). Epub 20061130.

177. Zhao X, Xie Y, Shen FF, Meng XH, Wang WL, Han LP, et al. Type 2 Diabetes Mellitus with Cognitive Dysfunction Related Factors Analysis. *Journal of Tianjin Medical University* (2009) 15(4). Epub 20100330.

178. Zhao Z. Investigation of Common Mental Disorders in Type 2 Diabetes Patients. *Journal of Clinical and Experimental Medicine* (2006) 5(5):512-4. doi: 10.3969/j.issn.1671-4695.2006.05.044.

179. Zhao Z, Wang SP. Prevalence of Anxiety and Depression and Its Risk Factors in Elderly Patients with Type 2 Diabetes. *Practical Geriatrics* (2006) (05):322-4.

180. Zhen YF, Zhai XG, Fang H, Liu XY, Xu G, Tian JL, et al. Influence of Depression on Glycemic Control in Patients with Type 2 Diabetes Mellitus. *Chongqing Medicine* (2018) 47(1). Epub 20180806.

181. Zheng L. Prevalence and Influencing Factors of Depression in Patients with Type 2 Diabetes Mellitus. *Journal of Preventive Medicine of Chinese People's Liberation Army* (2019) 37(10).

182. Zheng T, Ge B, Qin L, Chen B, Tian L, Gao Y, et al. Association of Plasma Dpp4 Activity and Brain-Derived Neurotrophic Factor with Moderate to Severe Depressive Symptoms in Patients with Type 2 Diabetes: Results from a Cross-Sectional Study. *Psychosomatic Medicine* (2020) 82(4):350-8. doi: 10.1097/PSY.0000000000000796.

183. Zhong HL, Lu XZ, Sun H, Pan XM, Bai JL. The Relationship between Hba1c and Depression in Patient with Type 2 Diabetes. *China Modern Medicine* (2014) 21(10):43-5.

184. Zhou H. The Clinical Study on Coexisting Major Depression in Patients with Type 2 Diabetes. *Sichuan Medical Journal* (2010) 31(04):486-7.

185. Zhou LH, Zhou XL, Shi XH, Ren FD. The Analysis of Depression in 2-Type Diabetes Mellitus. *Chinese Journal of Clinical Medicine* (2004) (06):1127-8.

186. Zhou XZ, Guo JP, Yan DE. Investigation and Analysis Ofmood and Sleep Quality in Newly Diagnosed Patients with Type 2 Diabetic Retinopathy. *Contemporary Medicine* (2019) 25(27). Epub 20200430.

187. Zhou ZY, Chen YH, Chen CR. Depression in Type 2 Diabetes Patients and Its Influencing Factors. *Occupational Health and Damage* (2004) 19(3):186. doi: 10.3969/j.issn.1006-172X.2004.03.011.

188. Zhu XH. Investigation of Depression in Patients with Type 2 Diabetes Mellitus. *Medical Innovation of China* (2010) 7(34):119-21.

189. Zhu YF. Investigation and Analysis of Anxiety and Depression and Its Influencing Factors among Elderly Patients with Type 2 Diabetes in the Community. *The Journal of Medical Theory and Practice* (2015) 28(18). Epub 20161130.

190. Zhuang Y, Ma QH, Pan CW, Lu J. Health-Related Quality of Life in Older Chinese Patients with Diabetes. *PLoS One* (2020) 15(2):e0229652. Epub 2020/02/28. doi: 10.1371/journal.pone.0229652.

191. Hu Z, Zhang HM. Analysis of Self-Perceived Aging Status and Its Influencing Factors in Elderly Diabetic Patients. *Journal of Henan University(Medical Science)* (2021) 40(5). Epub 20220221.

192. Cheng ZN, Xing QL, Xu HM, Bao D, Mu C. Analysis on Status and Influential Factors of Frailty in Elderly Patients with Diabetes Mellitus. *Tianjin Journal of Nursing* (2020) 28(3). Epub 20210311.

193. Long E, Feng S, Zhou L, Chen J, Shi L, Jiang X, et al. Assessment of Health-Related Quality of Life Using Euroqol-5 Dimension in Populations with Prediabetes, Diabetes, and Normal Glycemic Levels in Southwest China. *Frontiers in public health* (2021) 9:690111. doi: 10.3389/fpubh.2021.690111.

194. Zhang B, Wang Q, Zhang X, Jiang L, Li L, Liu B. Association between Self-Care Agency and Depression and Anxiety in Patients with Diabetic Retinopathy. *BMC ophthalmology* (2021) 21(1):123. doi: 10.1186/s12886-021-01883-w.

195. Sun XX, Shi FH, Ma J, Yang ML, Liu W, Wang LL, et al. Blood Glucose and Emotion Management in Patients with Diabetes after Covid-19. *Chinese Journal of Endocrinology and Metabolism* (2020) 36(8). Epub 20210603.

196. Xu W, Hu X, Zhang X, Ling C, Wang C, Gao L. Cognitive Impairment and Related Factors among Middle-Aged and Elderly Patients with Type 2 Diabetes from a Bio-Psycho-Social Perspective. *Diabetes, Metabolic Syndrome and Obesity: Targets and Therapy* (2021) 14:4361-9. doi: 10.2147/DMSO.S333373.

197. Zuo MF, Chen YC, Yu QM, Ma X, Wang Y. Correlation between Family Functioning with Metabolic Indicators in Diabetic Patients. *Journal of Nongken Medicine* (2021) 43(1). Epub 20211116.

198. Liu P, Song YW, Wang P, Tian GW, Zheng FJ, Lv L, et al. Correlation between Vitamin D Deficiency and Depression in Patients with Type 2 Diabetes Mellitus. *Journal of Shandong University(Health Sciences)* (2021) 59(6):51-6,102. doi: 10.6040/j.issn.1671-7554.0.2021.0296.

199. Wu R. Effect of Depression on Quality of Life and Self-Management in Patients with Diabetic Nephropathy Patients. *World Latest Medicine Information* (2020) (92). Epub 20210629.

200. Wang JT, Wang ZJ, Wu YQ, Wu JH, Wu Y, Wang XW, et al. Epidemiological Status and Characteristics of Common Comorbidities of Type 2 Diabetes Mellitus among 1.15 Million Patients in Beijing Area. *Journal of Third Military Medical University* (2021) 43(12):1126-32. doi: 10.16016/j.1000-5404.202101017.

201. Liu LL, Li HY, PC ZG, Lei YY, Zhang Y, Yu DH, et al. Factors Related to Visual Impairment in Type 2 Diabetes Patients in Xining City. *South China Journal of Preventive Medicine* (2021) 47(7):885-8. doi: 10.12183/j.scjpm.2021.0885.

202. Zhuang Y, Ma QH, Pan CW, Lu J. Health-Related Quality of Life in Older Chinese Patients with Diabetes. *PLoS ONE* (2020) 15(2). doi: 10.1371/journal.pone.0229652.

203. Song X, Chen L, Zhang T, Xiang Y, Yang X, Qiu X, et al. Negative Emotions, Self-Care Activities on Glycemic Control in Adults with Type 2 Diabetes: A Cross-Sectional Study. *Psychol Health Med* (2021) 26(4):499-508. Epub 2020/08/05. doi: 10.1080/13548506.2020.1799042.

204. Li X, Xu ZR, Wang AH, Niu WF, Li LJ, Mou YM, et al. Quality of Life and Affecting Factors in Elderly Patients with Type 2 Diabetes in Chinese Tertiary Hospitals. *Chinese Journal of Multiple Organ Diseases in the Elderly* (2020) 19(11). Epub 20210603.

205. Li J, Qiu X, Yang X, Zhou J, Zhu X, Zhao E, et al. Relationship between Illness Perception and Depressive Symptoms among Type 2 Diabetes Mellitus Patients in China: A Mediating Role of Coping Style. *Journal of Diabetes Research* (2020) 2020. doi: 10.1155/2020/3142495.

206. Wang L, Li J, Dang Y, Ma H, Niu Y. Relationship between Social Capital and Depressive Symptoms among Type 2 Diabetes Mellitus Patients in Northwest China: A Mediating Role of Sleep Quality. *Frontiers in Psychiatry* (2021) 12. doi: 10.3389/fpsyt.2021.725197.

207. Lin CL, Yu NC, Wu HC, Liu YC. Risk Factors Associated with Frailty in Older Adults with Type 2 Diabetes: A Cross-Sectional Study. *Journal of clinical nursing* (2021). doi: 10.1111/jocn.15953.

208. Wang Q. Study on Related Influencing Factors of Sleep Disturbance in Diabetic Peripheral Neuropathy. *Zhejiang Journal of Integrated Traditional Chinese and Western Medicine* (2022) 32(1).

**Ineligible publication type (abstract, editorial, letter) (*n* = 8)**

1. Xue YZ. Investigation and Analysis of Psychological Status of Type 2 Diabetes Patients in Shanxi Province. *Abstracts of the 11th National Conference on Psychology*; Kaifeng, Henan Province, China(2007).

2. Chan J. Physical and Psychological Health in Type 2 Diabetes. *Asia-Pacific Psychiatry* (2012) 4:36. doi: 10.1111/appy.12002.

3. Huang ZT, Cao GY, Yao SS, Chen ZS, Hu YH, Xu B. Cardiometabolic Multimorbidity and Depression in Adults Aged 45 Years and Older in China: Longitudinal Findings from the China Health and Retirement Longitudinal Study. *The Lancet* (2019) 394:S89. doi: 10.1016/S0140-6736(19)32425-0.

4. Jia M, Xie B, Ye XL, Tu YC, Yang N, Sun ZL. Multiple Analysis of Risk Factors for Depression in Chinese Type 2 Diabetic Patients. *Diabetologia* (2013) 56:S461. doi: 10.1007/s00125-013-3012-z.

5. Ning F, Wang YM, Wang SJ, Nan HR, Sun JP, Zhang D, et al. Bidirectional Associations between Depression and Type 2 Diabetes in Adults: Result from Qingdao Diabetes Prevention Program. *Diabetes Research and Clinical Practice* (2014) 106:S2.

6. Wang L, Gu A, Sun C, Xu H, Ni X, Wang R, et al. Cross-Sectional Study of Factors Correlated to Quality of Life in Patients with Coronary Artery Disease and Diabetic Retinopathy. *Journal of Biological Regulators and Homeostatic Agents* (2020) 34(3):1235-40.

7. Xie B, Yao L, Ye XL, Ju CP, Fang Y, Yang YXX, et al. Psychological Status and Diabetes-Related Distress of Type 2 Diabetes in Rural Communities of Nanjing, China. *Diabetes Research and Clinical Practice* (2014) 106:S253.

8. Three Diseases-Diabetes, Headache Associated with Drug Overuse and Depression - Are the Most Significant Negative Factors in Global Health-Adjusted Life Expectancy Changes. *Chinese Journal of Health Management* (2020) 14(1). Epub 20200804.

**Not cross-sectional studies (*n*=27)**

1. Hu C, Lin L, Zhu Y, Zhang Y, Wang S, Zhang J, et al. Association between Age at Diagnosis of Type 2 Diabetes and Cardiovascular Diseases: A Nationwide, Population-Based, Cohort Study. *Frontiers in Endocrinology* (2021) 12. doi: 10.3389/fendo.2021.717069.

2. Li H, Zheng D, Li Z, Wu Z, Feng W, Cao X, et al. Association of Depressive Symptoms with Incident Cardiovascular Diseases in Middle-Aged and Older Chinese Adults. *JAMA Network Open* (2019) 2(12). doi: 10.1001/jamanetworkopen.2019.16591.

3. Ye B, Xie R, Mishra SR, Dai X, Chen H, Chen X, et al. Bidirectional Association between Physical Multimorbidity and Subclinical Depression in Chinese Older Adults: Findings from a Prospective Cohort Study. *Journal of Affective Disorders* (2022) 296:169-74. doi: 10.1016/j.jad.2021.09.067.

4. Guan S, Fang X, Gu X, Zhang Z, Tang Z, Wu X, et al. The Link of Depression, Untreated Hypertension, and Diabetes with Mortality in Postmenopausal Women: A Cohort Study. *Clinical and Experimental Hypertension* (2021) 43(1):1-6. doi: 10.1080/10641963.2020.1790584.

5. Ning H, Du Y, Zhao Y, Liu Q, Li X, Zhang H, et al. Longitudinal Impact of Metabolic Syndrome and Depressive Symptoms on Subsequent Functional Disability among Middle-Aged and Older Adults in China. *Journal of Affective Disorders* (2022) 296:216-23. doi: 10.1016/j.jad.2021.09.061.

6. Bi YH, Pei JJ, Hao C, Yao W, Wang HX. The Relationship between Chronic Diseases and Depression in Middle-Aged and Older Adults: A 4-Year Follow-up Study from the China Health and Retirement Longitudinal Study. *Journal of Affective Disorders* (2021) 289:160-6. doi: 10.1016/j.jad.2021.04.032.

7. Han L, Shen S, Wu Y, Zhong C, Zheng X. Trajectories of Depressive Symptoms and Risk of Cardiovascular Disease: Evidence from the China Health and Retirement Longitudinal Study. *Journal of Psychiatric Research* (2022) 145:137-43. doi: 10.1016/j.jpsychires.2021.12.017.

8. A T, Meng L. Diabetes and Depression. *Journal of Practical Diabetology* (2011) 7(05):3-4.

9. Chen G, Liu J, Yang S, Lin L. Clinical Study of Type 2 Diabetes Mellitus with Depression. *Proceedings of the ninth National Conference of Endocrinology, Chinese Medical Association*; Dalian(2010). p. 496-.

10. He Q, Zhang W, Liu Y, Zhang XC, Ying Y, Sun J, et al. A Comparative Study of Depression Rate and Quality of Life between Type 2 Diabetes and Non-Type 2 Diabetes Elderly in a Community of Beijing. *Chinese General Practice Nursing* (2016) 14(22). Epub 20170323.

11. Huang M, Guo Z, Zhang J, Duan W, Zhang H. Investigation on the Prevalence of Depression in Type 2 Diabetes Mellitus in Senior Cadres. *Hebei Medical Journal* (2014) 36(22):3444-5.

12. Lan Y. Nursing Progress in Type 2 Diabetes Patients Complicated with Depression. *Psychology* (2018) (9):272-3.

13. Li N, Jiang S, F., Gao X, Ji JL, Hua X, Zhao NQ. Analysis of Anxiety and Depression in Type 2 Diabetes Patients. *Fudan University Journal of Medical Sciences* (2004) 31(6). Epub 20050330.

14. Luo L, Huo YH, Tuo XP. Association of Serum Cystatin C and Risk of Mild Cognitive Impairment in Elderly Patients with Type 2 Diabetes Mellitus. *Academic Journal of Second Military Medical University* (2011) 32(10). Epub 20120220.

15. Tang Y. Discussion and Treatment of Type 2 Diabetes Complicated with Depression. *Chinese Journal of Clinical Rational Drug Use* (2010) 3(03):116-7.

16. Wang LH, Wu ZD, Ying H. Analysis of Clinical Characteristics of Type 2 Diabetes Mellitus Complicated with Depression. *Health Vocational Education* (2013) 31(13):146-7.

17. Xiang H, Xu HS, H K, editors. Investigation of Depression in Type 2 Diabetes Patients and Analysis of Related Factors. *The 4th Pan-Asia Pacific Symposium on Mental Health*; 2005; ShangHai, China.

18. Xiang H, Xu HS, Yang SC. Investigation of Depression State for Patients with Type Ⅱ Diabetes. *Journal of Clinical Psychosomatic Diseases* (2006) 12(3). Epub 20060830.

19. Zang XY, Wang KF, Du WJ, Zhang XJ. Investigation of Quality of Life , Emotion, Social Support of Community Patients with Type 2 Diabetes. *Nursing Journal of Chinese People's Liberation Army* (2007) 24(4). Epub 20070730.

20. Zhang YL. Recognition of Depression in Patients with Type 2 Diabetes. *Chinese Journal of Postgraduates of Medicine* (2007) (16):73-4.

21. Zheng YR, Wei BJ, Xue YZ. Influencing Factors of Quality of Life in Elderly Patients with Type 2 Diabetes. *Chinese Journal of Gerontology* (2016) 36(8). Epub 20161130.

22. Zhou Y. Research Progress on the Interaction between Depression and Osteoporosis in Type 2 Diabetes Patients. *Shaanxi Medical Journal* (2013) (3).

23. Fisher EB, Qian Y, Tang PY, Liu Y, Coufal MM, Jia W. Diabetes Distress and Depression Are Reduced through Neighborhood Support in Peer Support Program in Shanghai. *Diabetes* (2020) 69. doi: 10.2337/db20-736-P.

24. Huang C-J, Hsieh H-M, Tu H-P, Jiang H-J, Wang P-W, Lin C-H. Major Depressive Disorder in Patients with Type 2 Diabetes Mellitus: Prevalence and Clinical Characteristics. *Journal of Affective Disorders* (2018) 227:141-8. doi: 10.1016/j.jad.2017.09.044.

25. Ken-Opurum J, Li L, Liu S, Chen Y, Grillo V, Stankus A. Pdb27 Association between Health Characteristics and Mode of Insulin Administration among Patients with Type Ii Diabetes in 2017 China National Health and Wellness Survey. *Value in Health Regional Issues* (2020) 22:S37. doi: 10.1016/j.vhri.2020.07.186.

26. Yan Z, Cai C, Song H, Jiang H, Sun B, Bai B, et al. Association of Diabetes and Prediabetes with Cognitive Impairment and Depression among Chinese Elderly People: The Confucius Hometown Aging Project. *Alzheimer's and Dementia* (2012) 8(4):P313. doi: 10.1016/j.jalz.2012.05.861.

27. Wang JH. People with Diabetes Have a High Risk of Depression. *Doctors online* (2020) 10(20).

**Wrong instruments for identifying depression (*n*= 5)**

1. Rao YT. A comparative study of behavior and psychology between type 2 diabetes patients, high-risk and health population in the community [dissertation thesis]: Xinjiang Medical University; 2017.

2. Yu GF, Luo Q, Wang L, et al. The Current Status of Psychosomatic Health in Elderly Patients With Type Ⅱ Diabetes Mellitus in Suining Community. Journal of Preventive Medicine Information 2019;35(8):886-9.

3. Wong EL, Xu RH, Cheung AW. Measurement of health-related quality of life in patients with diabetes mellitus using EQ-5D-5L in Hong Kong, China. Quality of Life Research. 2020;29(7):1913-21.

4. Che XY. Health-related quality of life of diabetes patients and related factors before and after healthcare reform in Gansu province. Chinese Journal of Epidemiology. 2021;42(9).

5. Tian S, Wang R, Qian M, et al. The association between diabetes mellitus and HRQoL of older people in Shanghai. BMC geriatrics. 2021;21(1):626.

**Unclear or ineligible type of diabetes (*n* = 17)**

1. Chen L. Analysis of anxiety and depression status of residents in a community in Beijing. Gems of Health. 2021(19):278-9.

2. Wang XY, Xu M, Li LL, et al. Relationship between glucose metabolism and depression in community residents over 40 years old in Guangzhou. Lingnan Journal of Emergency Medicine. 2021;26(5):536-8.

3. Yang JH, Xiao XJ, Liu Y, et al. A survey of prevalence of constipation in diabetic patients in a community of Shanghai. Shanghai Medical & Pharmaceutical Journal. 2020;41(22):54-7.

4. Zhang S, Chen Y, Jiang ZL, et al. Status and influencing factors of cognitive weakness in elderly patients with diabetes in community. Chinese Nursing Management. 2020;20(3).

5. Cui N, Cui J, Xu X, et al. Health Conditions, Lifestyle Factors and Depression in Adults in Qingdao, China: A Cross-Sectional Study. Frontiers in Psychiatry. 2021;12.

6. Zhang Y, Zhang S, Pan L, et al. Painful Diabetic Peripheral Neuropathy Study of Chinese Outpatients (PDNSCOPE): A Multicentre Cross-Sectional Registry Study of Clinical Characteristics and Treatment in Mainland China. Pain and Therapy. 2021;10(2):1355-73.

7. Deng QD, Hu YJ, Li LJ, et al. Mediating effect of depressive symptoms on diabetes-related distress and frailty in elderly patients with diabetes in the community. Medical Science Journal of Central South China 2020;48(3).

8. Lin ST, He F, Zhai YJ, et al. Analysis of rural residents' depression and its influencing factors. Journal of Preventive Medicine 2017;29(9).

9. Ma JL. Analysis on the disease status and clinical characteristics of elderly patients with diabetes complicated with hypertension in community. Chinese Baby. 2015(24):31-.

10. Sun MH. Screening of Depression and Anxiety in Elderly Patients with Chronic Diseases in Urban and Rural Communities Health Education and Health Promotion. 2018;13(5).

11. Tu QY, Han XH, Qin Y, et al. Prevalence of depression in adult residents and its influence factors in Jiangsu province. Modern Medical Journal. 2018(11).

12. Zhang AP. Regional distribution of psychological characteristics in diabetic patients. Proceeding of Clinical Medicine. 2011;20(8).

13. Chau PH, Woo J, Lee CH, et al. Older people with diabetes have higher risk of depression, cognitive and functional impairments: implications for diabetes services. J Nutr Health Aging. 2011;15(9):751-5.

14. Leung J, Fan VS, Mahadevan R. How do different chronic condition comorbidities affect changes in depressive symptoms of middle aged and older adults? Journal of Affective Disorders. 2020;272:46-9.

15. Yan S, Hong X, Yu H, et al. Prevalence of Diabetes and Health-Related Quality of Life Among Rural-to-Urban Nong Zhuan Fei Migrants in an Urban Area of Northern China, 2013. Public Health Rep. 2016;131(1):167-76.

16. He P, Hu Y, Li C, et al. Predictors of Depressive Symptoms Among Mid-Aged and Older Men With Diabetes in China. Res Theory Nurs Pract. 2019;33(1):6-22.

17. Zhuang Q, Wu L, Ting W, et al. Negative emotions in community-dwelling adults with prediabetes and hypertension. Journal of International Medical Research. 2020;48(4).

**No data on the numbers of patients with depression (*n* = 58)**

1. Chen H, Ji JL, Yang MR, Liu HL. Comparison of Physical and Mental Health Status among Different Communities in Kaohsiung City, Taiwan Province. *Shanghai Archives of Psychiatry* (2008) 20(6). Epub 20090315.

2. Chen PP, Tang S, Lou PA, Zhang P, Qiao C, Li T, et al. Analysis of Life Quality and Influencing Factors in Hypertension Patients, Diabetes Patients and Hypertension Combined with Diabetes Patients. *Chinese Journal of Prevention and Control of Chronic Diseases* (2016) (4). Epub 20161130.

3. Chen ZK, Liang YM, Zhou GP, Lv J, Zhu XQ, Liang YF. Risk Factors of Menopausal Women for Type 2 Diabetes. *Guangxi Medical Journal* (2005) 27(8):1193-4. doi: 10.3969/j.issn.0253-4304.2005.08.041.

4. Dong SH, Sun XR, Wang T, Xiao SF. Investigation and Analysis of Depressive Symptoms in Elderly of Community. *Journal of Internal Medicine Concepts & Practice* (2018) 13(3):170-4. doi: 10.16138/j.1673-6087.2018.03.008.

5. Dou MB, Mou ML, Han Y, Qin B. Explore the Risk Factors for Type 2 Diabete. *Preventive Medicine Tribune* (2005) 11(5). Epub 20060430.

6. Gong XH, Wang H, Shao XD. Investigation and Analysis of Aged Body Disease and Depression. *Modern Hospitals* (2013) 13(10):77-9. doi: 10.3969/j.issn.1671-332X.2013.10.036.

7. Gu YX, Hao XJ, Chen CX, Li SX, Li JM, Li YJ. Prevalence and Influential Factors of Chronic Diseases among Commumity Elderly People in Hebei Province. *Chinese Journal of Public Health* (2015) 31(2):132-6. doi: 10.11847/zgggws2015-31-02-02.

8. He LY, Zhang CJ, Yu X, Xu RF, Xu HY. Analysis of Non-Communicable Disease and Utilization of Health Resources on Elders in Qingpu District of Shanghai. *Chinese Journal of Public Health Engineering* (2013) 12(2):91-4.

9. Hou LS, Deng CY, Peng XC, Zhao YL, Chen L, Yue JR. Analysis of Risk Factors of Frailty in Patients with Type 2 Diabetes Mellitus. *Practical Geriatrics* (2020) 34(4):323-6. doi: 10.3969/j.issn.1003-9198.2020.04.005.

10. Jia LN, Liu FF, Zhuang HL, Wang XY, Lin YJ, Ding YL. Survey on Health Status and Community Health Service Demand of the Aged in Fuzhou City. *Chinese Journal of Geriatrics* (2011) 30(5):431-3. doi: 10.3760/cma.j.issn.0254-9026.2011.05.024.

11. Jia Y, Mei YY, Sheng CQ, Pan YJ, Cheng ZX, Cheng FY. Investigation and Correlation Analysis of Multimorbidity of Chronic Diseases among Residents Aged 55 Years and Over. *Chinese General Practice* (2016) 19(6). Epub 20160930.

12. Li Y, Liu JF, He SJ, Yan H, Li SY, YU LP. The Prevalence of Diabetes among Rural Residents Aged 15 and above in Hubei Province. *Chinese Journal of Disease Control & Prevention* (2017) 21(11):1088-91. doi: 10.16462/j.cnki.zhjbkz.2017.11.003.

13. Li Y, Li YF, Guo LF, Ma L, Tian QF. Common Chronic Comorbidity in the Elderly, Henan. *Modern Preventive Medicine* (2020) 47(15):2797-800.

14. Lu XM. Quality of Life and Its Influencing Factors in Patients with Type 2 Diabetes over 10 Years of Disease Course. *Chinese Journal of Practical Nursing* (2009) (11C). Epub 20100430.

15. Luo L, Huo YH, Tuo XP. Association of Serum Cystatin C and Risk of Mild Cognitive Impairment in Elderly Patients with Type 2 Diabetes Mellitus. *Academic Journal of Second Military Medical University* (2011) 32(10). Epub 20120220.

16. Luo MY. Regression Tree Analysis of Influencing Factors of Anxiety and Depression among the Elderly in Some Urban Communities of Jinan City [dissertation thesis]: Shandong University (2016).

17. Qiu YH, Guan Q, Wang J, Li X, Tan YW, Luo YJ. Cognitive Characteristics and Influencing Factors of Mild Cognitive Impairment in Community Elderly. *Chinese Journal of Clinical Psychology* (2018) 26(2):313-7,21. doi: 10.16128/j.cnki.1005-3611.2018.02.022.

18. Rao YT, JANGABIEKR LZ, Zhang F, Liu JB. A Comparative Analysis of Life Behavioral and Psychological Factors among T2dm Patients, High Risk Residents and Healthy Residents. *Chinese Journal of Prevention and Control of Chronic Diseases* (2017) 25(3). Epub 20171024.

19. Shen ZX, Zheng B, Yin G, YING DP, Wang J, Shen C. Comprehensive Assessment on the Health Status of Patients with Cardio-Cerebrovascular Diseases in Elderly Physical Examination Population. *Practical Preventive Medicine* (2018) 25(8):905-10. doi: 10.3969/j.issn.1006-3110.2018.08.003.

20. Song XF. Status of Quality of Life and Psychological and Social Support in Elderly Patients with Diabetes. *Chinese Journal of Gerontology* (2012) 32(18). Epub 20130131.

21. Wang RJ. Community Prevalence and Clinical Characteristics of Elderly Patients with Diabetes Mellitus and Hypertension. *Chinese Journal of Traditional Medical Science and Technology* (2014) (z2):119-20.

22. Wang YJ, Zhang SY, Zhao ZQ. A Study on the Influencing Factors of Anxiety among Newly Diagnosed Type 2 Diabetes Patients in Community Outpatient Department. *Chinese Primary Health Care* (2016) 30(10). Epub 20170323.

23. Wu YM. Study on Quality of Life and Influencing Factors of the Elderly in a Community in Shanghai [dissertation thesis]: Fudan University (2008).

24. Wu YM, Liu CH. Investigation and Analysis of Influencing Factors on Quality of Life of the Elderly in Shimen Community, Shanghai. *Chinese Journal of General Practitioners* (2009) 8(1):48-51. doi: 10.3760/cma.j.issn.1671-7368.2009.01.019.

25. Xiong Y, Miao RJ, Wang QQ, Zhou LJ, Gao L, Ma F. Prevalence and Influencing Factors of Mci among Community Elderly in Tianjin City. *Chinese Journal of Public Health* (2013) 29(1):1-4.

26. Xue YZ, Lv L, Hou YY, Wang WP, Zhao SF, Jing Y, et al. Regional Distribution of Mood Disorders and Social Support in Diabetic Patients. *Chinese Journal of Behavioral Medicine and Brain Science* (2008) 17(6). Epub 20090130.

27. Yang ZD, Chen DH, Qu ZW, Jiang Q, Zhang M, Sun XR, et al. Relationship of Depressive Disorders and Cognitive Function Impairment for the Elderly. *Medical Journal of Chinese People's Health* (2013) 25(15). Epub 20131226.

28. Yin JY, Zhou HS. Investigation and Intervention of Mental Health Status of Diabetic Patients. *Modern Nurse* (2014) (5). Epub 20150131.

29. Yuan J, Sun XR, Qu ZW, Zhang J, Jiang Q, Qin HY. Three-Year Follow-up Survey on Ederly Depressive Disorder in Shanghai Community. *Journal of Clinical Psychiatry* (2017) 27(01):25-7.

30. Zhang HD. Investigation and Analysis of Chronic Disease-Related Depression among Uygur Elderly in Zawar Township, Hotan Prefecture, Xinjiang [dissertation thesis]: Xinjiang Medical University (2011).

31. Zhang HD, ABULAITI PLD, MAIMAITIYIMING GLZE. Correlation between Depression and Related Risk Factors in the Elderly of Xinjiang. *Chinese Journal of Cardiovascular Rehabilitation Medicine* (2010) 19(1):7-10. doi: 10.3969/j.issn.1008-0074.2010.01.03.

32. Zhang P, Lou PA, Lou HQ, Zhao J, Chen PP, Qiao C, et al. Relationship between Anxiety and Glycosylated Hemoglobin in Type 2 Diabetes Mellitus. *Chinese Journal of Diabetes* (2016) 24(3). Epub 20161024.

33. Zhang SJ. A Study on the Current Situation of Chronic Diseases of Rural Residents Aged 35-70 in Shunyi District of Beijing and the Demand for Community Health Services and Control Strategies [dissertation thesis]: Chinese Center for Disease Control and Prevention (2007).

34. Zhang XQ, Liu X, Cao FY, Xue YZ. Psychosocial Factors Related to Quality of Life in Community Patients with Type 2 Diabetes. *China Modern Doctor* (2010) (2). Epub 20100420.

35. Zhang Y, Lv WJ, Li H. Analysis and Nursing of Depression in Diabetic Patients. *Journal of Qilu Nursing* (2012) (3). Epub 20120726.

36. Zhang ZF. Study on Health Status and Influencing Factors of Retired Cadres in a City [dissertation thesis]: Soochow University (2013).

37. Zhao HF, Li H, Huang HL, Wang MD. A Survey of Sickness of the Aged in City and Countryside Intersection of Fuzhou City and Influencing Factors. *Chinese Nursing Research* (2009) 023(003):767-8.

38. Zhong Y, Jia WP. Correlation between Glycated Hemoglobin and Early Cognitive Decline in Type 2 Diabetes Mellitus. *Chinese Journal of Geriatric Care* (2005) 3(4). Epub 20080330.

39. Zhou H. Clinical Study of Type 2 Diabetes Patients Complicated with Depression. *Sichuan Medical Journal* (2010) 31(04):486-7.

40. Bao XY, Xie YX, Zhang XX, Peng X, Huang JX, Du QF, et al. The Association between Multimorbidity and Health-Related Quality of Life: A Cross-Sectional Survey among Community Middle-Aged and Elderly Residents in Southern China. *Health and Quality of Life Outcomes* (2019) 17(1). doi: 10.1186/s12955-019-1175-0.

41. Garin N, Koyanagi A, Chatterji S, Tyrovolas S, Olaya B, Leonardi M, et al. Global Multimorbidity Patterns: A Cross-Sectional, Population-Based, Multi-Country Study. *J Gerontol A Biol Sci Med Sci* (2016) 71(2):205-14. Epub 2015/10/01. doi: 10.1093/gerona/glv128.

42. Jiang CH, Zhu F, Qin TT. Relationships between Chronic Diseases and Depression among Middle-Aged and Elderly People in China: A Prospective Study from Charls. *Curr Med Sci* (2020) 40(5):858-70. Epub 2020/10/31. doi: 10.1007/s11596-020-2270-5.

43. Liu Y, Meng H, Tu N, Liu D. The Relationship between Health Literacy, Social Support, Depression, and Frailty among Community-Dwelling Older Patients with Hypertension and Diabetes in China. *Front Public Health* (2020) 8:280. Epub 2020/07/28. doi: 10.3389/fpubh.2020.00280.

44. Nan H, Lee PH, McDowell I, Ni MY, Stewart SM, Lam TH. Depressive Symptoms in People with Chronic Physical Conditions: Prevalence and Risk Factors in a Hong Kong Community Sample. *BMC Psychiatry* (2012) 12. doi: 10.1186/1471-244X-12-198.

45. Xiu S, Liao Q, Sun L, Chan P. Risk Factors for Cognitive Impairment in Older People with Diabetes: A Community-Based Study. *Ther Adv Endocrinol Metab* (2019) 10:2042018819836640. Epub 2019/06/04. doi: 10.1177/2042018819836640.

46. Li Y, Li YF, Guo LF, Ma L, Tian QF. Common Chronic Comorbidity in the Elderly in Henan. *Modern Preventive Medicine* (2020) 47(15):2797-800.

47. Li X. A Survey of Calm Mood Status and Its Influencing Factors in Elderly Patients with Type 2 Diabetes Mellitus. *Journal of Practical Diabetology* (2020) 16(5). Epub 20210603.

48. Lu YF. Investigation and Analysis of the Physical Quality of the Elderly in Chengdu Branch Community. *Boxing & Fight* (2021) (8):106.

49. Xu CX, Xue JY, Chen R, Li WJ. Status and Influencing Factors of Depression among the Elderly in Taixing Nursing Institutions. *Chinese Journal of Gerontology* (2021) 41(9). Epub 20210910.

50. Wu LY. To Obtain Management Methods of Chronic Disease under the Background of Epidemic Situation [dissertation thesis]: Anhui Medical University (2021).

51. Fu C, Li Z, Mao Z. Association between Social Activities and Cognitive Function among the Elderly in China: A Cross-Sectional Study. *International Journal of Environmental Research and Public Health* (2018) 15(2). doi: 10.3390/ijerph15020231.

52. Fan R, Zhao L, Ding BJ, Xiao R, Ma WW. The Association of Blood Non-Esterified Fatty Acid, Saturated Fatty Acids, and Polyunsaturated Fatty Acids Levels with Mild Cognitive Impairment in Chinese Population Aged 35–64 Years: A Cross-Sectional Study. *Nutritional Neuroscience* (2021) 24(2):148-60. doi: 10.1080/1028415X.2019.1610606.

53. Jia Y, Liu R, Tang S, Zhang D, Wang Y, Cong L, et al. Associations of the Glycaemic Control of Diabetes with Dementia and Physical Function in Rural-Dwelling Older Chinese Adults: A Population-Based Study. *Clinical Interventions in Aging* (2021) 16:1503-13. doi: 10.2147/CIA.S319633.

54. Cai Y, Xu W, Xiao H, Liu H, Chen T. Correlation between Frailty and Adverse Outcomes among Older Community-Dwelling Chinese Adults: The China Health and Retirement Longitudinal Study. *Journal of Nutrition, Health and Aging* (2020) 24(7):752-7. doi: 10.1007/s12603-020-1368-6.

55. Kong L, Zhao H, Fan J, Wang Q, Li J, Bai J, et al. Predictors of Frailty among Chinese Community-Dwelling Older Adults with Type 2 Diabetes: A Cross-Sectional Survey. *BMJ Open* (2021) 11(3). doi: 10.1136/bmjopen-2020-041578.

56. Jia Z, Du X, Du J, Xia S, Guo L, Su X, et al. Prevalence and Factors Associated with Depressive and Anxiety Symptoms in a Chinese Population with and without Cardiovascular Diseases. *Journal of Affective Disorders* (2021) 286:241-7. doi: 10.1016/j.jad.2021.02.006.

57. Li HW, Lee WJ, Lin MH, Peng LN, Loh CH, Chen LK, et al. Quality of Life among Community-Dwelling Middle-Aged and Older Adults: Function Matters More Than Multimorbidity. *Archives of Gerontology and Geriatrics* (2021) 95. doi: 10.1016/j.archger.2021.104423.

58. Liu C. Quality of Life Evaluation and Influencing Factors of Type 2 Diabetes Mellitus [dissertation thesis]: Harbin Medical University (2010).

**Duplicate publication or data (n = 15)**

1. Fan LS, Gao M, FISHER EB, et al. Factors associated with quality of life in 747 patients with type 2 diabetes in Tongzhou District and Shunyi District of Beijiing. Journal of Peking University(Health Sciences. 2021;53(3).

2. Wang FB, Gao M, Chen XY, et al. Relation of depression to quality of life and family support in patients with type 2 diabetes in community. Chinese Mental Health Journal. 2020;34(7).

3. Chen L, Wu CK, Peng C, et al. Association Between Chronic Diseases and Depressive Symptom in Middle-aged and Elderly People over 45 Years Old in China. Medicine and Society. 2021;34(10):90-4,9.

4. Huang ZT, Luo Y, Su HX, et al. Relationship between cardiovascular metabolic multimorbidity and depressive symptoms in Chinese older adults. Chinese Journal of Prevention and Control of Chronic Diseases. 2021;29(9):644-8.

5. Huang ZT, Luo Y, Han L, et al. Patterns of cardiometabolic multimorbidity and the risk of depressive symptoms in a longitudinal cohort of middle-aged and older Chinese. Journal of Affective Disorders. 2022;301:1-7.

6. Gao J, Zeng QZ, He YL, et al. Positive rate and associated factors of anxiety and depressive symptoms among community-dwelling hypertension and diabetes patients. Chinese Journal of Public Health 2018;34(2):223-9.

7. Li Y, Jia R, Li XC. Community based cross-sectional survey on patients with Type 2 diabetes complicated with depression. Chinese Health Service Management. 2016;33(02):94-6.

8. Si Q. Bidirectional association between depression and type 2 diabetes mellitus in adults [dissertation thesis]: Shandong University; 2015.

9. Sun XR, Yuan J, Qu ZW, et al. Investigation of depressive disorder in community elderly with diabetes mellitus. Journal of Clinical Psychiatry. 2017;27(04):238-40.

10. Zhao J, Lou PA. Sleep quality and risk factors in type 2 diabetes mellitus. Jiangsu Journal of Preventive Medicine 2014;25(3).

11. Zhao J, Lou PA. Correlation between self-management positivity and quality of life in patients with type 2 diabetes mellitus. Chinese Journal of Modern Nursing. 2016(2).

12. Zhao J, Lou PA, Zhang P, et al. Status and risk factors of anxiety and depression in type 2 diabetes mellitus. Chinese Journal of Diabetes. 2014;22(07):615-9.

13. Liu Y, Maier M, Hao Y, et al. Factors related to quality of life for patients with type 2 diabetes with or without depressive symptoms - results from a community-based study in China. J Clin Nurs. 2013;22(1-2):80-8.

14. Ning F, Wang YM, Zhang D, et al., editors. Depression increases the risk of developing type 2 diabetes. The 7th National Epidemiology Academic Conference & Epidemiology Branch of Chinese Preventive Medicine Association; 2014; Nanjing, Jiangsu, China.

15. Ning F, Wang YM, Zhang D, et al. Study on the correlation between depression and type 2 diabetes. Proceedings of the 18th National Conference of Diabetes Society of Chinese Medical Association; Guangzhou, China2014. p. 282-.

**Unknown source of population (*n* = 9)**

1. Zhang C, Wu JP, Li JN, Guo XD, Liu SH, Zeng YM. Psychological Status Analysis of Patients with Diabetes During Covid-19 Epidemic. *Chinese General Practice Nursing* (2020) 18(8). Epub 20200804.

2. Liao HF, Yang SQ, Chen XC. Investigation and Analysis of Depression Symptoms in 167 Patients with Type 2 Diabetes Mellitus. *The Journal of Practical Medicine* (2002) 18(6). Epub 20021231.

3. Lin T, Qin XQ, Wang J. Correlation Analysis between Depression and Self-Care Behavior in Type 2 Diabetes Patients. *Journal of Qilu Nursing* (2008) 14(3). Epub 20080720.

4. Ma S, Li L. Analysis of 86 Cases of Depressive Disorder with Type 2 Diabetes. *The Journal of Medical Theory and Practice* (2004) 17(4):409-10. doi: 10.3969/j.issn.1001-7585.2004.04.023.

5. Wang P, Cai HW. Analysis of Depression in Patients with Incipient Type 2 Diabetes Mellitus. *National internal medicine nursing academic exchange and thematic lecture conference papers*; Kunming, China(2003).

6. Xu JH. Investigation on Anxiety and Depression of Elderly Diabetic Patients in Community. *Journal of Modern Medicine & Health* (2019) 35(S1). Epub 20200804.

7. Zhang DY, Ma QH. Risk Factors for Depression in Type 2 Diabetes Patients. *Journal of Practical Diabetology* (2018) 14(6). Epub 20190805.

8. Zhang Y, Lv WJ, Li H. Analysis and Nursing of Depression in Diabetic Patients. *Journal of Qilu Nursing* (2012) (3). Epub 20120726.

9. Zhao S, Mao H, Fu AD, Wang HX, Yao CW. A Study on Depression among Diabetic Population. *Chinese Journal of Diabetes* (2002) (05):17-20.
