# Supplementary material for: A Systematic Review and Meta-Analysis of the Prevalence and Risk Factors of Depression in Type 2 Diabetes Patients in China
Source: Front Med (Lausanne). 2022 May 10;9:759499. doi: 10.3389/fmed.2022.759499 (PMC9127805; doi:10.3389/fmed.2022.759499)
Supplement: Supplementary File 4 — Risk of bias assessment results of included studies. [file Data_Sheet_4.docx]

**Risk of bias assessment results of included studies**

| Studies | the Agency for Healthcare Research and Quality (AHRQ) methodology checklist | | | | | | | | | | | |
| --- | --- | --- | --- | --- | --- | --- | --- | --- | --- | --- | --- | --- |
|  | Item 1 | Item 2 | Item 3 | Item 4 | Item 5 | Item 6 | Item 7 | Item 8 | Item 9 | Item 10 | Item 11 | Scores |
| Chou and Chi (32) | Yes | Yes | Yes | Yes | Unclear | Unclear | Unclear | Unclear | No | Yes | No | 5 |
| Xu (33) | Yes | Yes | Yes | Yes | Yes | Yes | No | Yes | No | Yes | No | 8 |
| Huang et al. (34) | Yes | Yes | Yes | Yes | Unclear | Unclear | No | No | No | Yes | No | 5 |
| Sun and Dong (35) | Yes | No | No | Yes | Unclear | Unclear | Unclear | No | No | No | No | 2 |
| Chen et al. (36) | Yes | Yes | No | Yes | Unclear | Unclear | Unclear | Yes | No | No | No | 4 |
| Liu et al. (37) | Yes | Yes | No | Yes | Unclear | Unclear | Yes | No | No | Yes | No | 5 |
| Qian et al. (38) | Yes | Yes | No | Yes | Unclear | Unclear | No | Yes | No | Yes | No | 5 |
| Yang et al.  (39) | Yes | Yes | Yes | Yes | Unclear | Unclear | No | Yes | No | Yes | No | 6 |
| Zhang (40) | Yes | Yes | Yes | Yes | Unclear | Unclear | No | No | No | No | No | 4 |
| Wang (41) | Yes | Yes | No | Yes | Unclear | Unclear | No | No | No | Yes | No | 4 |
| Liu et al. (42) | Yes | Yes | Yes | Yes | Unclear | Unclear | Yes | Yes | No | Yes | No | 7 |
| Mezuk et al. (43) | Yes | Yes | Yes | Yes | Unclear | Yes | Yes | Yes | Yes | Yes | No | 9 |
| Wang et al. (44) | Yes | Yes | Yes | Yes | Unclear | Unclear | No | Yes | No | No | No | 5 |
| Xie (45) | Yes | Yes | No | Yes | Unclear | Yes | No | Yes | No | Yes | No | 6 |
| Xu et al. (46) | Yes | No | No | Yes | Unclear | No | No | No | No | Yes | No | 3 |
| Zheng et al. (47) | Yes | Yes | Yes | Yes | Unclear | Unclear | Yes | Yes | No | No | No | 6 |
| Wang et al. (48) | Yes | Yes | No | Yes | Unclear | Unclear | Yes | Unclear | No | Yes | No | 5 |
| Sun et al. (49) | Yes | Yes | Yes | Yes | Yes | Unclear | Yes | Yes | Yes | Yes | No | 9 |
| Sun et al. (22) | Yes | Yes | Yes | Yes | Unclear | Unclear | Yes | Yes | No | Yes | No | 7 |
| Li et al. (50) | Yes | Yes | Yes | Yes | Unclear | Unclear | Unclear | Unclear | Unclear | Yes | No | 5 |
| Ning et al. (51) | Yes | Yes | Yes | Yes | Unclear | Unclear | Unclear | Yes | No | Yes | No | 6 |
| Zhang et al. (52) | Yes | Yes | Yes | Yes | Unclear | No | Yes | No | No | Yes | No | 6 |
| Huang et al. (53) | Yes | Yes | Yes | Yes | Unclear | Unclear | No | No | No | Yes | No | 5 |
| Li et al. (54) | Yes | Yes | Yes | Yes | Unclear | Unclear | No | No | No | Yes | No | 5 |
| Ni and Liu (55) | Yes | Yes | No | Yes | Unclear | Unclear | No | No | No | Yes | No | 4 |
| Yang et al. (56) | Yes | Yes | Yes | Yes | Unclear | Unclear | No | No | No | Yes | No | 5 |
| Liu et al. (23) | Yes | Yes | Yes | Yes | Yes | Unclear | No | Unclear | No | Yes | No | 6 |
| Li et al. (24) | Yes | Yes | Yes | Yes | Yes | Unclear | No | Yes | No | Yes | No | 7 |
| Lee et al. (57) | Yes | Yes | Yes | Yes | Unclear | Unclear | Unclear | Unclear | No | Yes | No | 5 |
| Li et al. (58) | Yes | Yes | No | Yes | Unclear | Unclear | No | Unclear | No | No | No | 3 |
| Tang et al. (59) | Yes | Yes | Yes | Yes | Unclear | Unclear | Unclear | Unclear | No | Yes | No | 5 |
| Fu et al. (60) | Yes | Yes | No | Yes | Unclear | Unclear | No | Unclear | No | No | No | 3 |
| Ren (61) | Yes | Yes | Yes | Yes | Unclear | Unclear | Yes | Unclear | No | Yes | No | 6 |
| Sun et al. (62) | Yes | Yes | Yes | Yes | No | No | No | No | No | No | No | 4 |
| Zhang et al. (63) | Yes | Yes | Yes | Yes | Unclear | No | No | No | No | No | No | 4 |
| Zhang and Zhang  (64) | Yes | Yes | Yes | Yes | Unclear | Unclear | Unclear | No | No | Yes | No | 5 |
| Xiu et al. (65) | Yes | Yes | Yes | Yes | Yes | Unclear | No | No | No | Yes | No | 6 |
| Zhang et al. (66) | Yes | Yes | Yes | Yes | Unclear | Unclear | No | No | No | Yes | No | 5 |
| Zhang et al. (67) | Yes | Yes | Yes | Yes | Unclear | Unclear | No | No | No | Yes | No | 5 |
| Xu et al. (68) | Yes | Yes | Yes | Yes | Unclear | Unclear | No | Yes | No | Yes | No | 6 |
| Abdulai et al. (69) | Yes | Yes | Yes | Yes | Yes | Unclear | Yes | Yes | No | Yes | No | 8 |
| Kong et al. (70) | Yes | Yes | Yes | Yes | Unclear | Unclear | Unclear | Unclear | No | Yes | No | 5 |
| Gao et al. (71) | Yes | Yes | No | Yes | Unclear | Unclear | Yes | Unclear | No | Yes | No | 5 |
| Pan et al. (72) | Yes | Yes | Yes | Yes | Yes | Yes | No | Unclear | No | No | No | 6 |
| Wu et al. (73) | Yes | Yes | Yes | Yes | Unclear | Unclear | Unclear | Unclear | No | Yes | No | 5 |
| Yang and Wu (74) | Yes | Yes | Yes | Yes | Unclear | Yes | No | No | No | Yes | No | 6 |
| Liu et al. (75) | Yes | Yes | Yes | Yes | Unclear | Yes | Yes | Unclear | No | Yes | No | 7 |
| Ji et al. (76) | Yes | Yes | Yes | Yes | Unclear | Unclear | Unclear | Unclear | No | No | No | 4 |
| Item 1: Define the source of information (survey, record review);  Item 2: List inclusion and exclusion criteria for exposed and unexposed subjects (cases and controls) or refer to previous publications;  Item 3: Indicate time period used for identifying patients;  Item 4: Indicate whether or not subjects were consecutive if not population-based;  Item 5: Indicate if evaluators of subjective components of study were masked to other aspects of the status of the participants;  Item 6: Describe any assessments undertaken for quality assurance purposes (e.g., test/retest of primary outcome measurements);  Item 7: Explain any patient exclusions from analysis;  Item 8: Describe how confounding was assessed and/or controlled;  Item 9: If applicable, explain how missing data were handled in the analysis;  Item 10: Summarize patient response rates and completeness of data collection;  Item 11: Clarify what follow-up, if any, was expected and the percentage of patients for which incomplete data or follow-up was obtained. | | | | | | | | | | | | |
