# Supplementary material for: A Systematic Review and Meta-Analysis of the Prevalence and Risk Factors of Depression in Type 2 Diabetes Patients in China
Source: Front Med (Lausanne). 2022 May 10;9:759499. doi: 10.3389/fmed.2022.759499 (PMC9127805; doi:10.3389/fmed.2022.759499)

**Figure S1 Forest plot of the prevalence of depression in patients with type 2 diabetes mellitus (T2DM) according to gender**

(A) Women


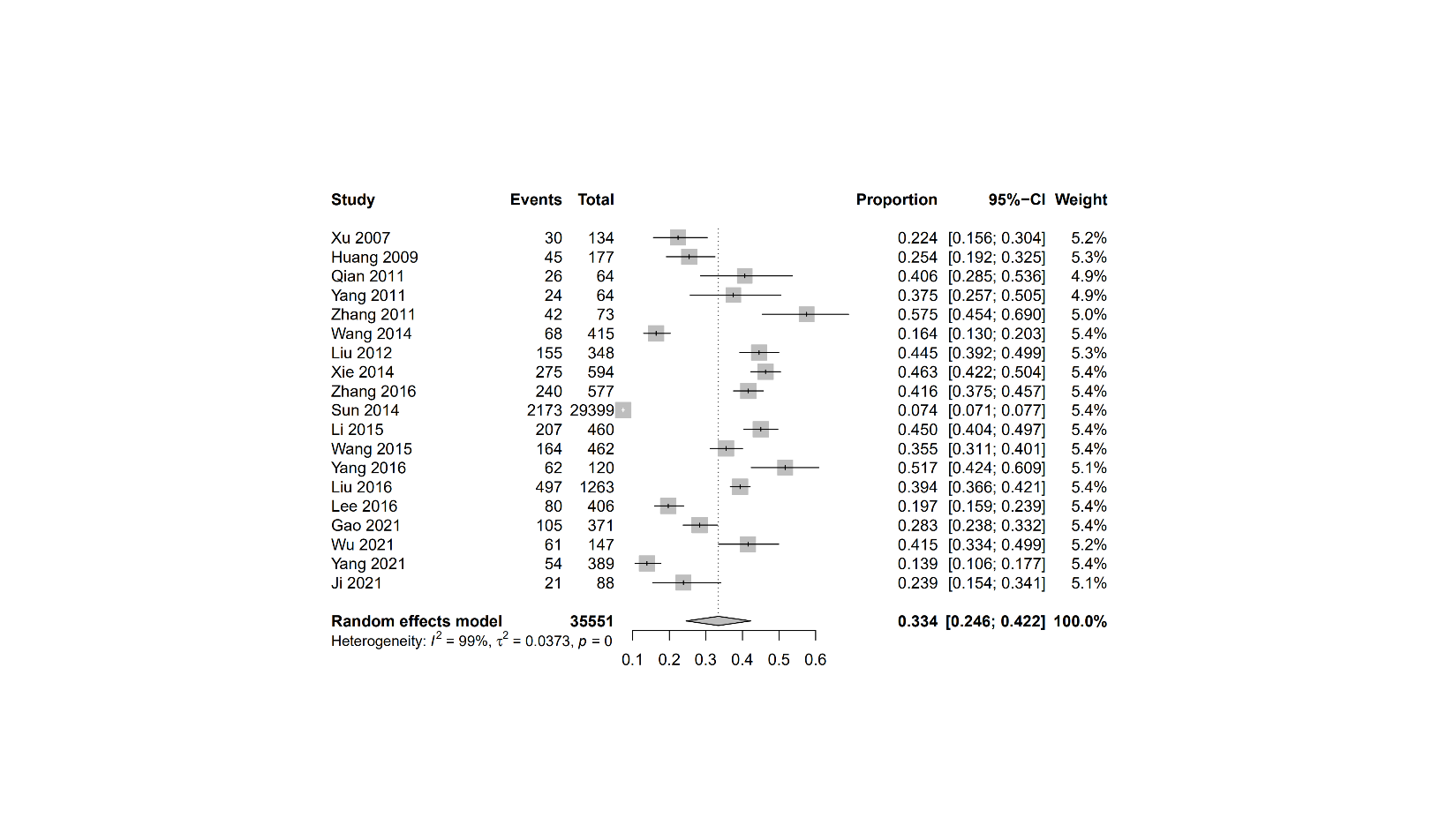


(B) Men


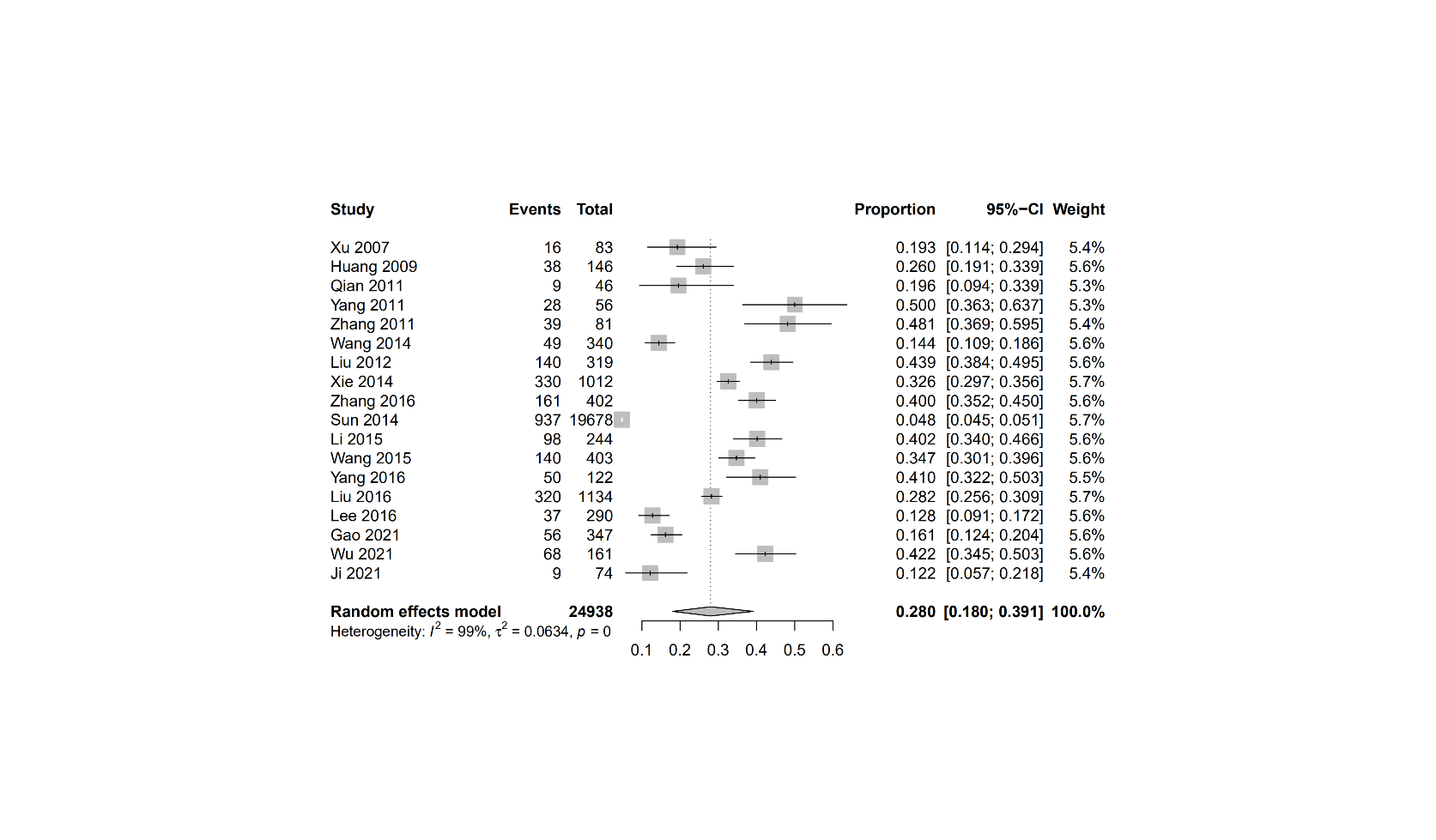


(C) Odds ratio (OR) (women vs. men)


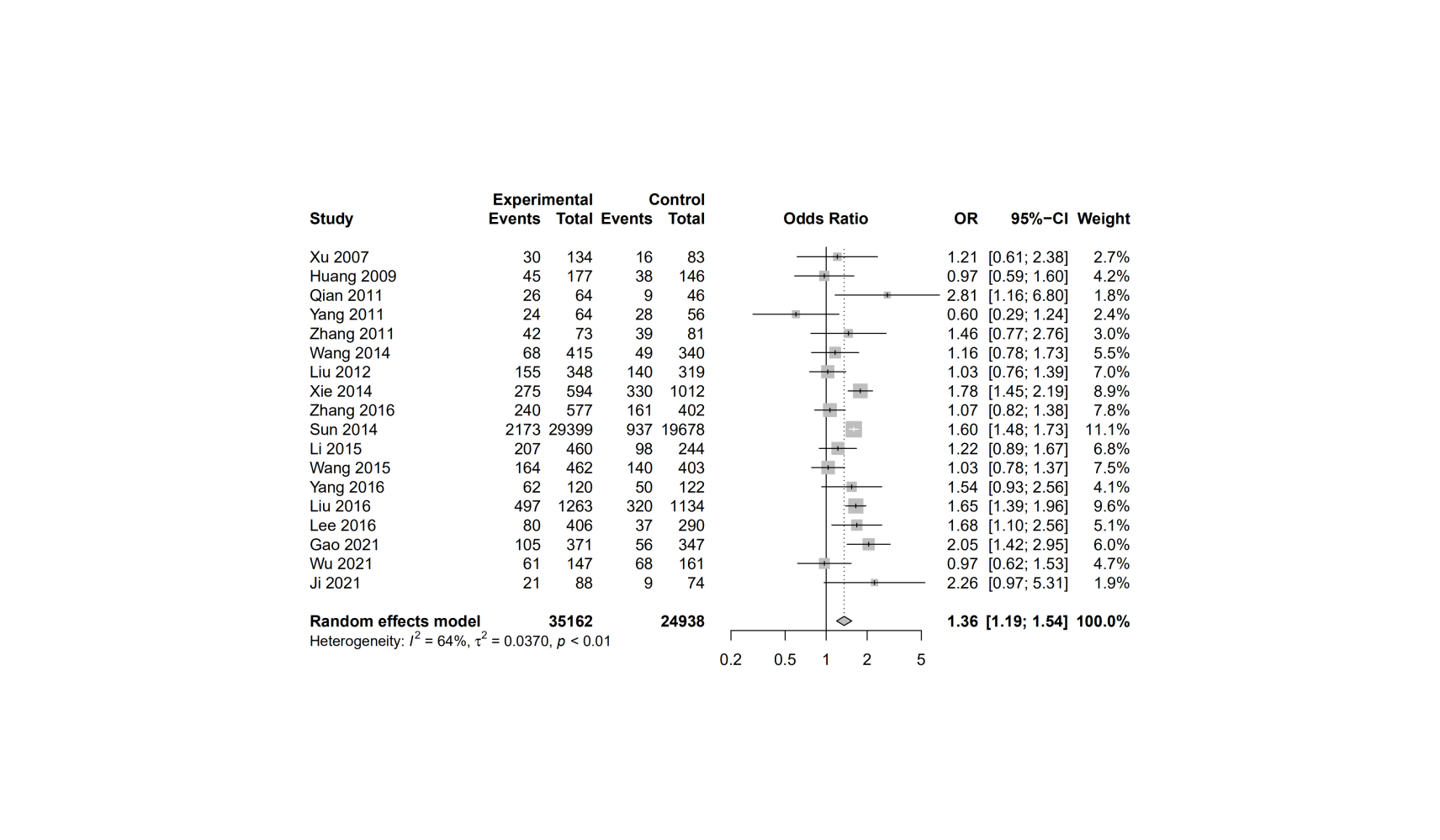


**Figure S2 Forest plot of the prevalence of depression in patients with T2DM according to age**

(A) Patients with T2DM aged ≥ 60 years


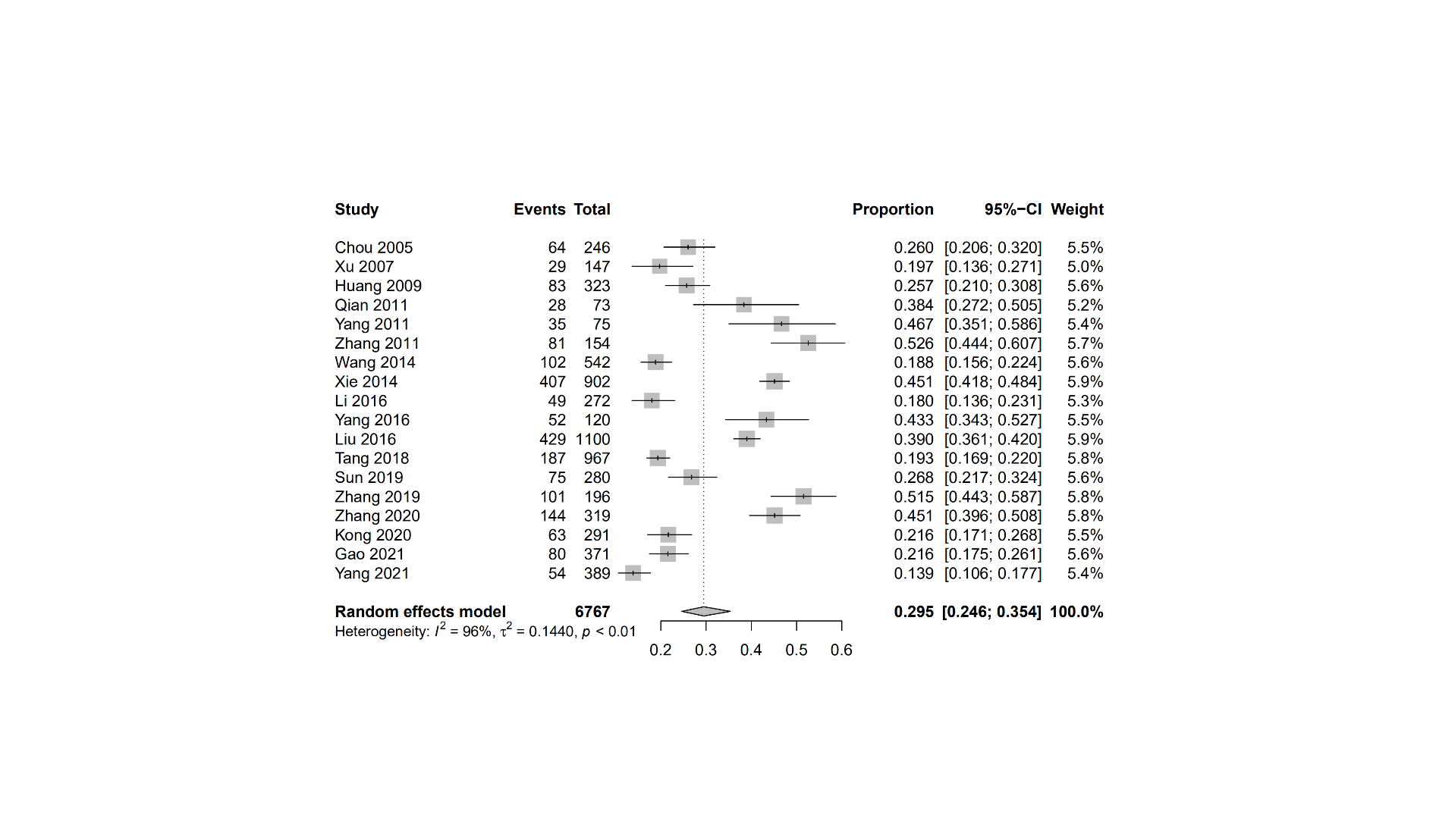


(B) Patients with T2DM aged <60 years


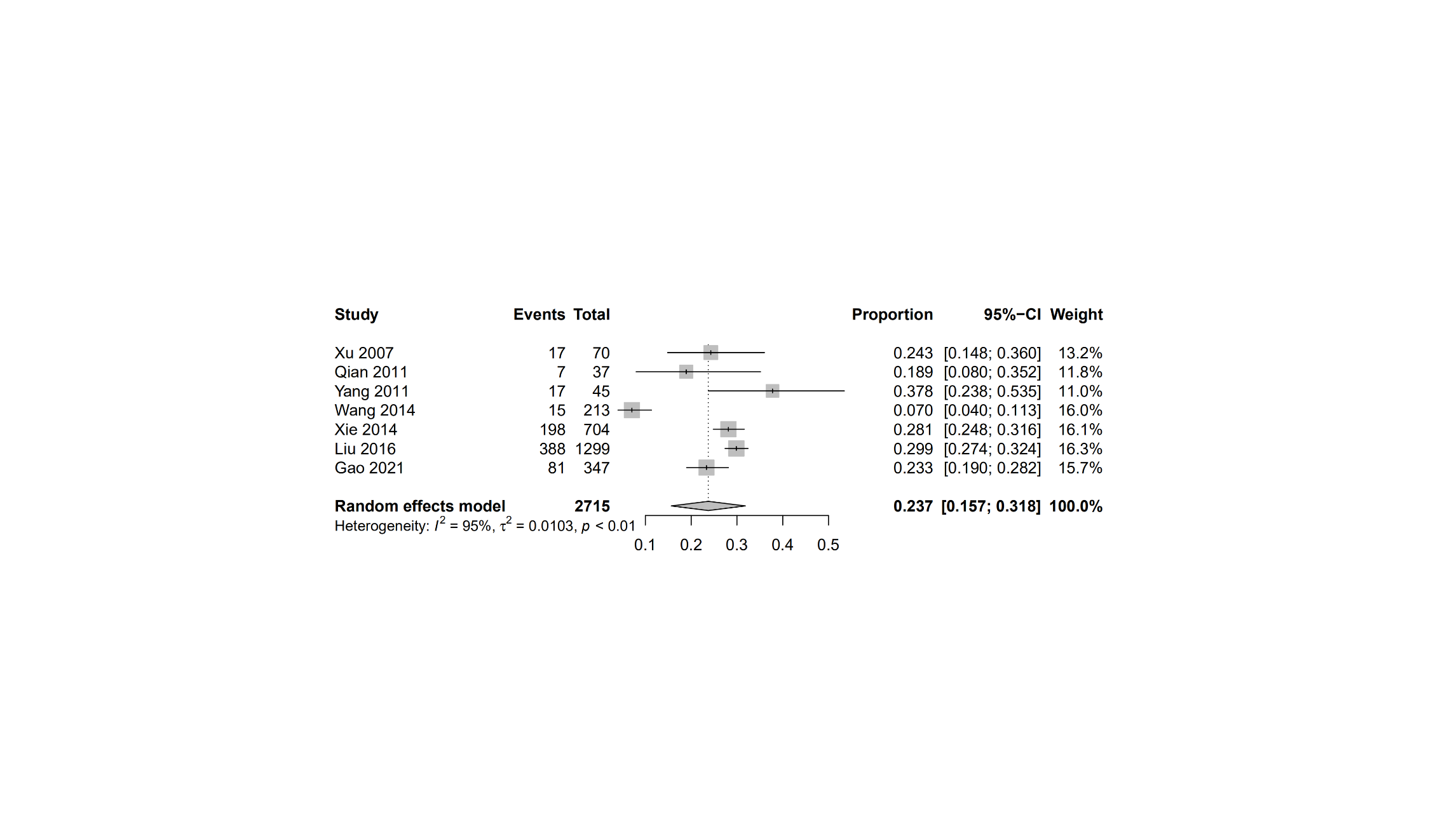


(C) OR (≥ 60 years old vs. <60 years old)


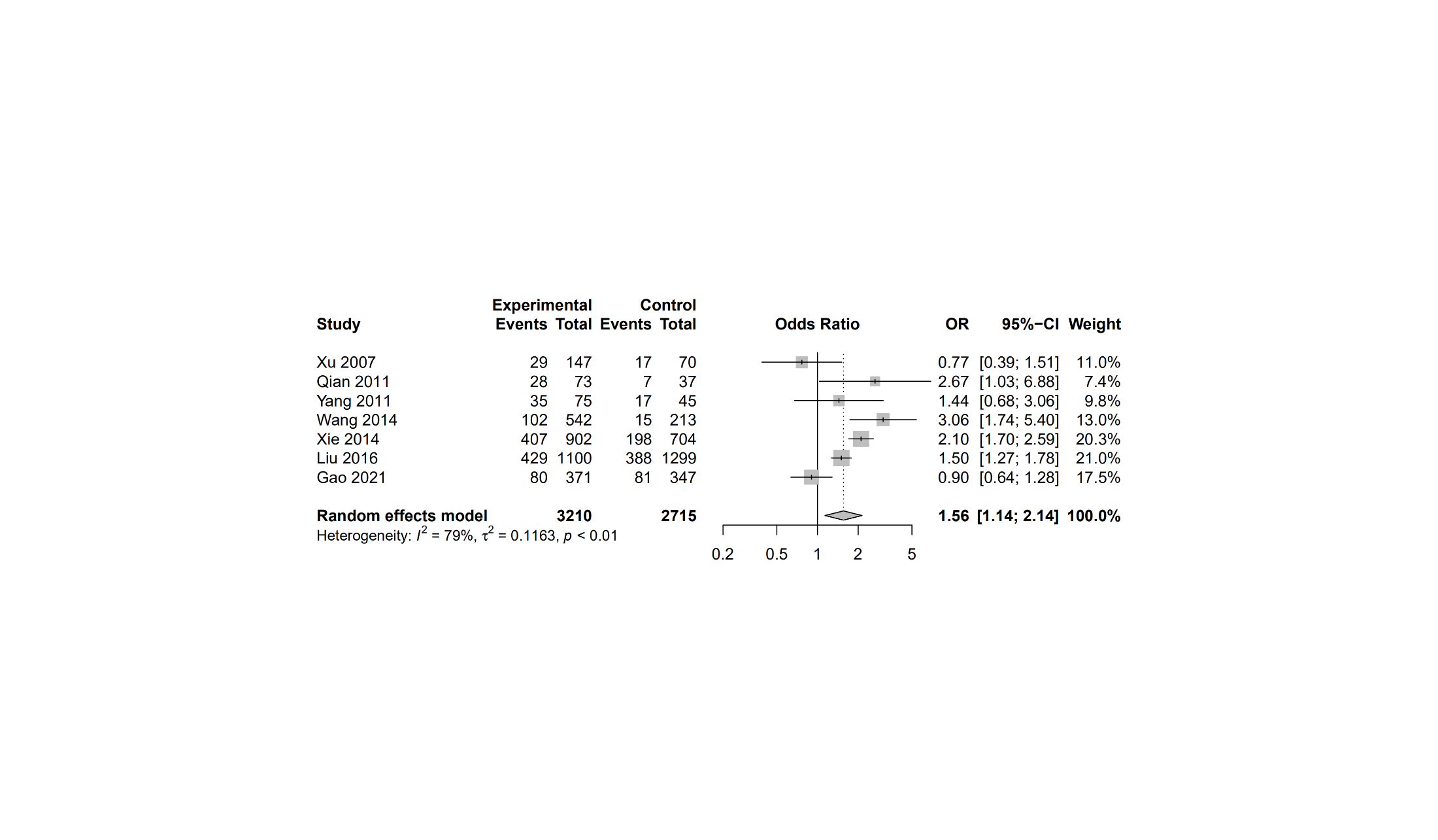


**Figure S3 Forest plot of the prevalence of depression in patients with T2DM according to educational level**

(A) Primary school or lower education level


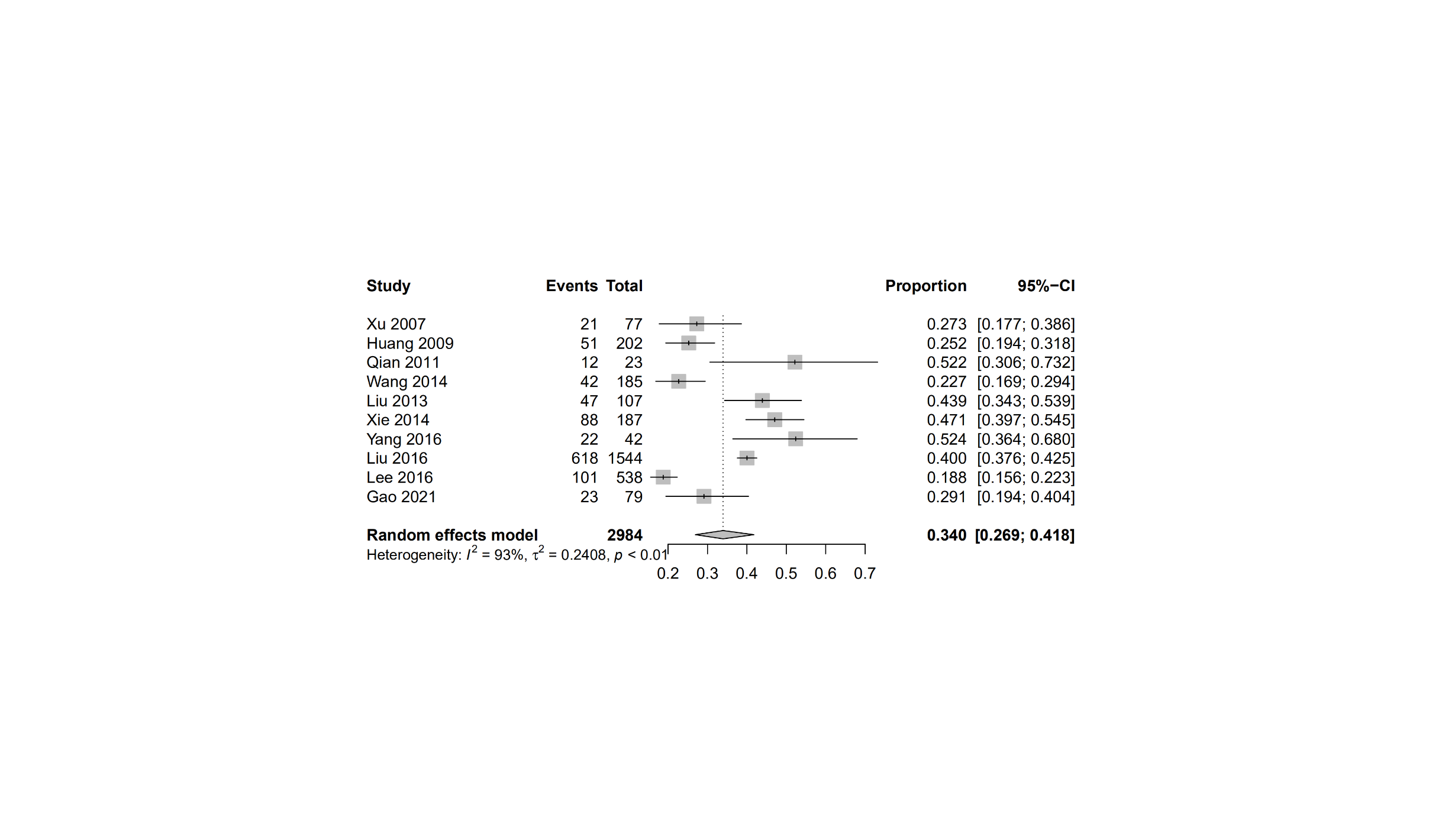


(B) Middle or high school education level


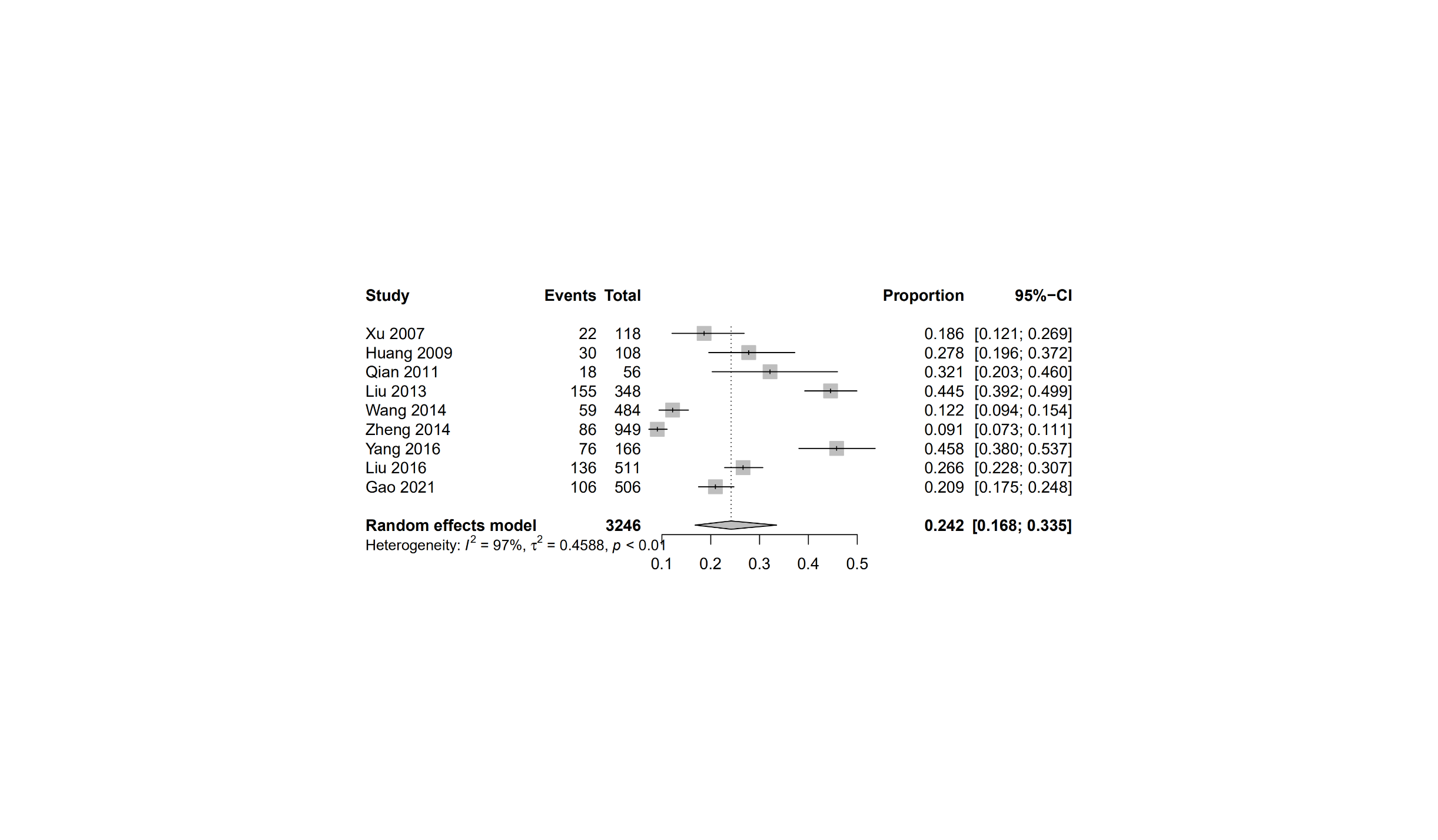


(C) College degree or higher education level


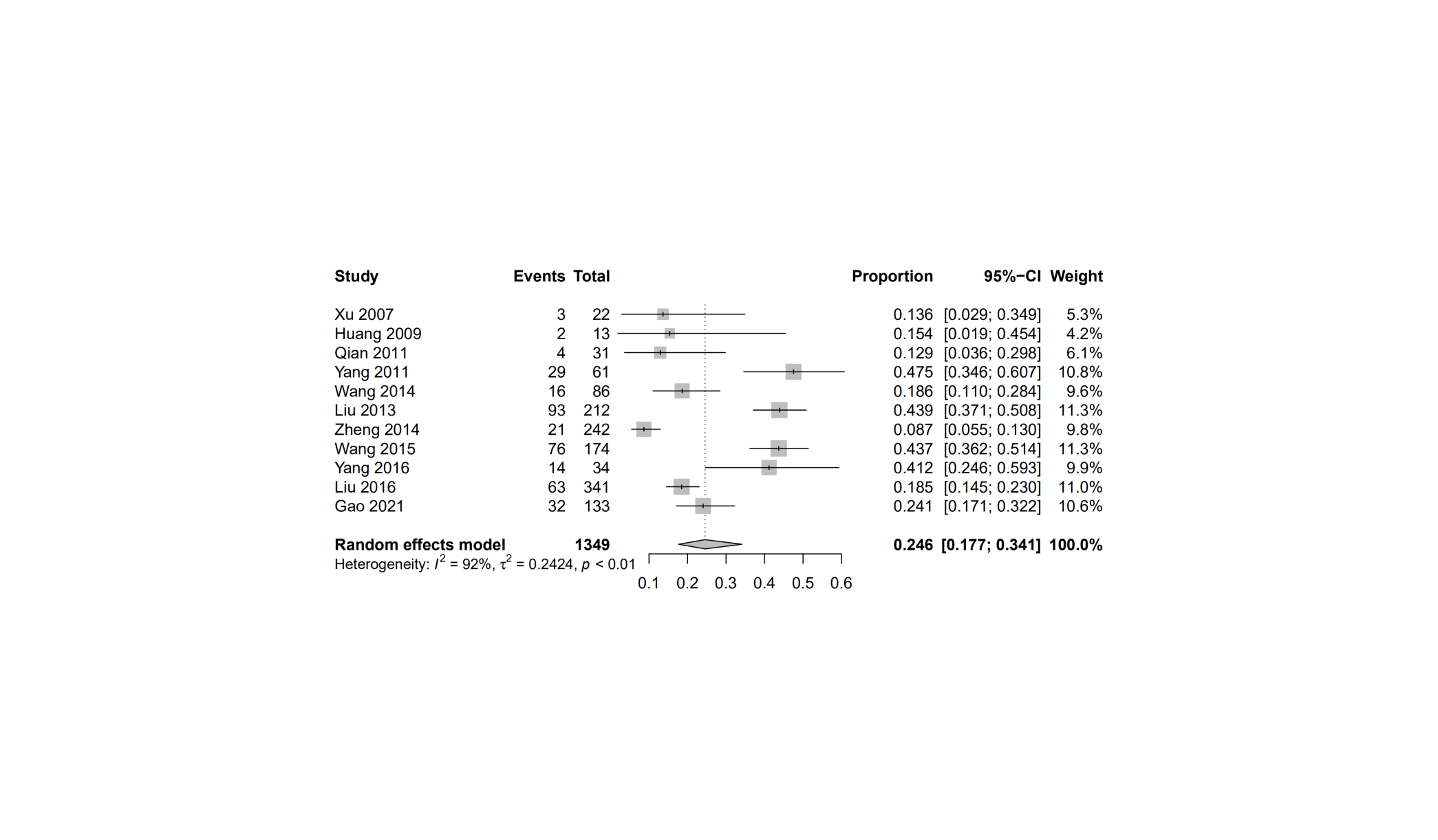


(D) OR (Primary school or lower education level vs. Middle or high school education level)


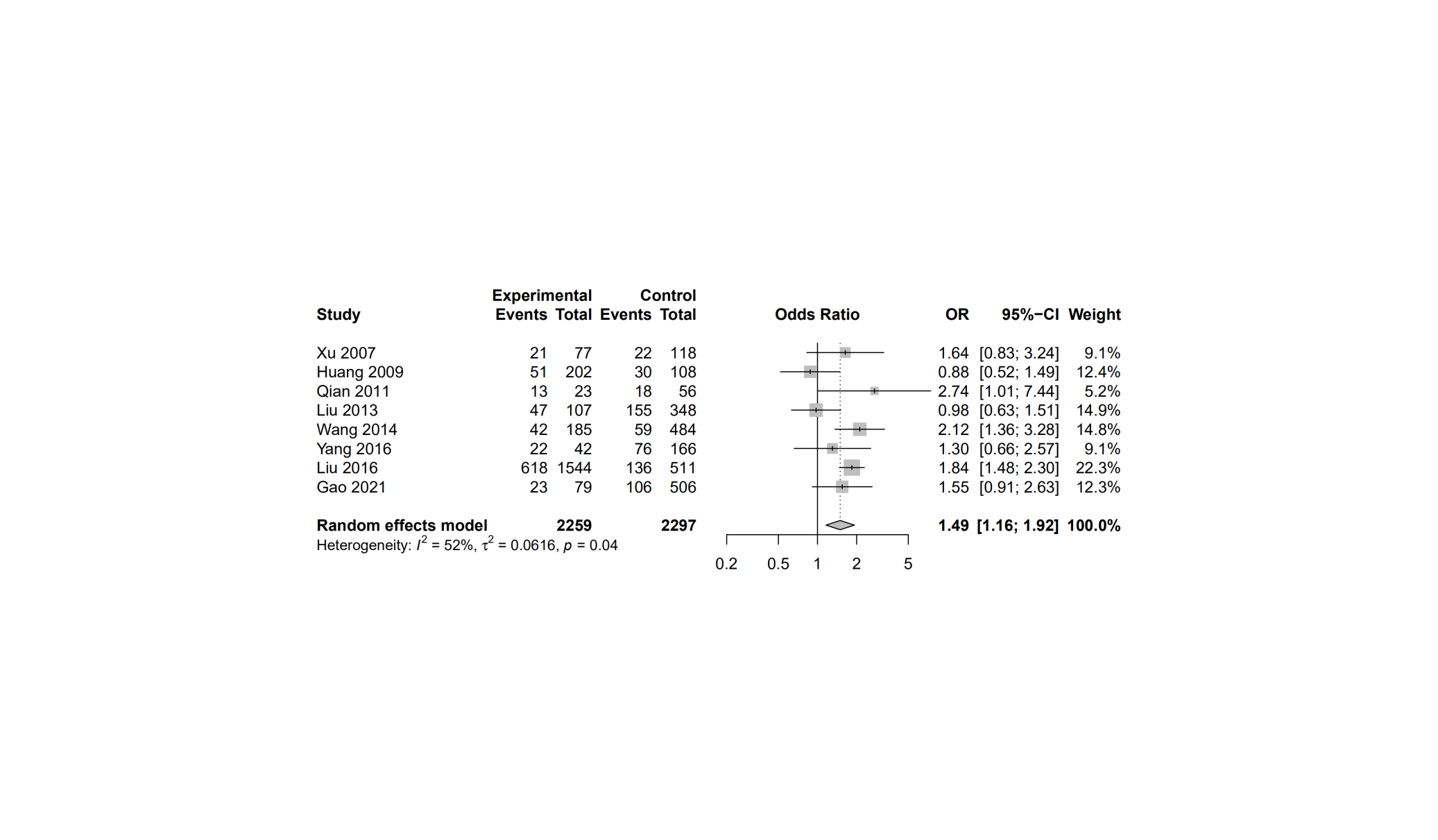


(E) OR (Primary school or lower education level vs. College degree or higher education level)


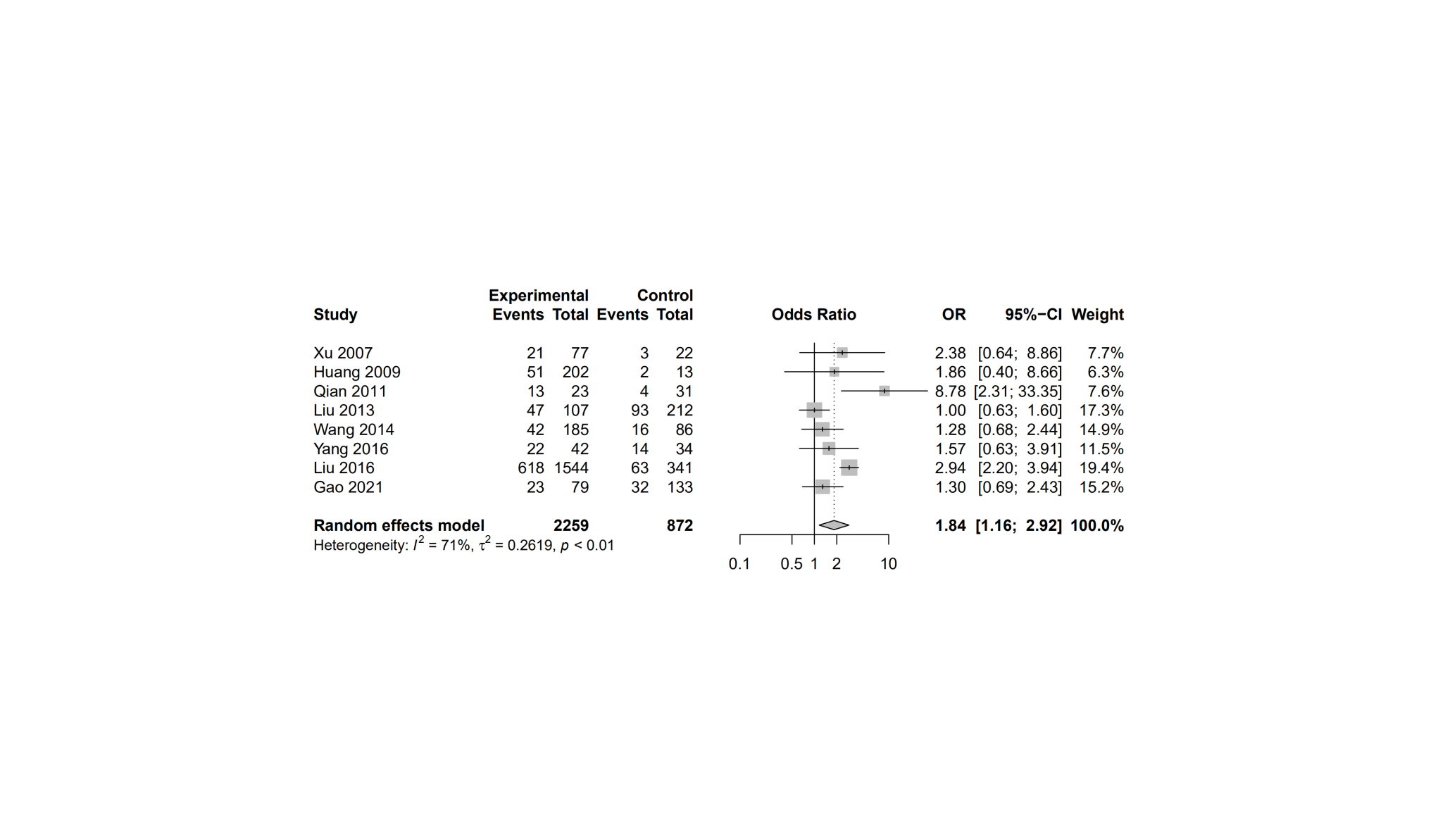


**Figure S4 Forest plot of the prevalence of depression according to residence**

(A) Urban residents


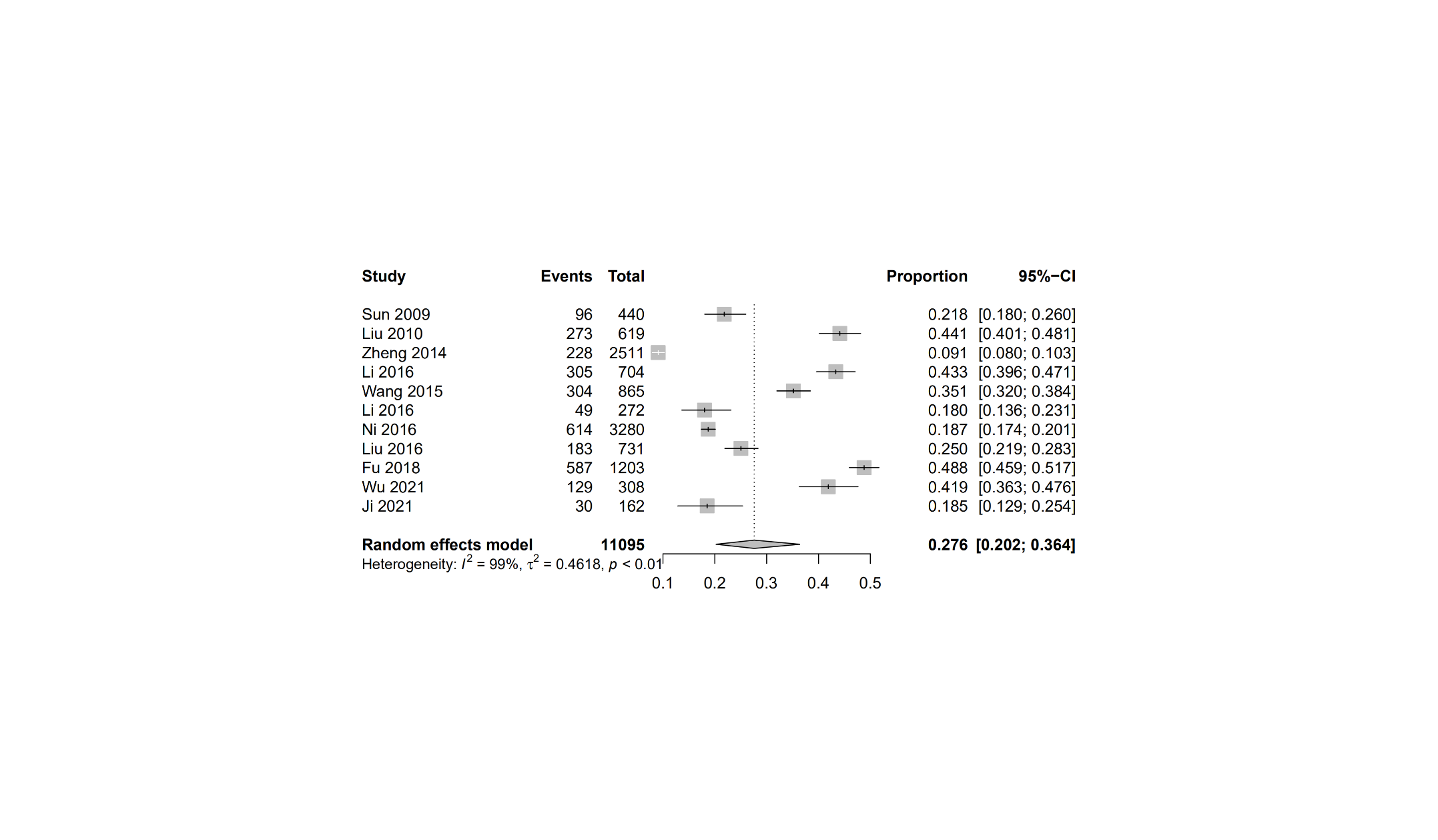


(B) Rural residents


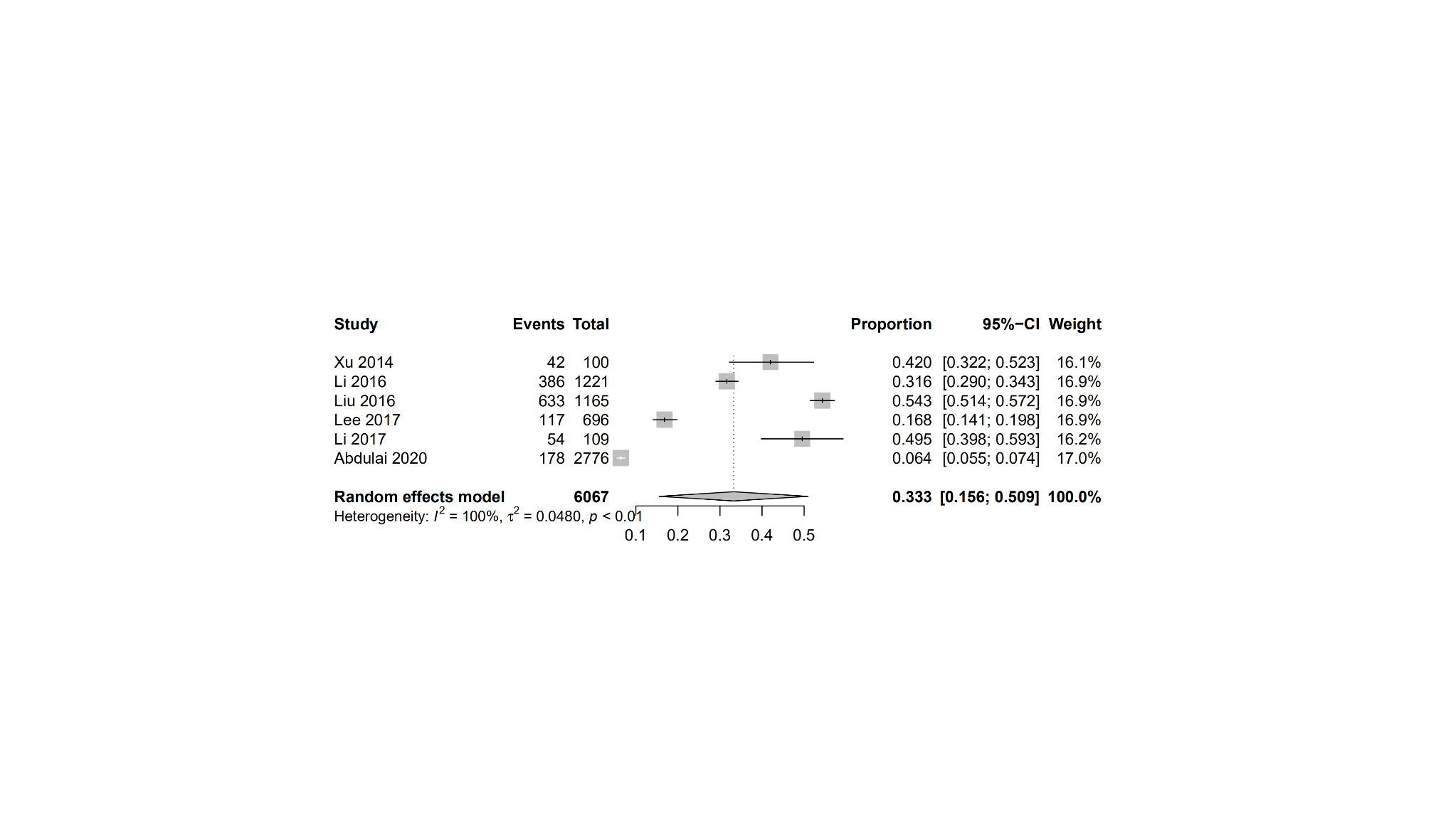


**Figure S5 Forest plot of the prevalence of depression in undiagnosed and diagnosed T2DM patients**

(A) Undiagnosed T2DM


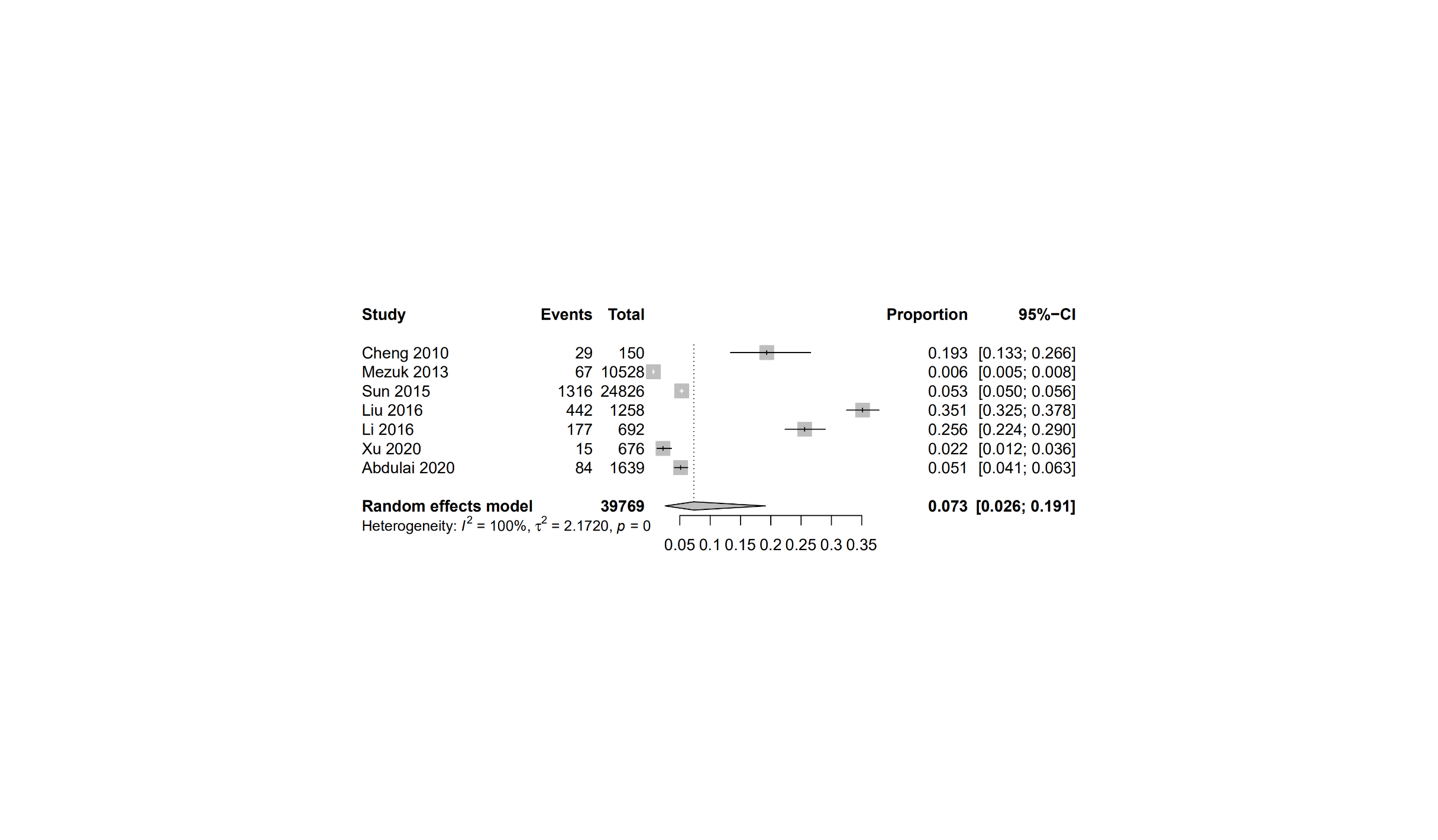


(B) Diagnosed T2DM


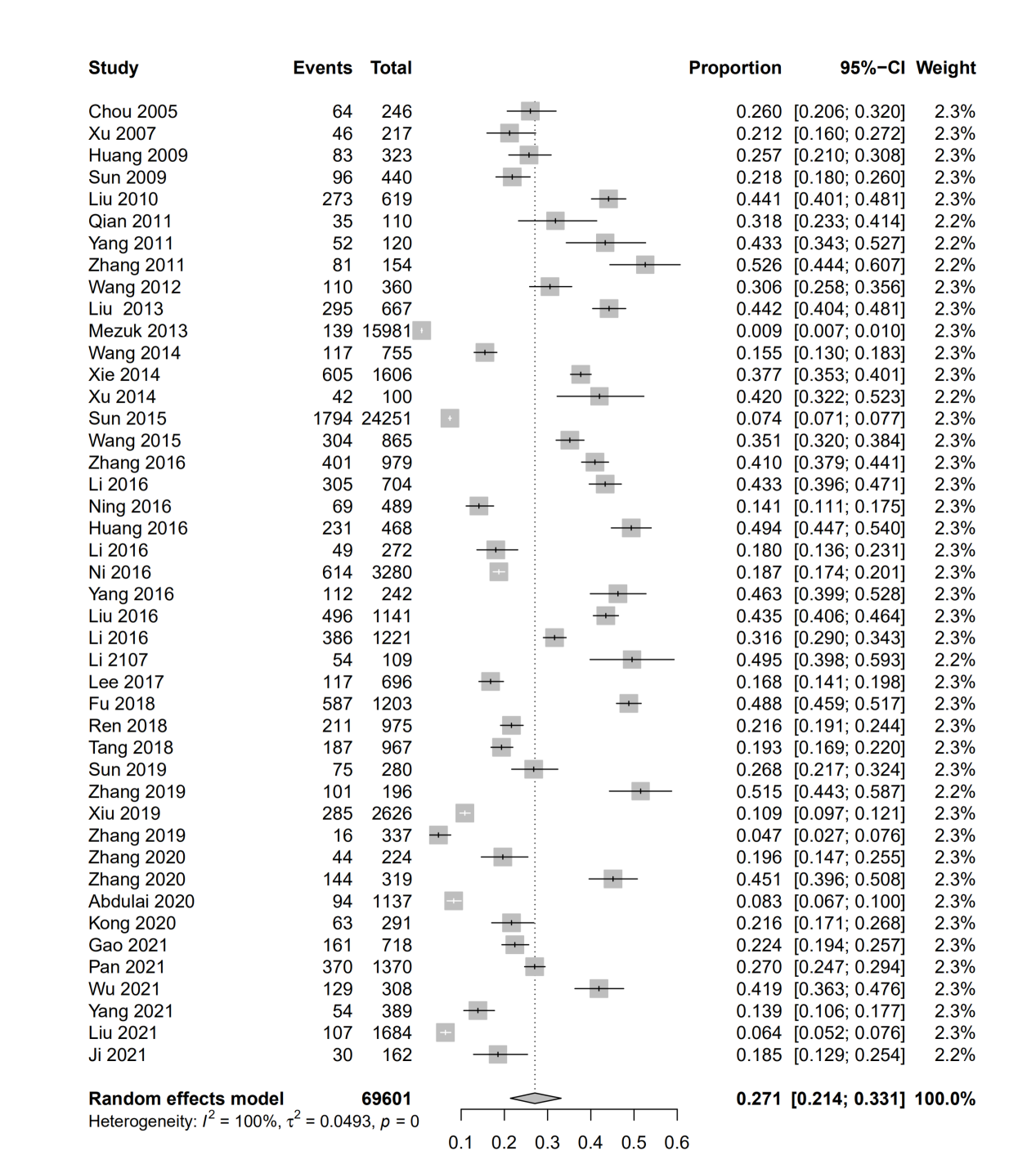


**Figure S6 Forest plot of the prevalence of depression according to the duration of T2DM**

(A) Duration <5 years


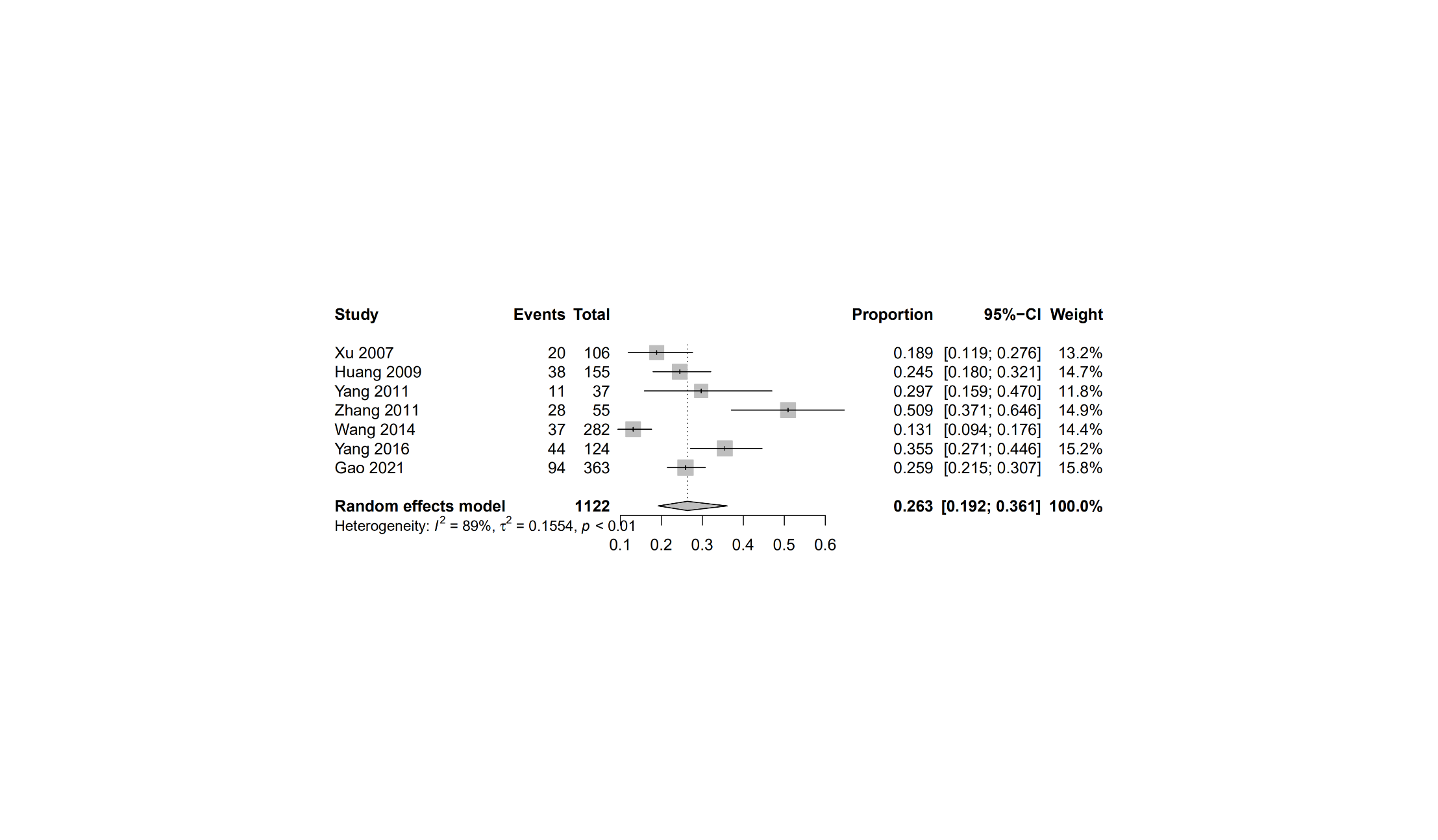


(B) Duration between 5 and 10 years


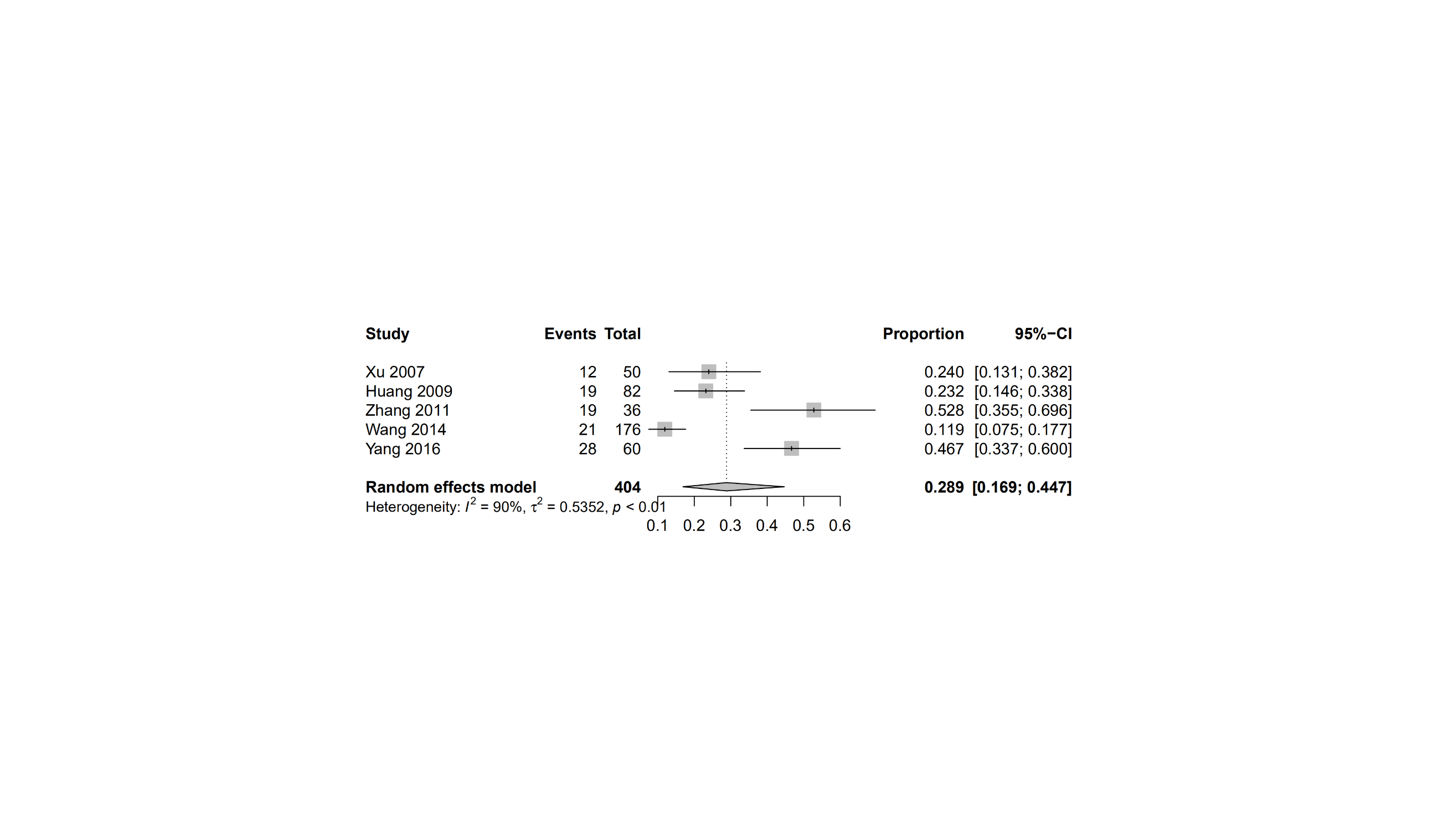


(C) ≥10 years


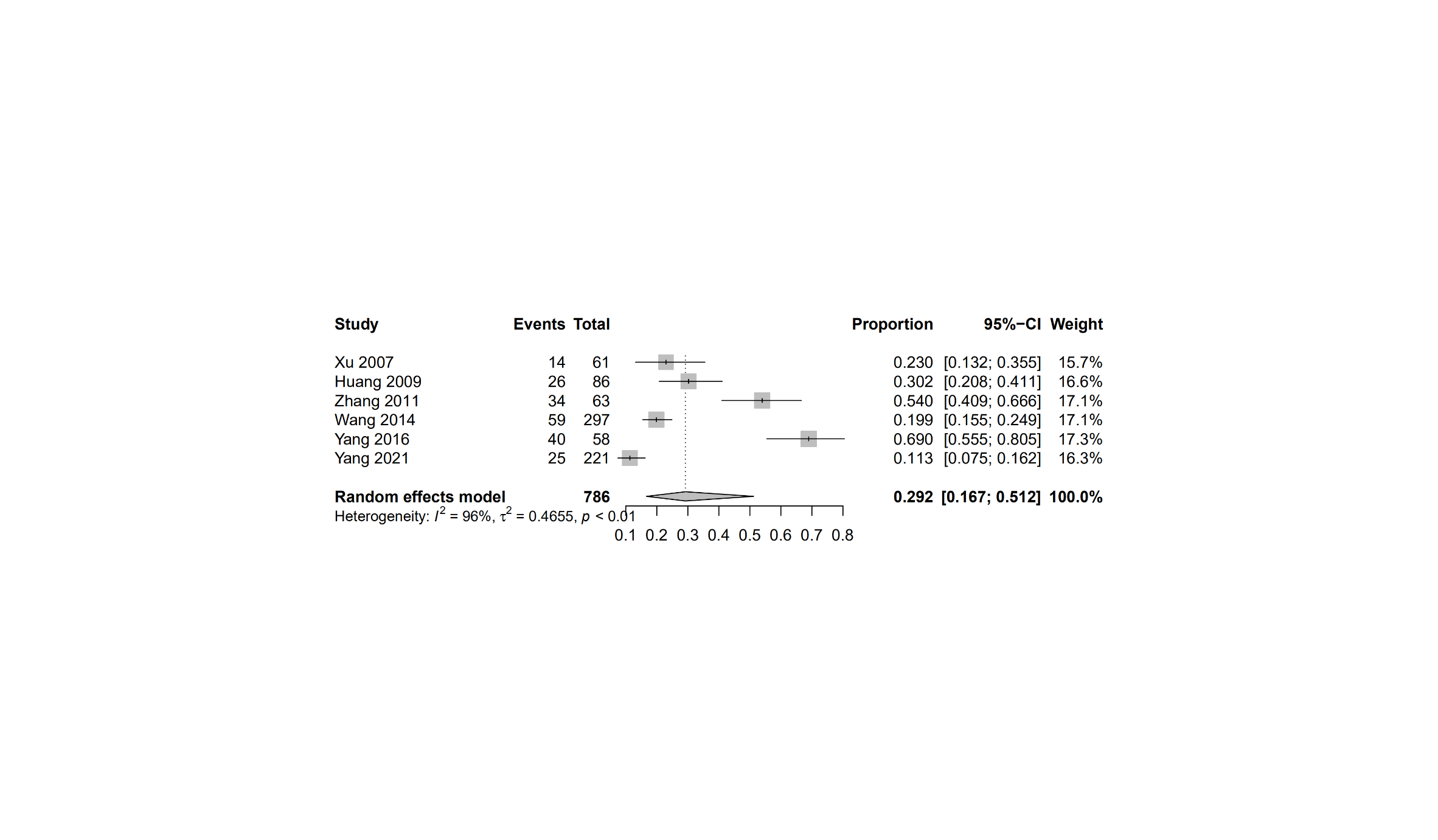


(D) OR (5–10 years vs. <5 years)


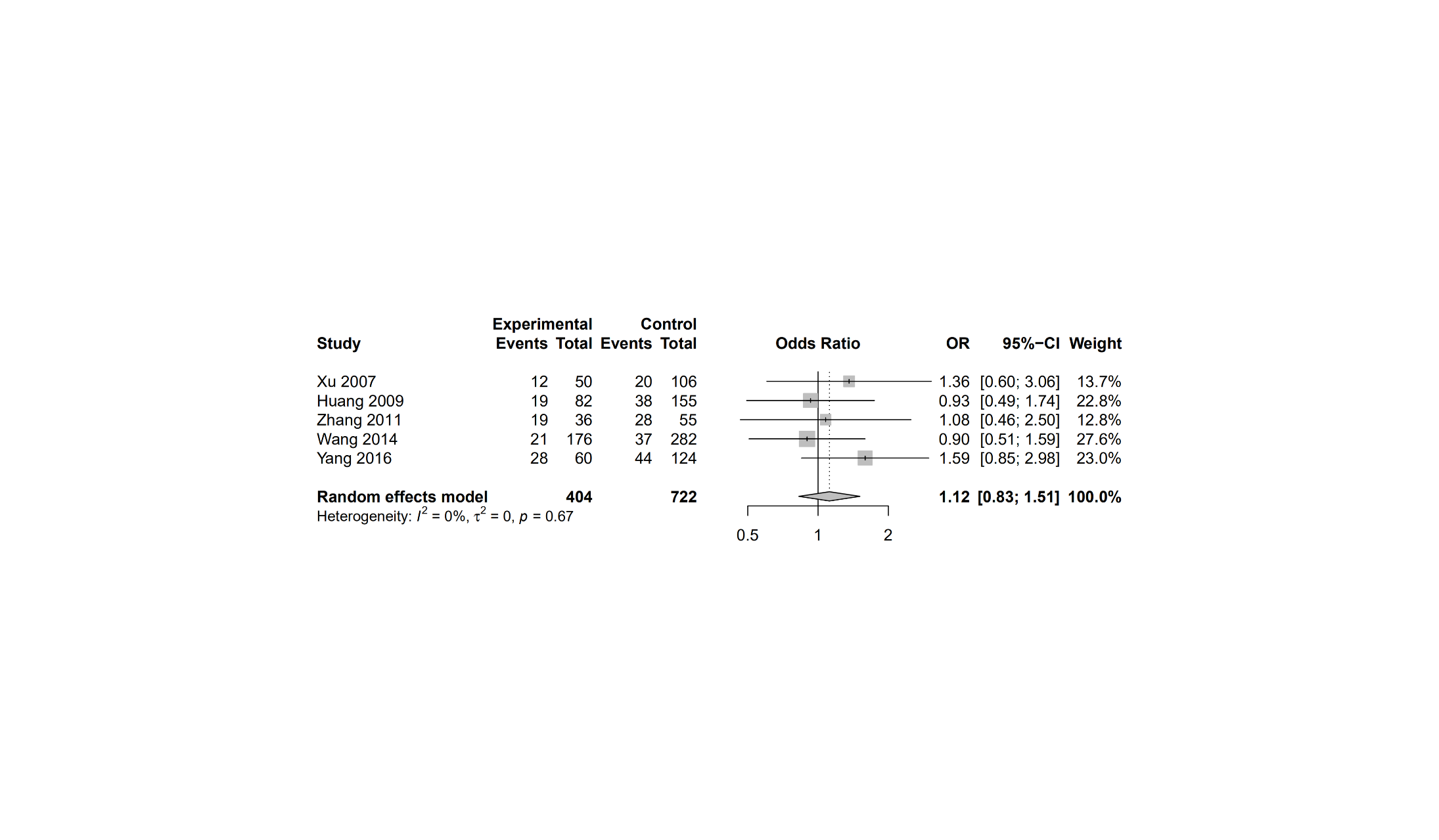


(E) OR (≥10 years vs. <5 years)


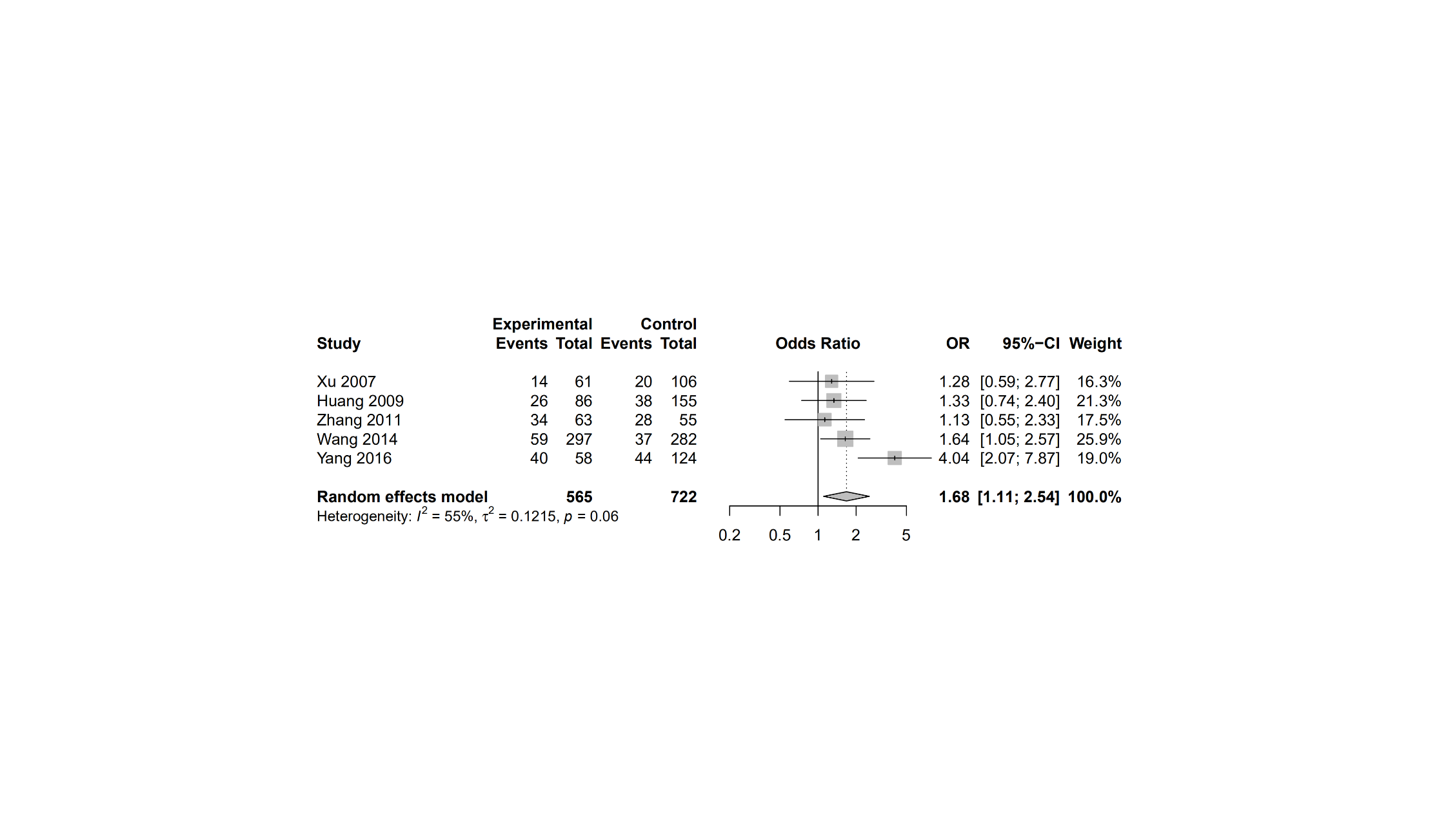


**Figure S7 Forest plot of the prevalence of depression according to the insulin use**

(A) Insulin user


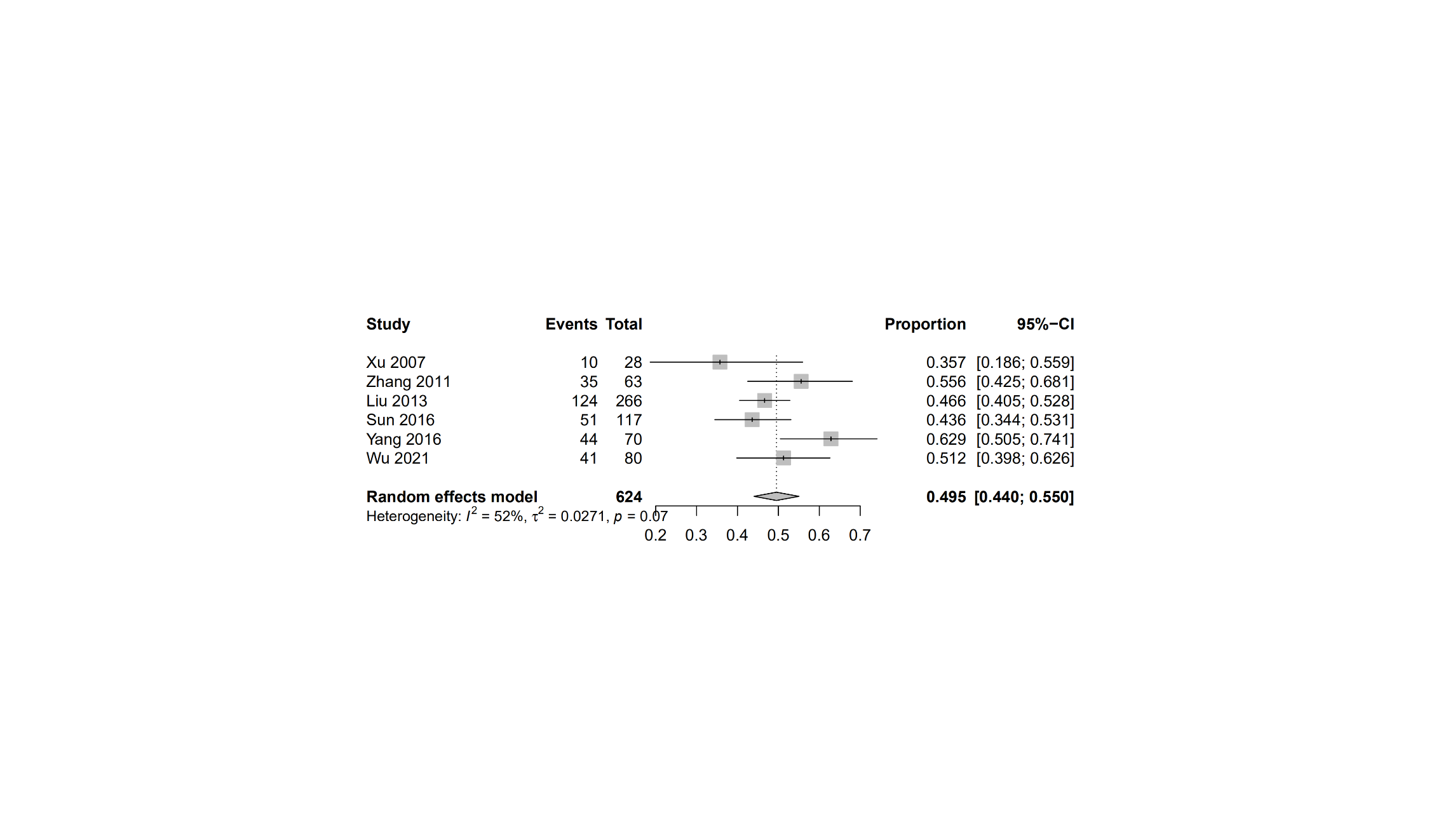


(B) Non-insulin users


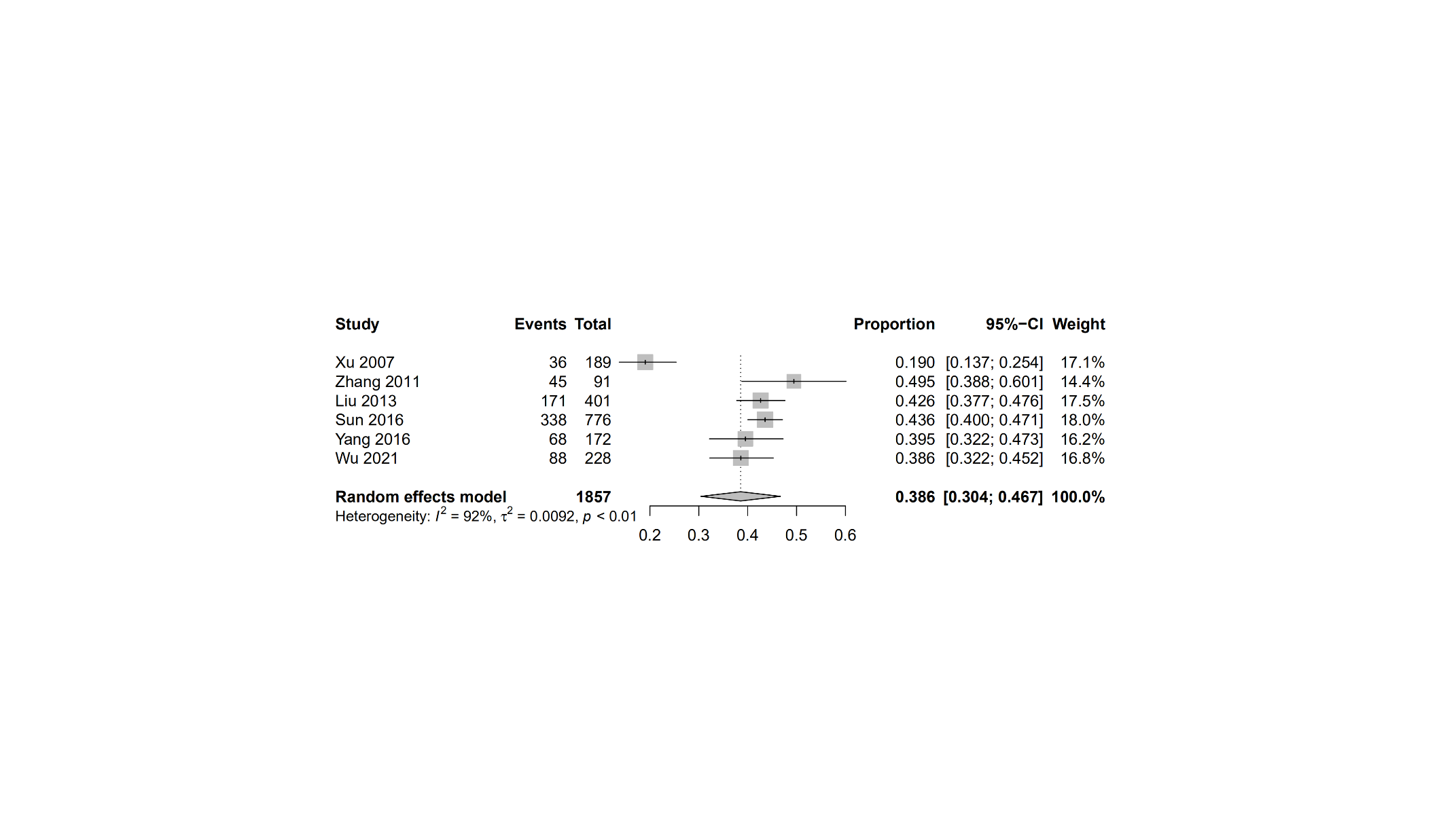


(C) OR (Insulin user vs. Non-insulin users)


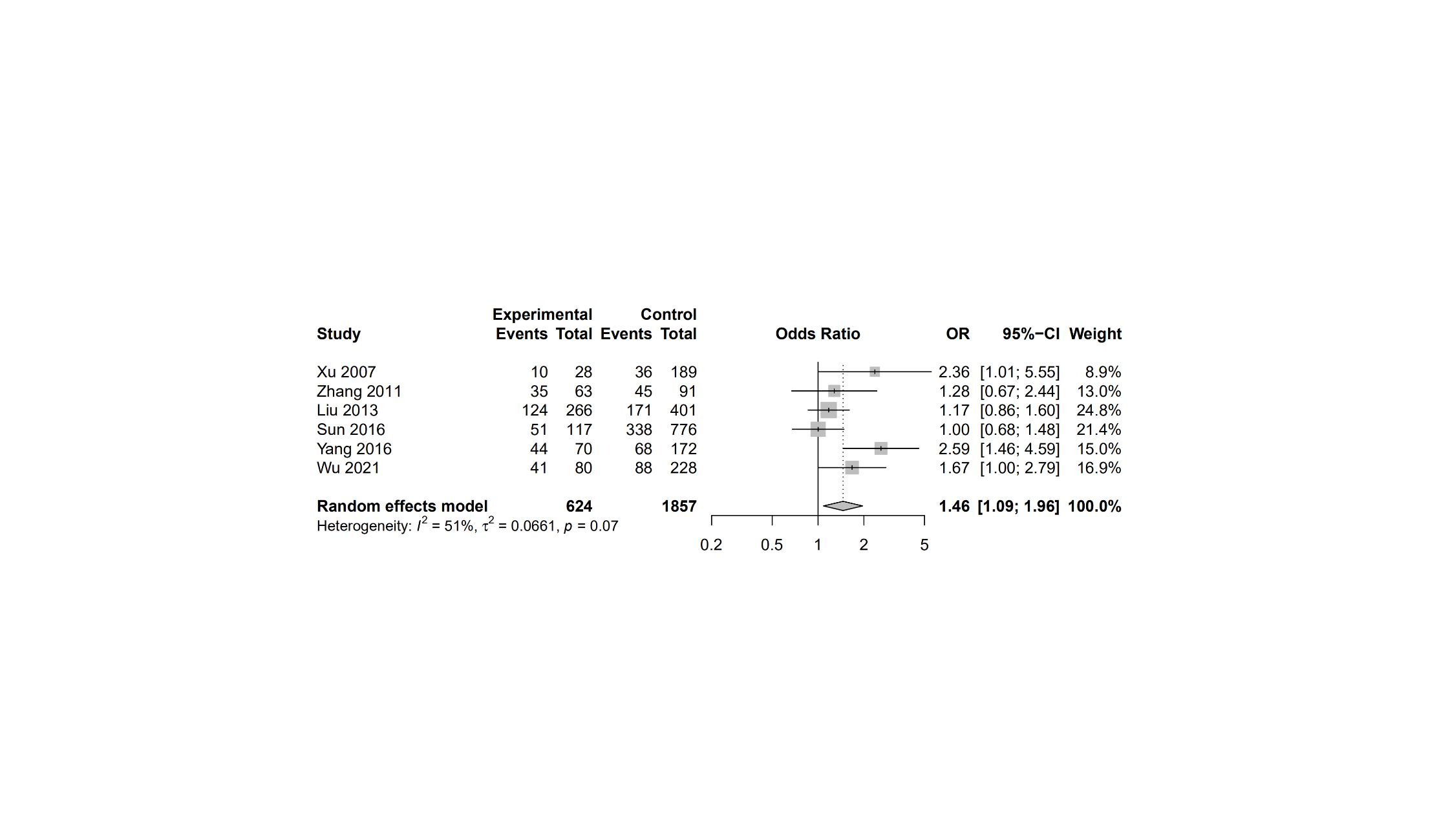


**Figure S8 Forest plot of the prevalence of depression according to the complications**

(A) T2DM with complications


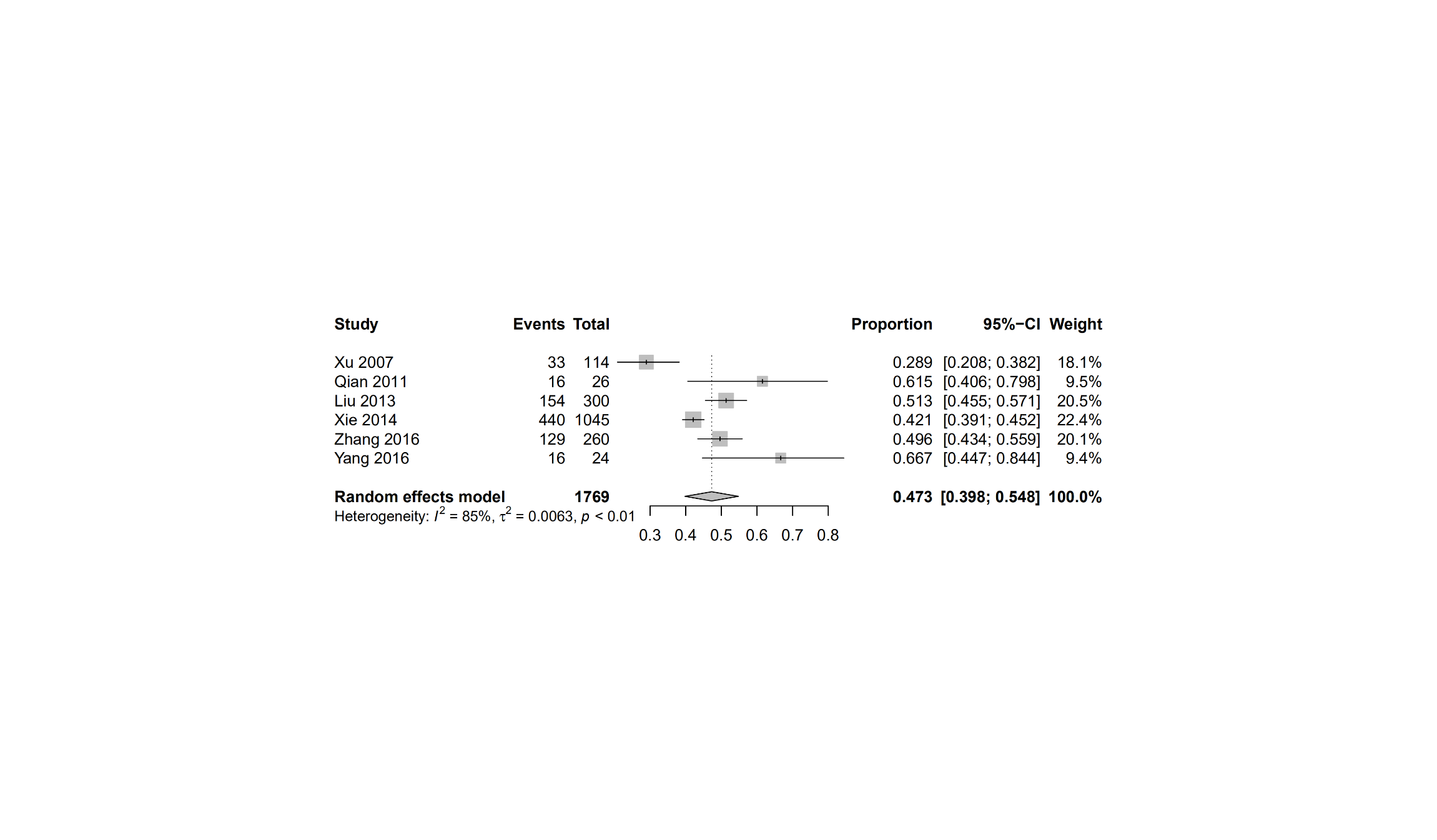


(B) T2DM without complications


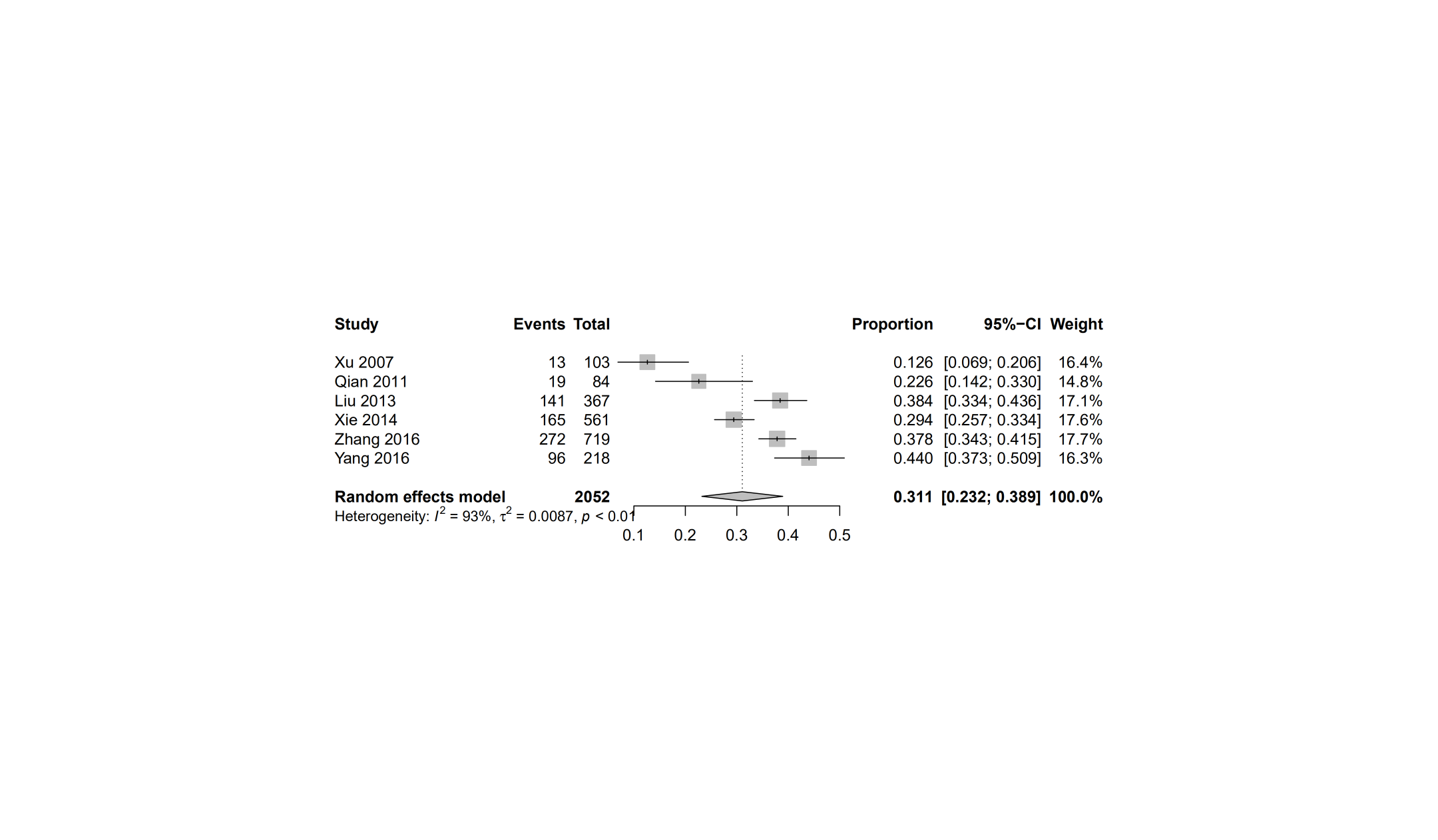


(C) OR (T2DM with complications vs. T2DM without complications)


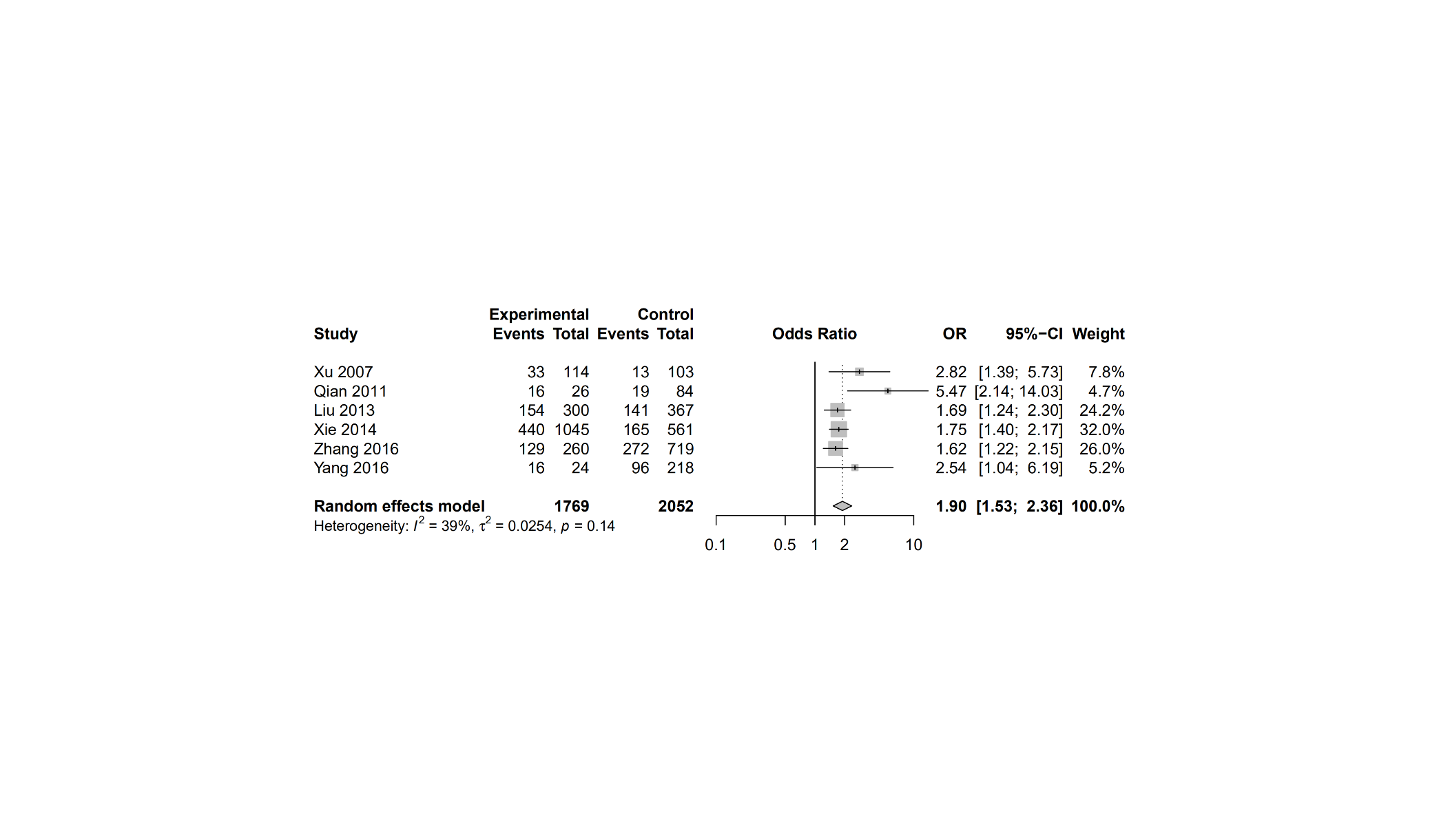


**Figure S9 Forest plot of the prevalence of depression according to current smoking status**

(A) Current smoker


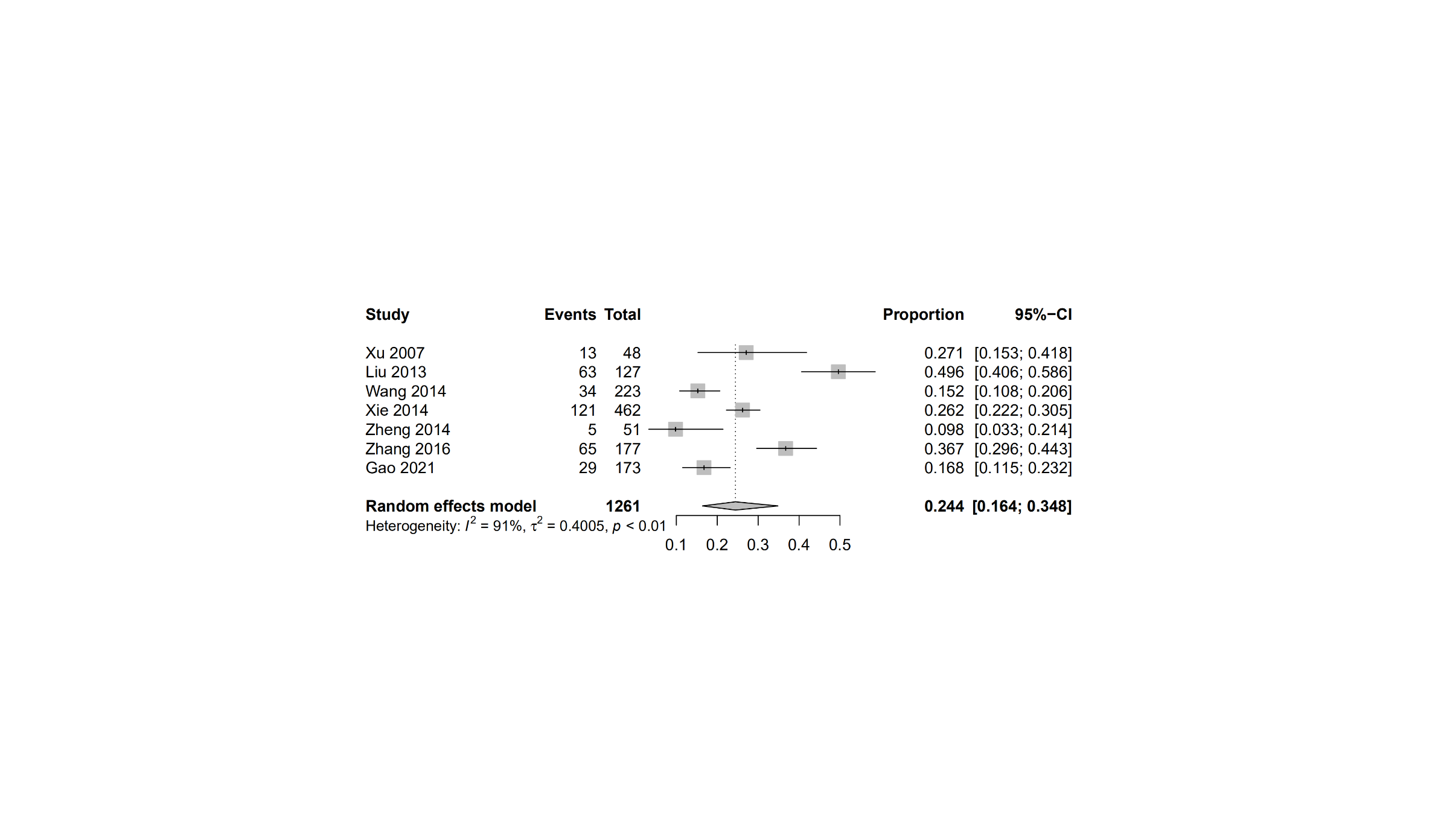


(B) Non-smoker


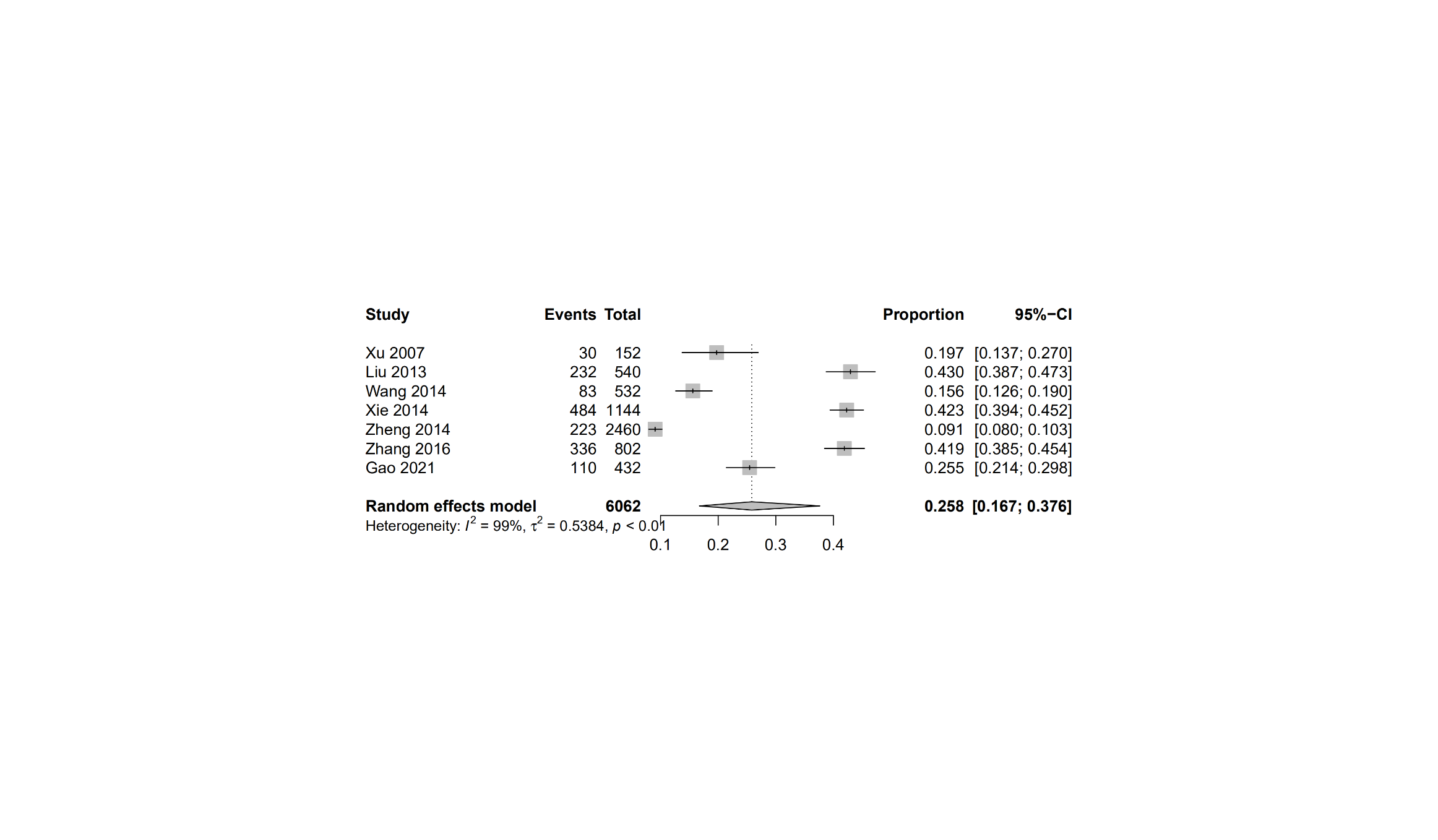


(C) Current smoker vs. non-smoker


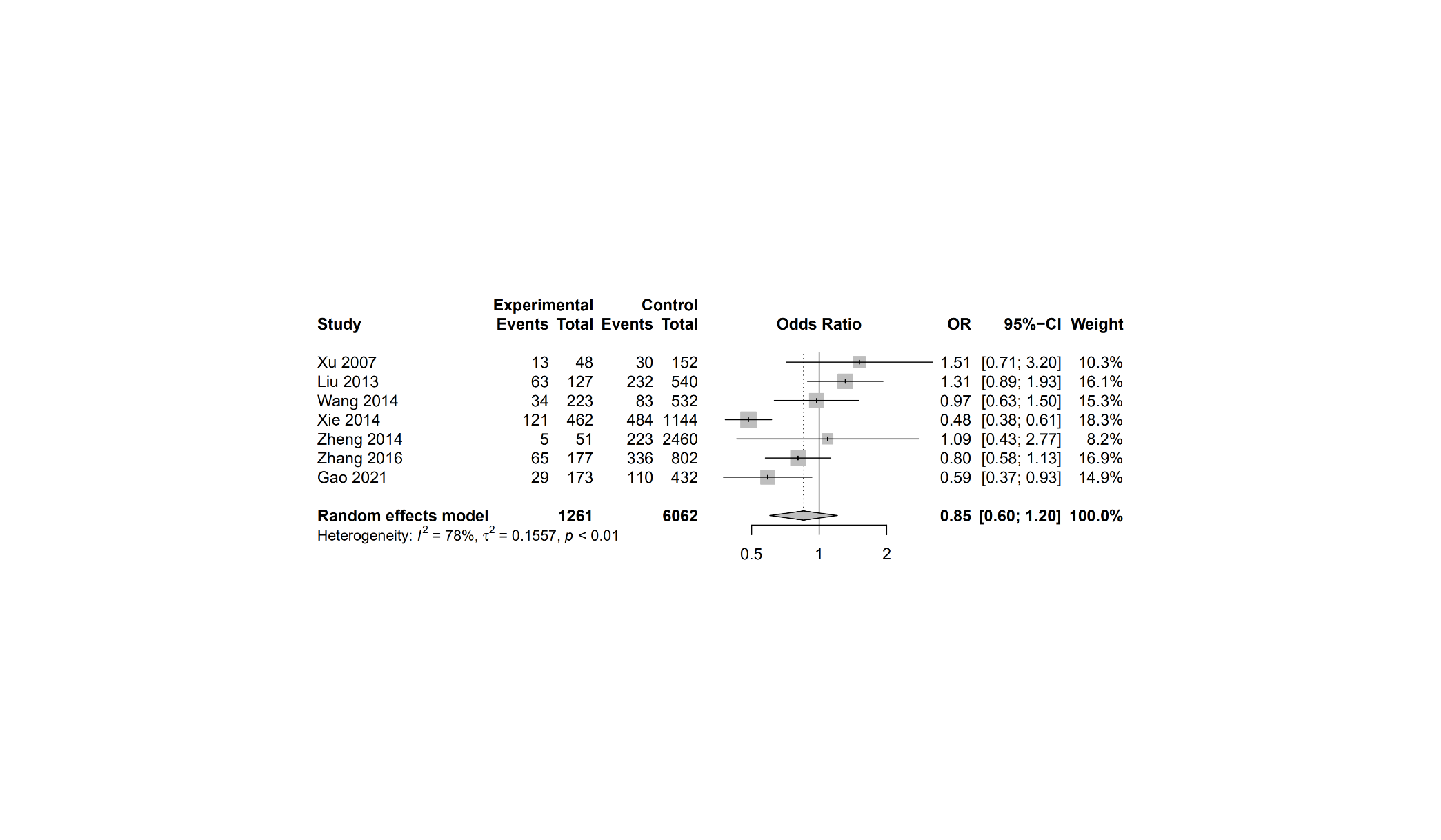


**Figure S10 Forest plot of the prevalence of depression according to current alcohol use**

(A) Current alcohol user


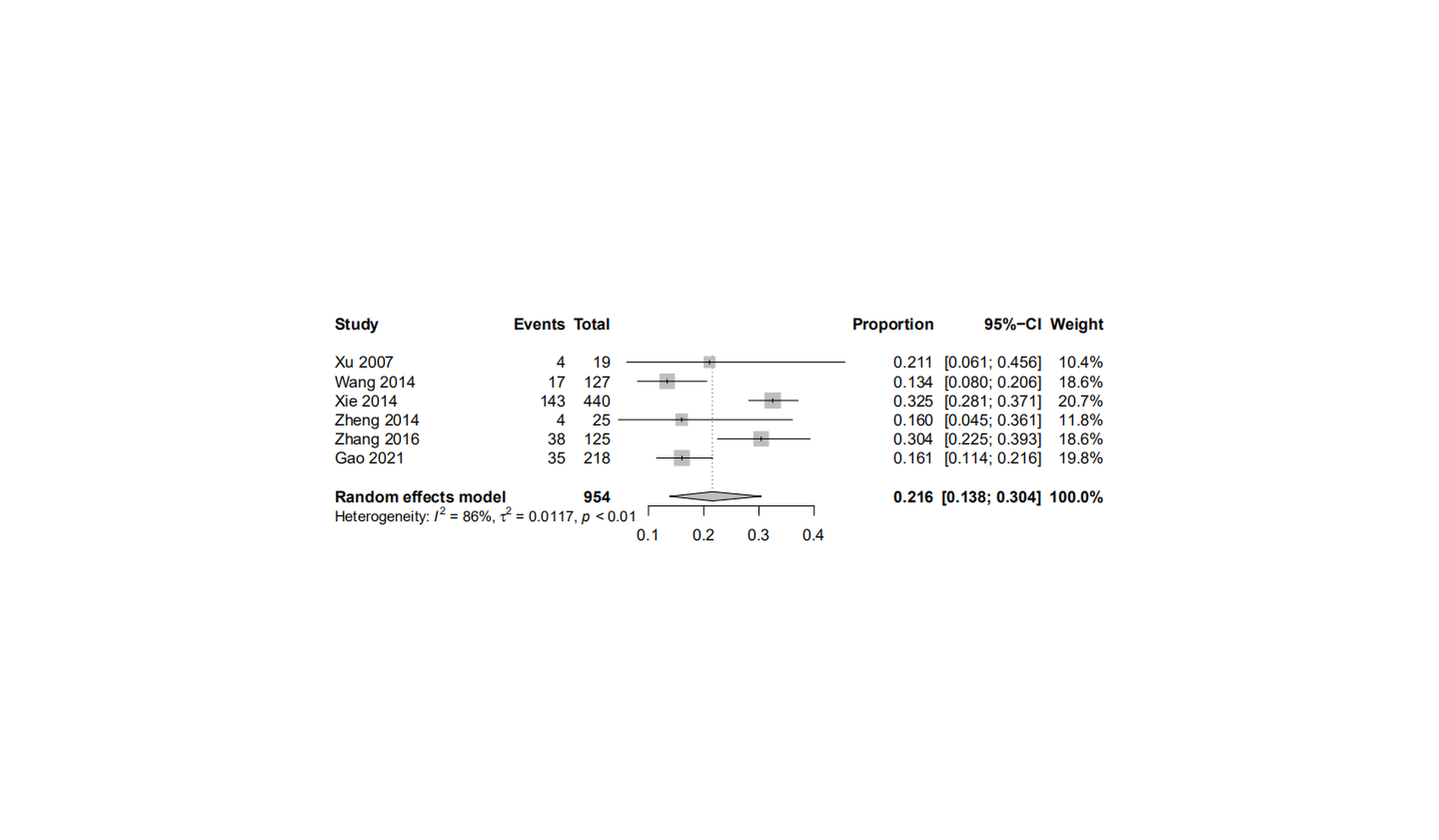


(B) Non-drinker


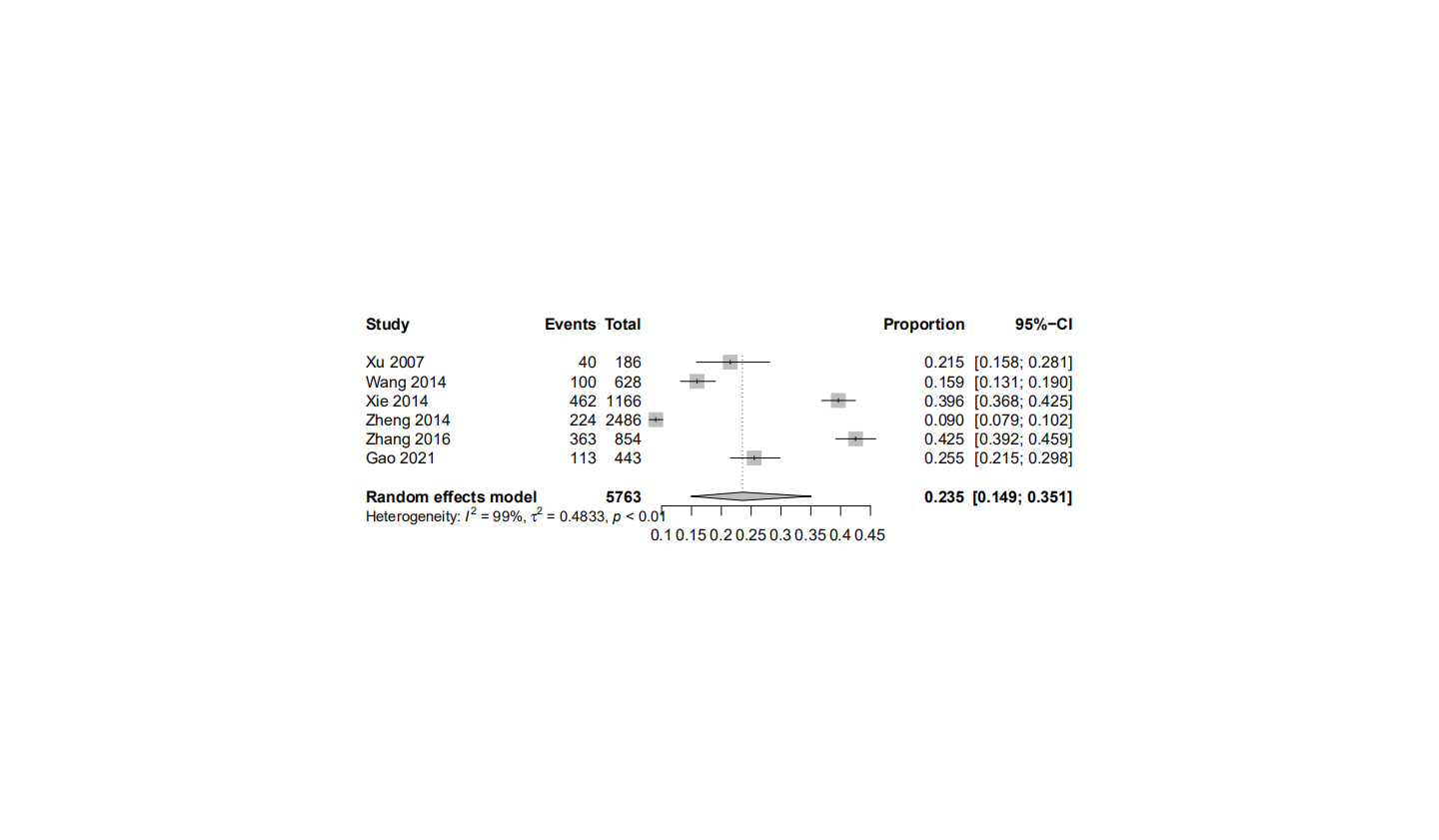


(C) OR (Current alcohol user vs. non-drinker)


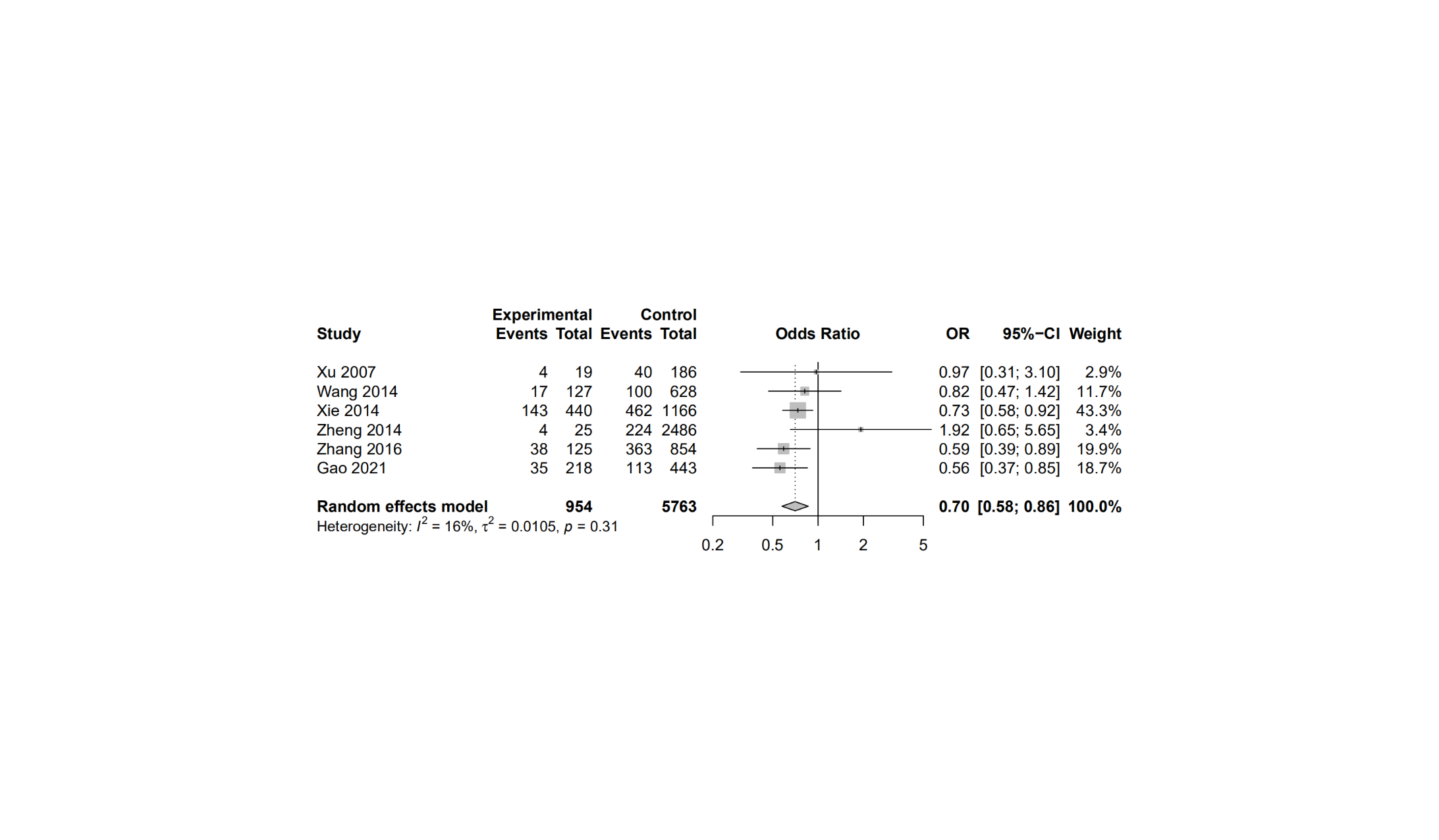


**Figure S11 Forest plot of the prevalence of depression according to marital status**

(A) Abnormal marital status


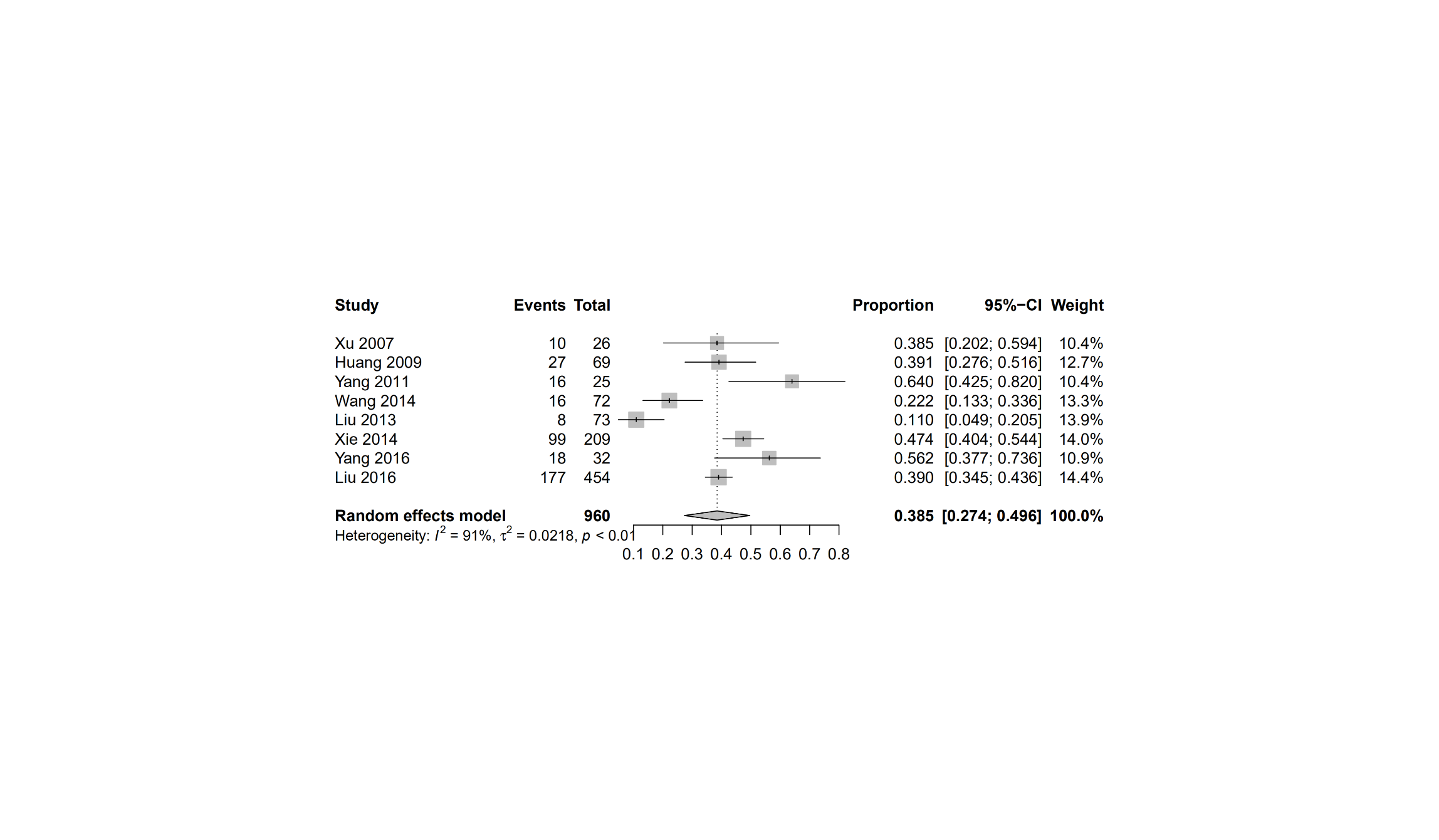


(B) Normal marital status


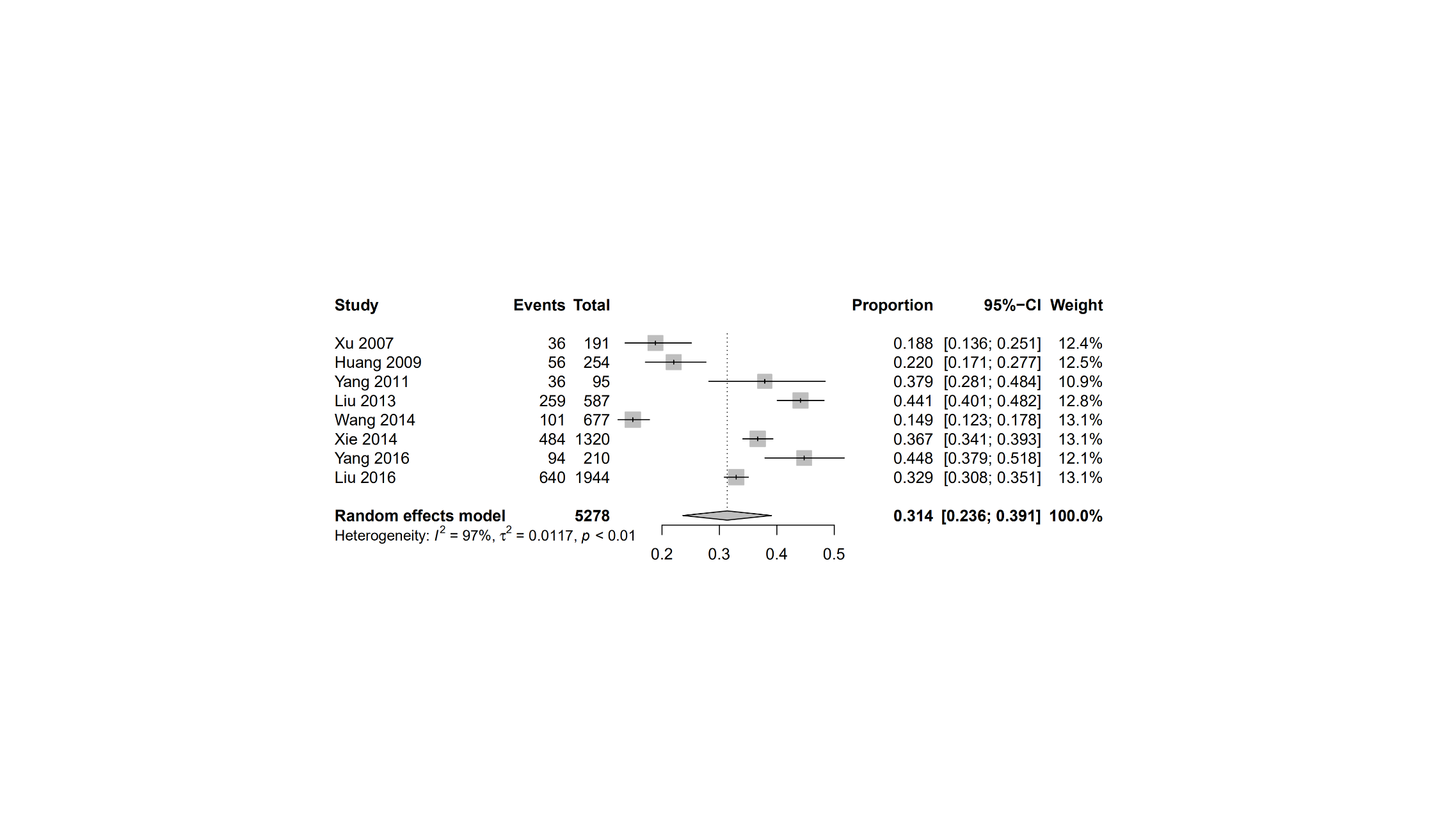


(C) OR (Abnormal marital status vs. normal marital status)


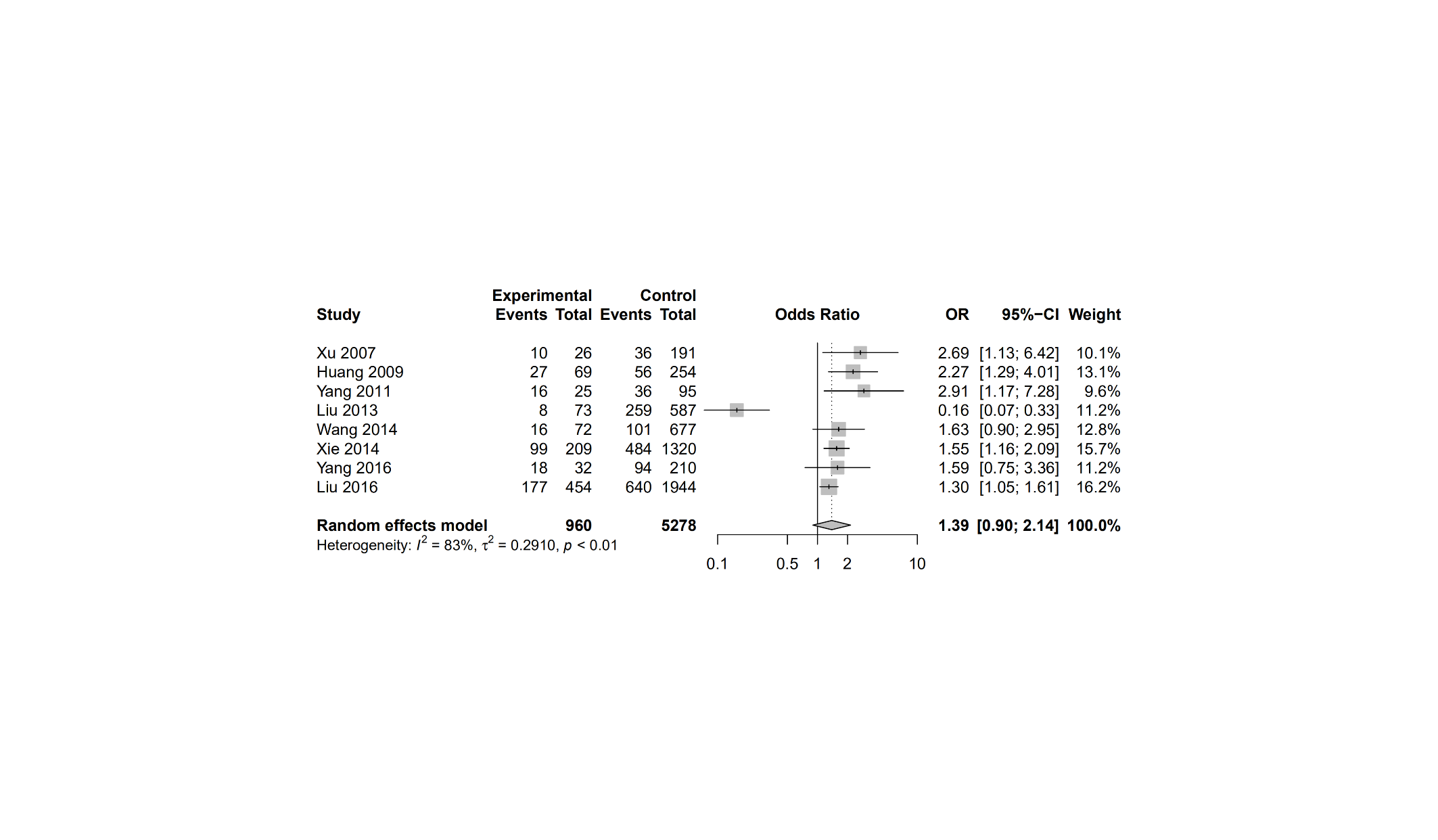


**Figure S12 Forest plot of the prevalence of depression according to living status**

(A) Living alone


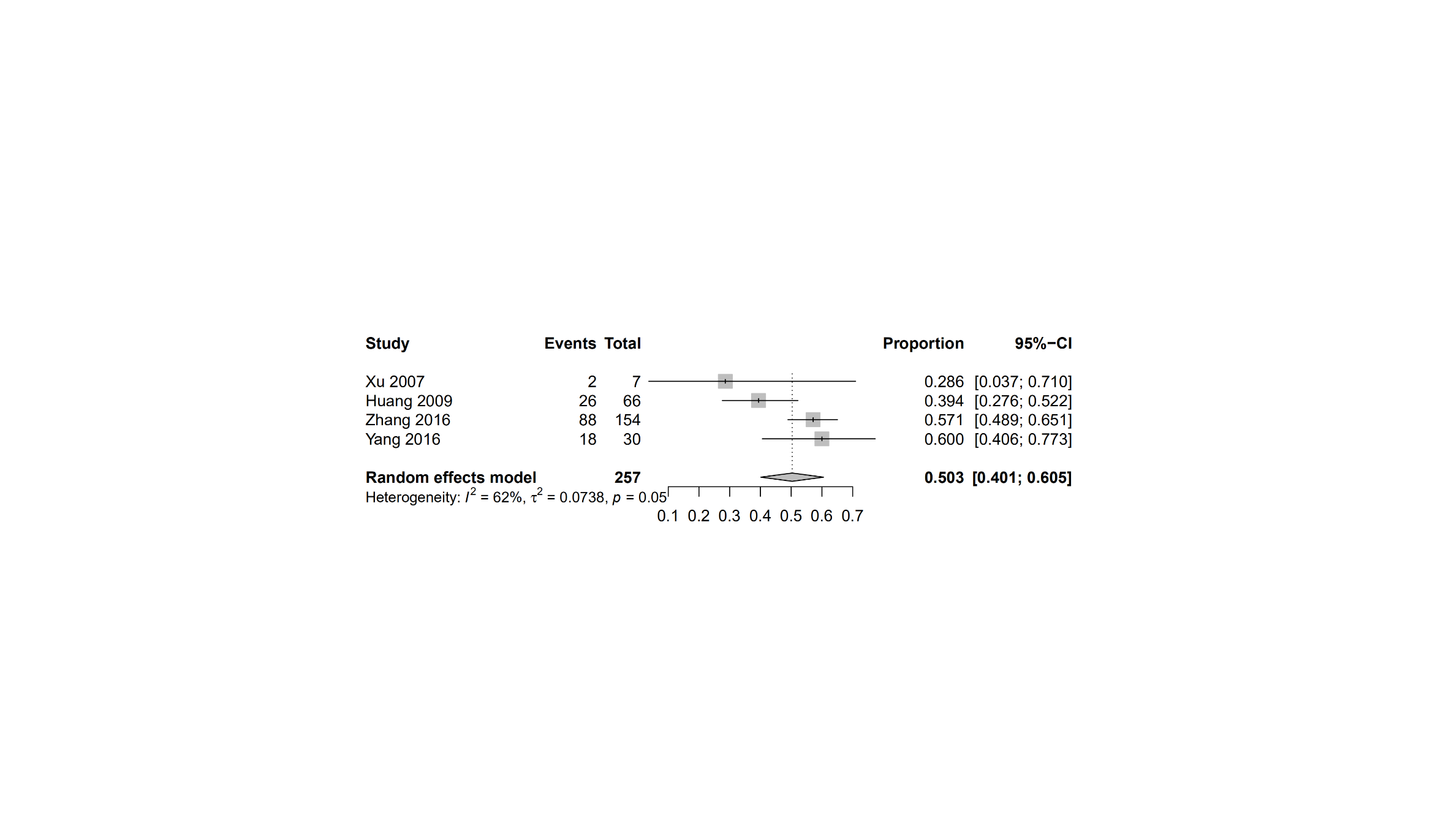


(B) Not living alone


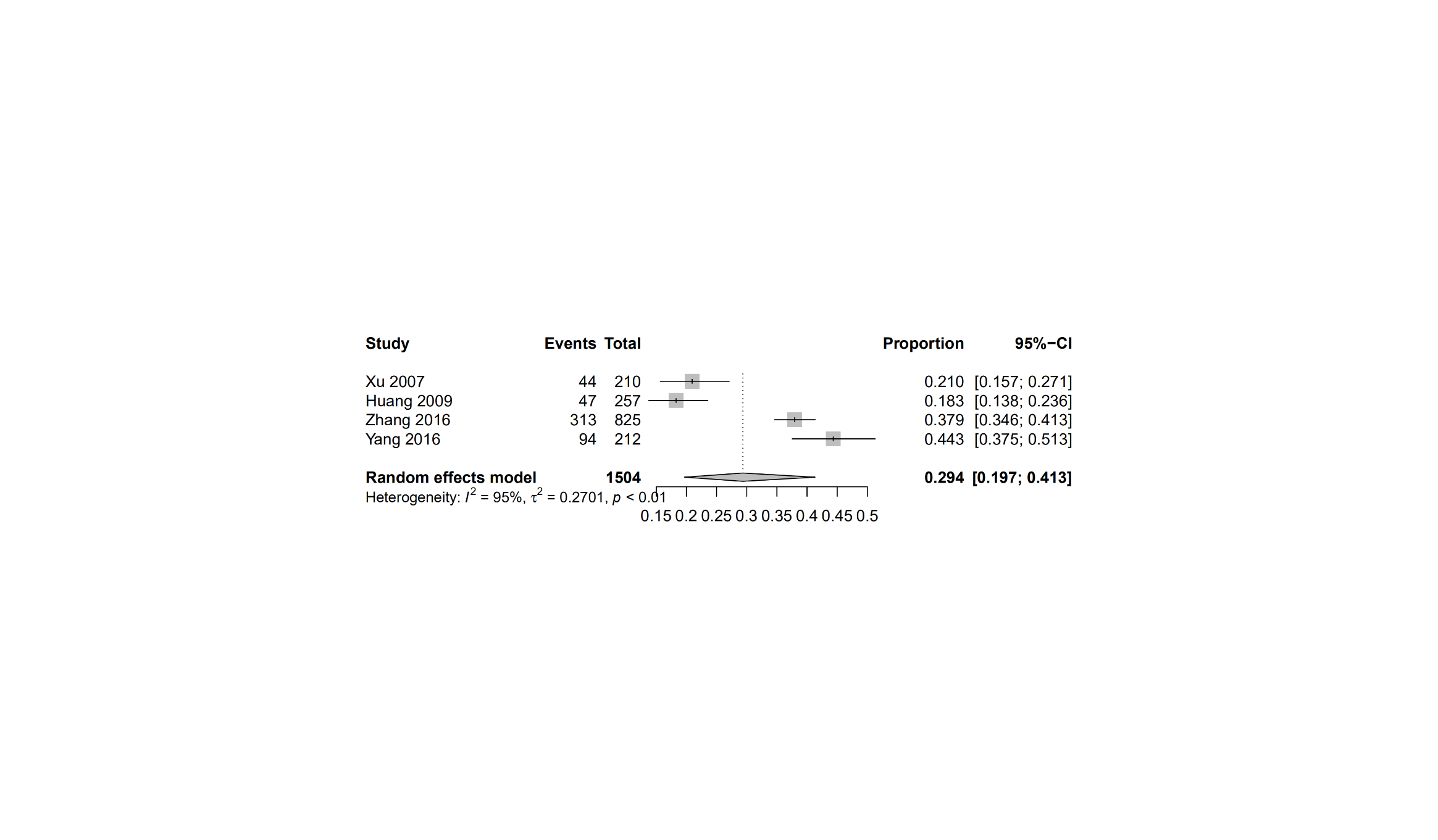


(C) OR (Living alone vs. Not living alone)


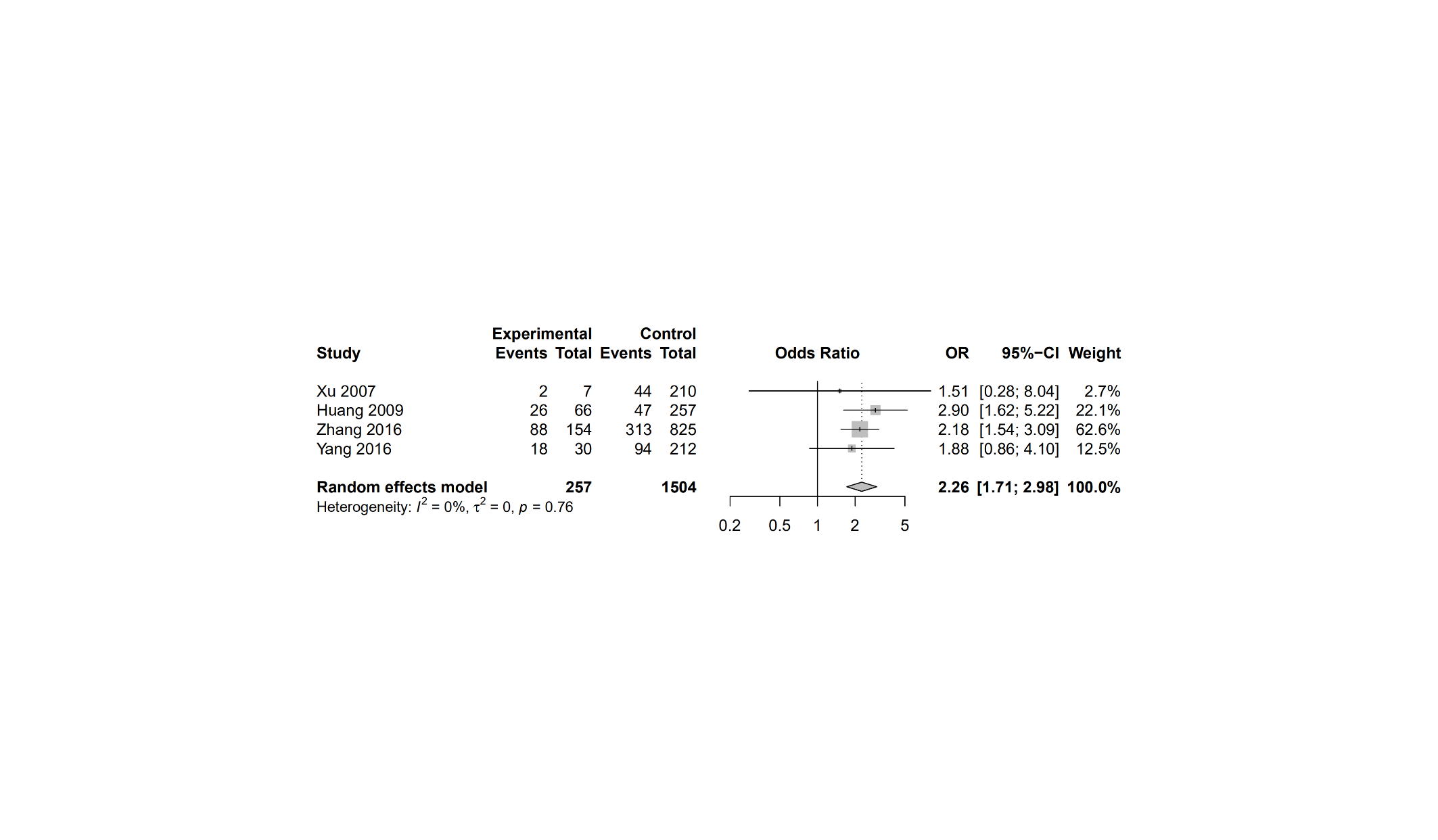


**Figure S13 Forest plot of the prevalence of depression according to different instruments used to detect depression**

(A) SDS ≥ 50


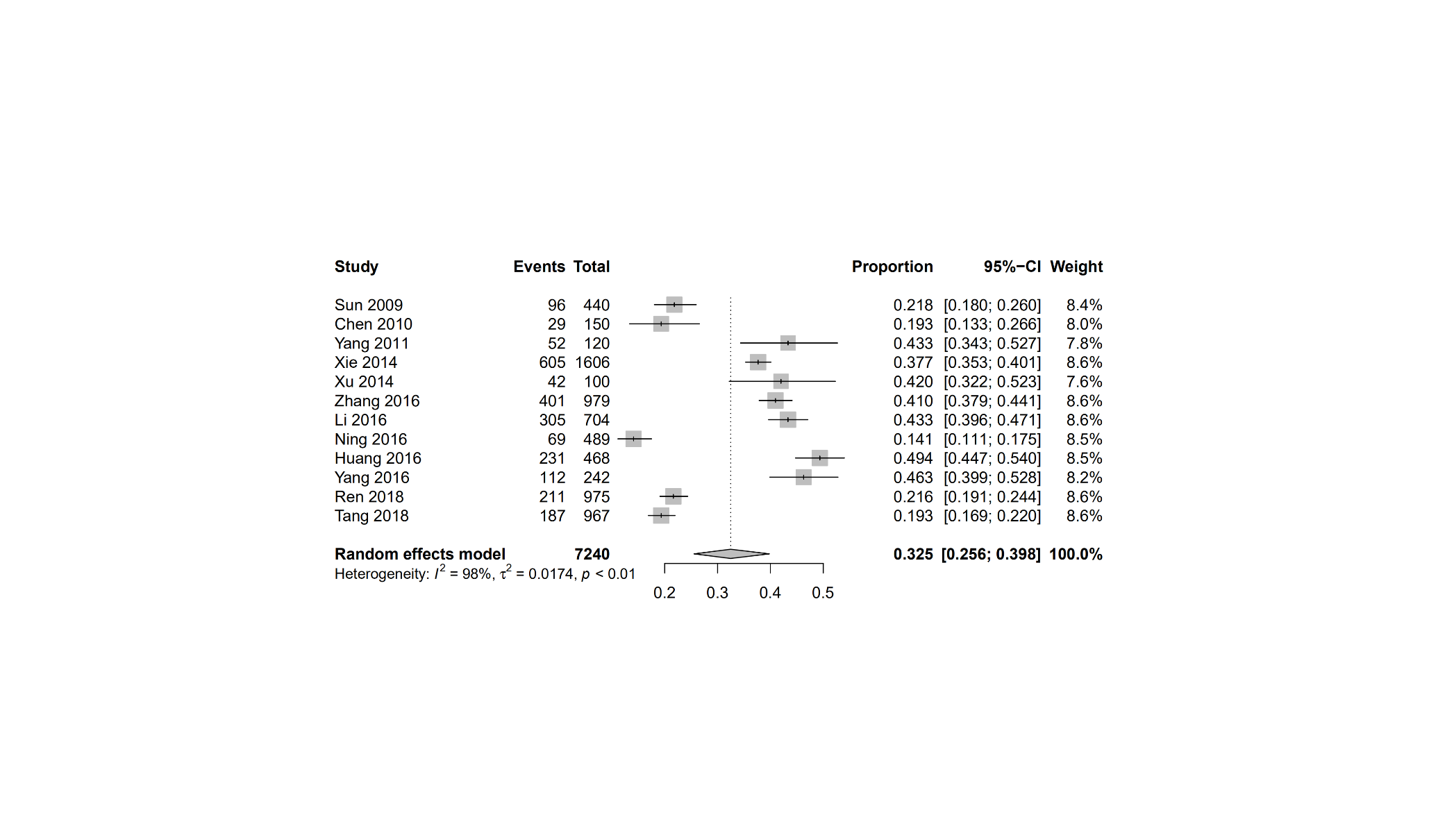


(B) SDS ≥ 53


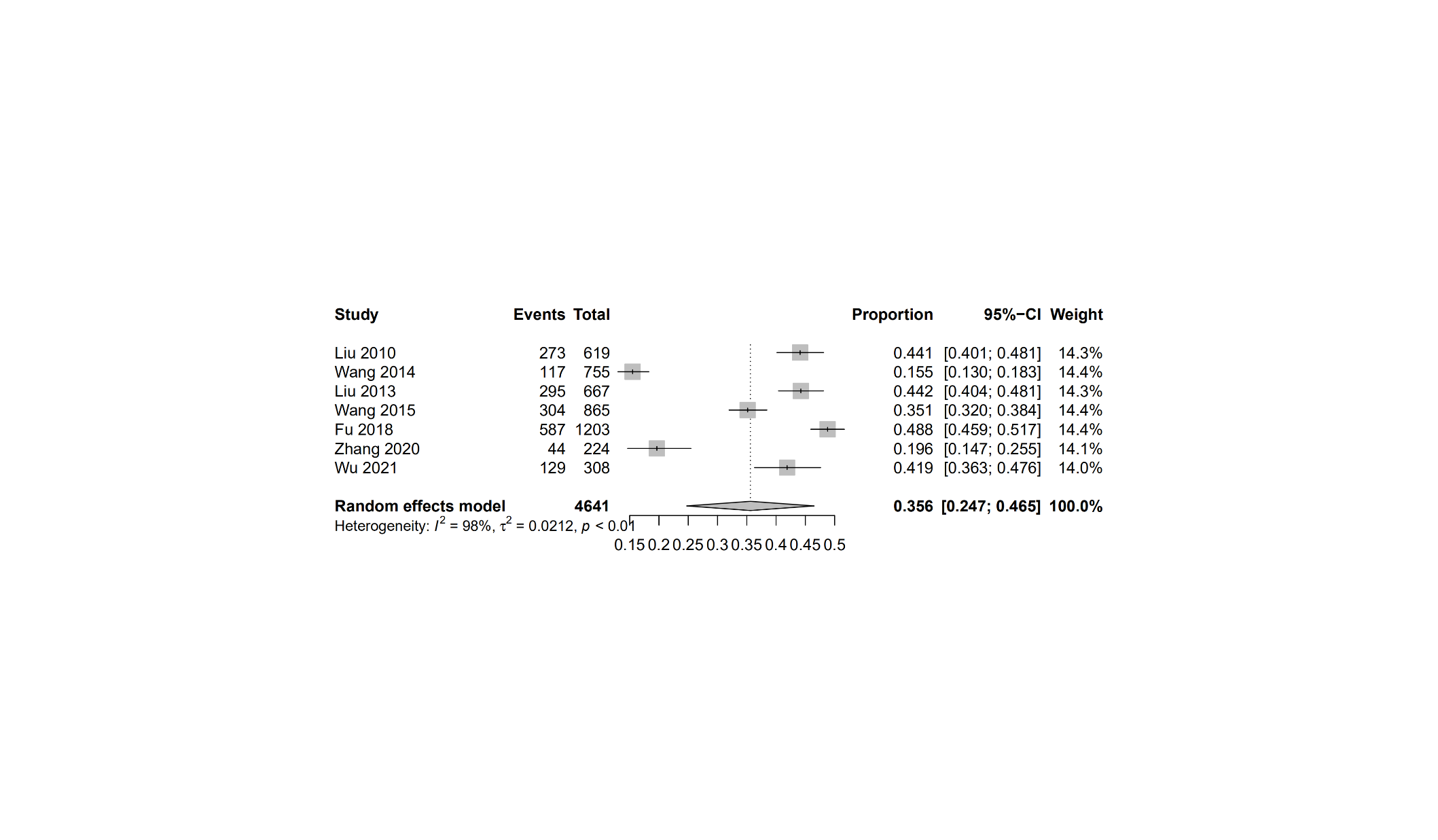


(C) PHQ-9 ≥5


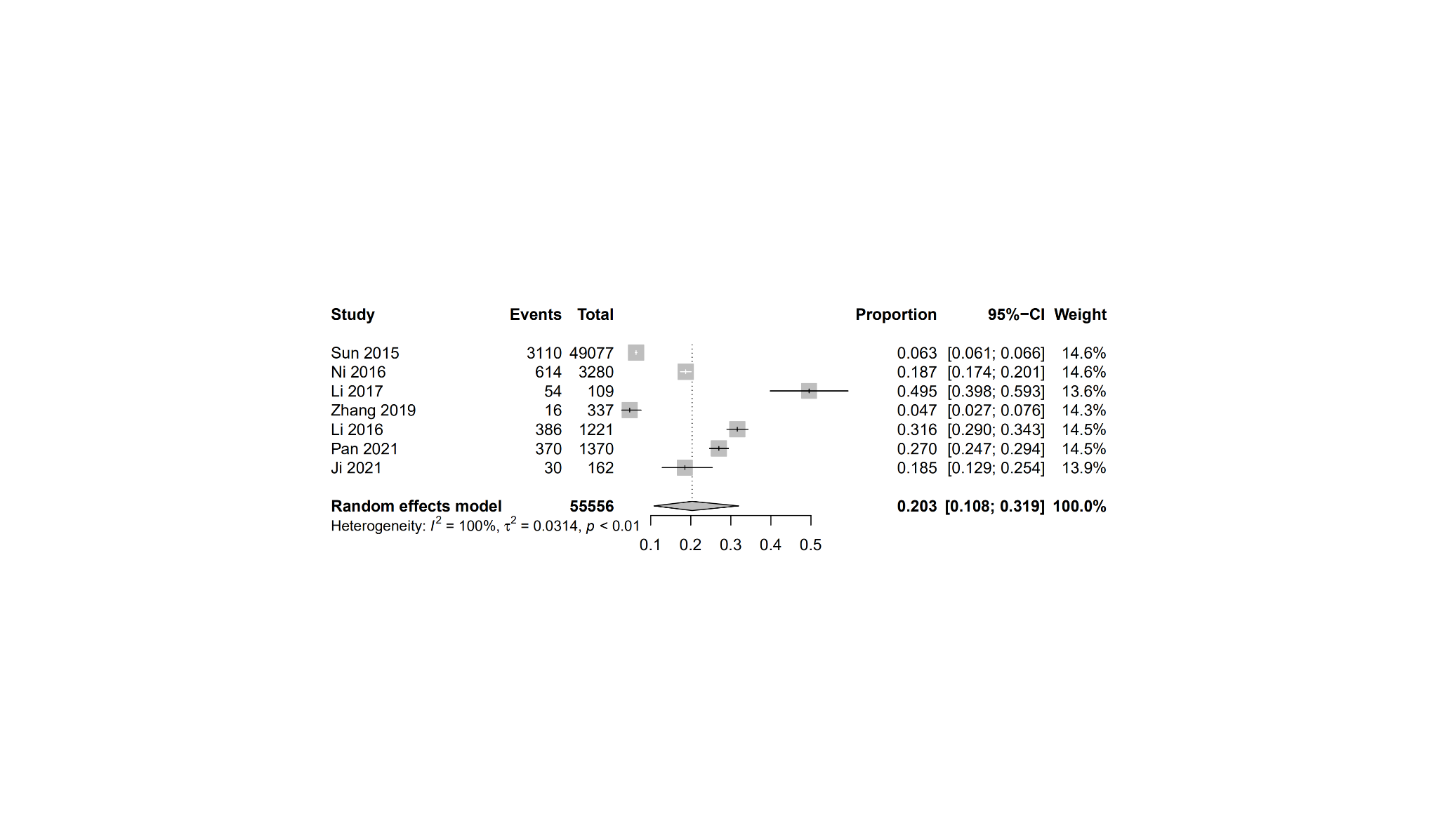


(D) PHQ-9 ≥10


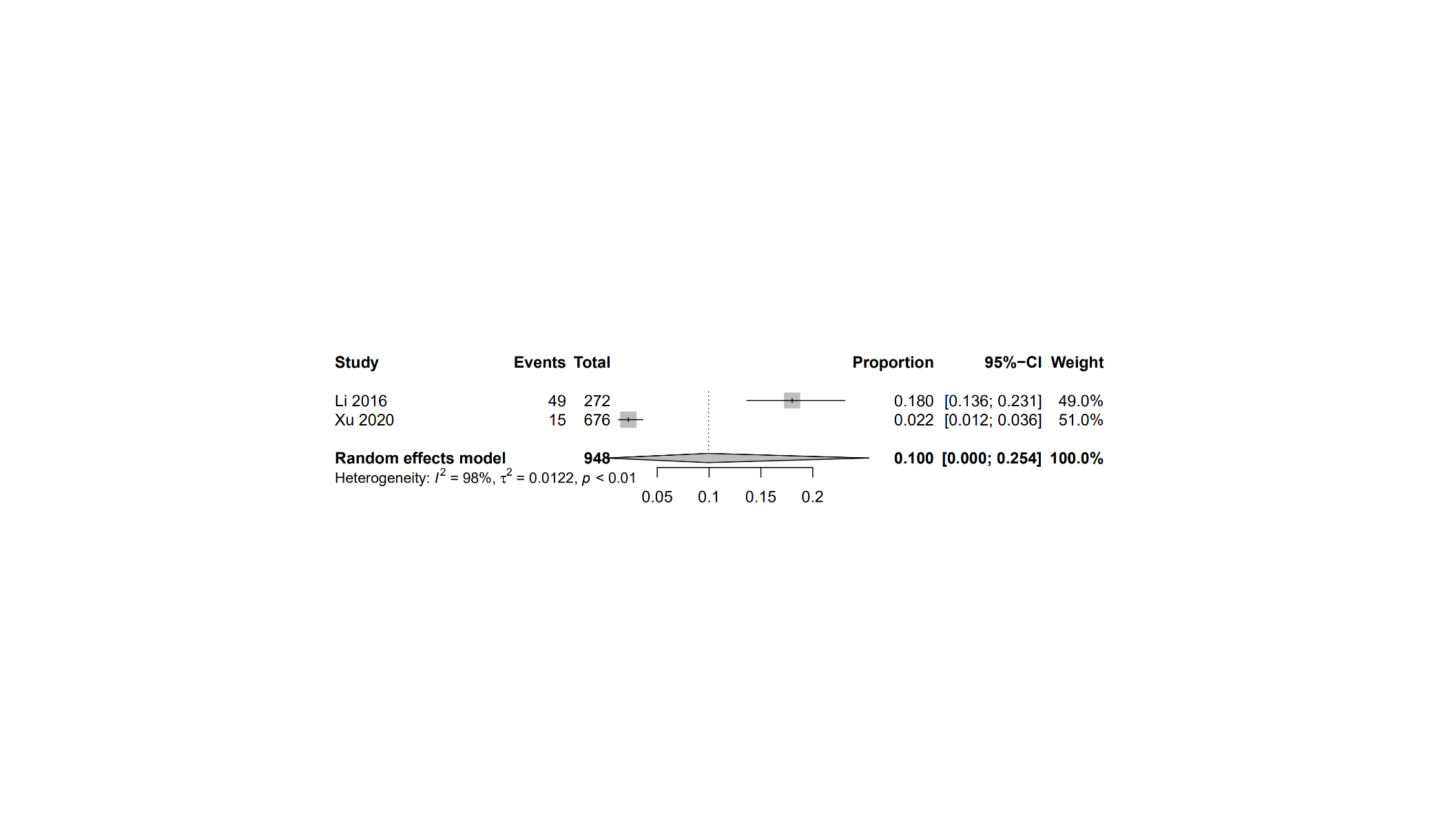


(E) CESD-20 ≥16


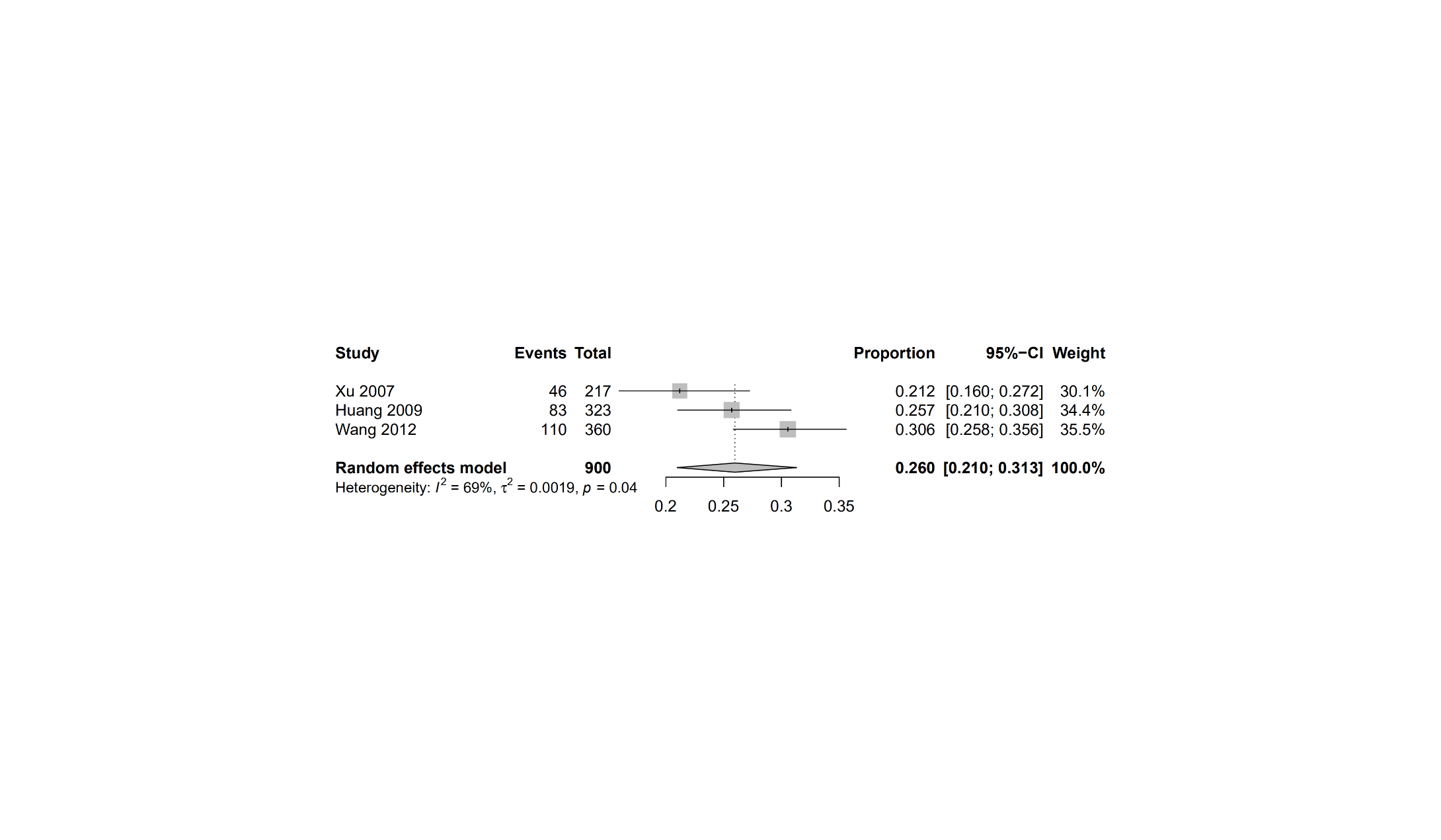


(E) GDS-15 ≥6


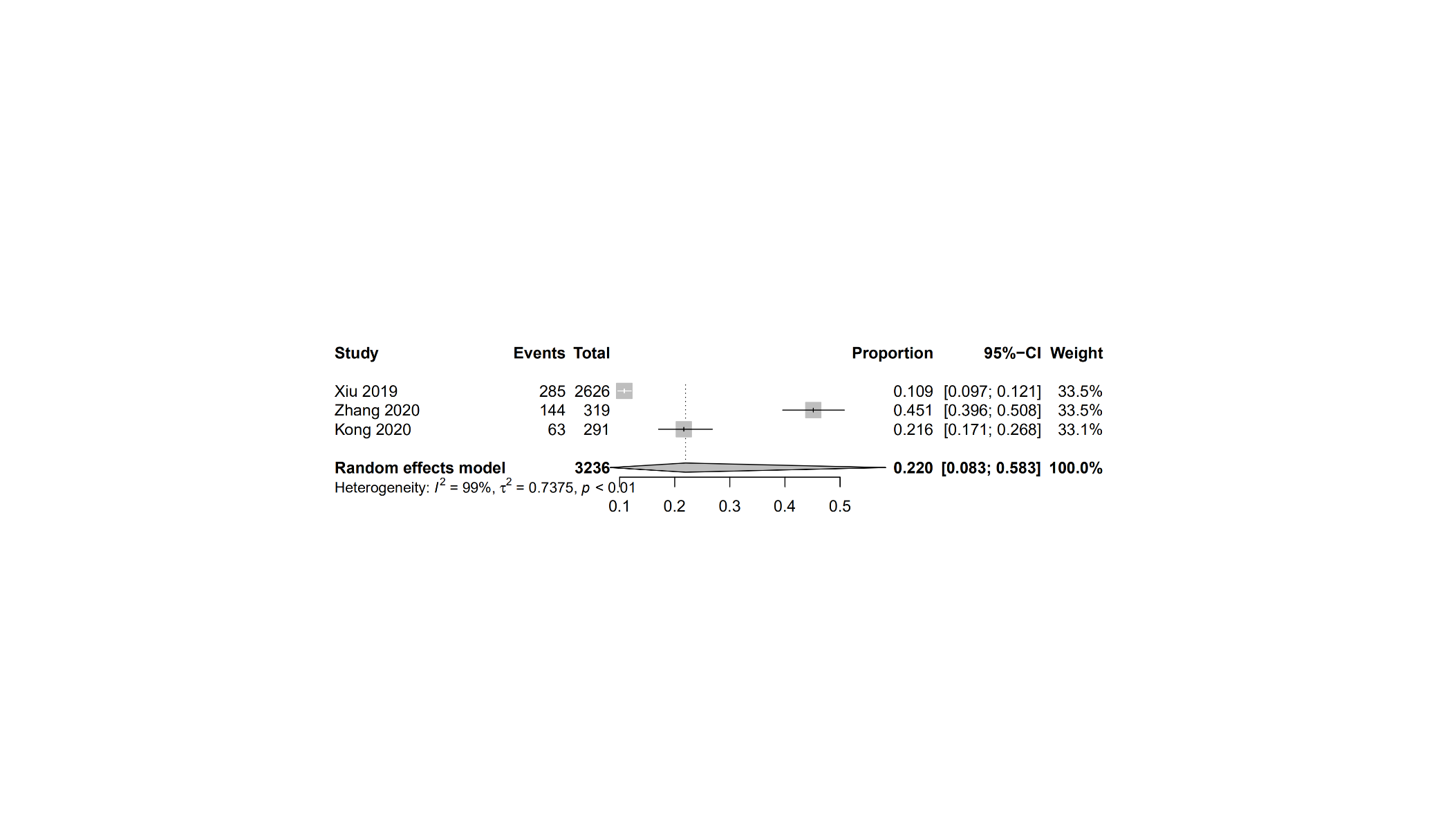


(F) GDS-15 ≥8


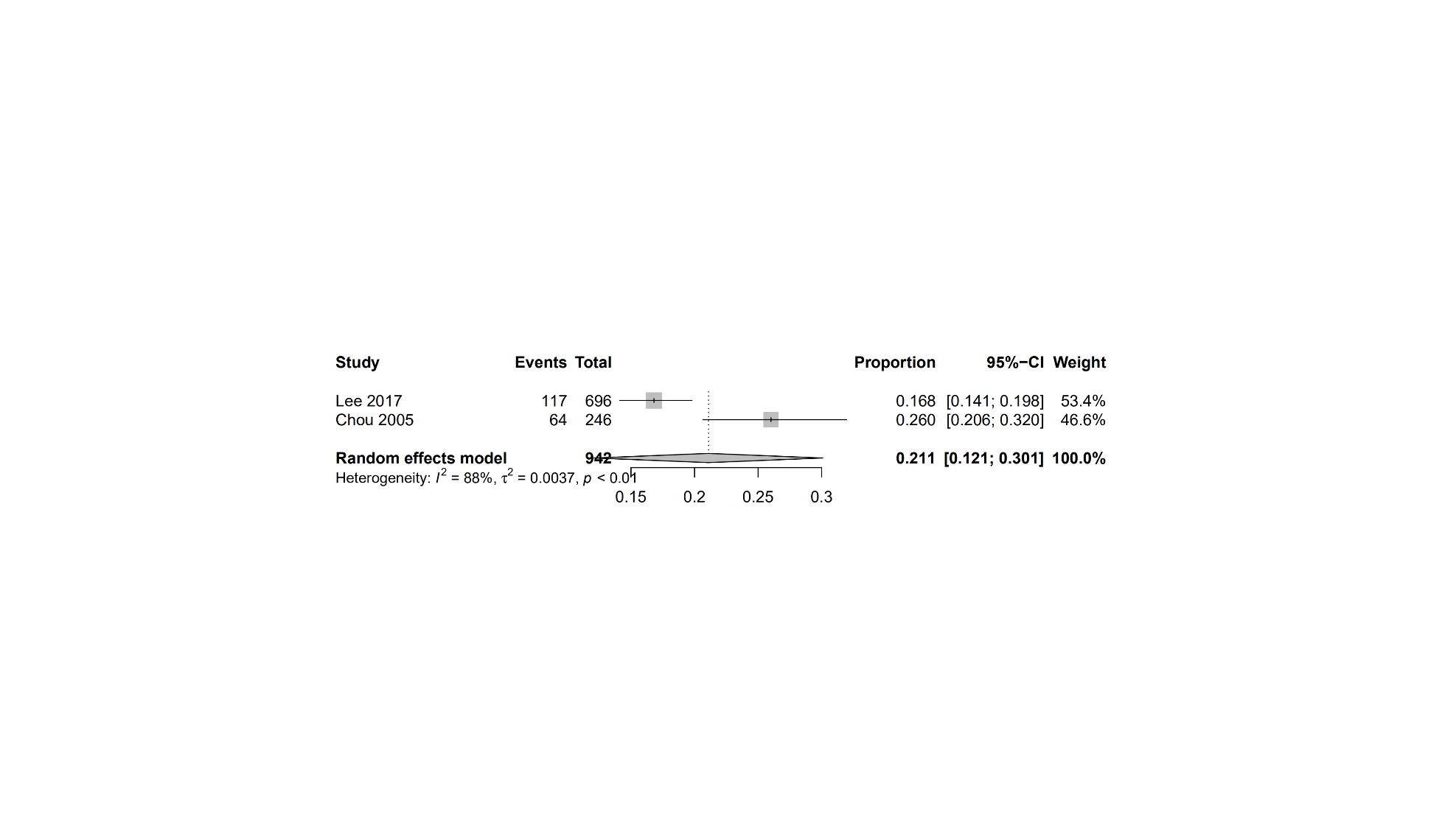


**Figure S14 Forest plot of the prevalence of depression according to risk of bias**

1. Low risk of bias


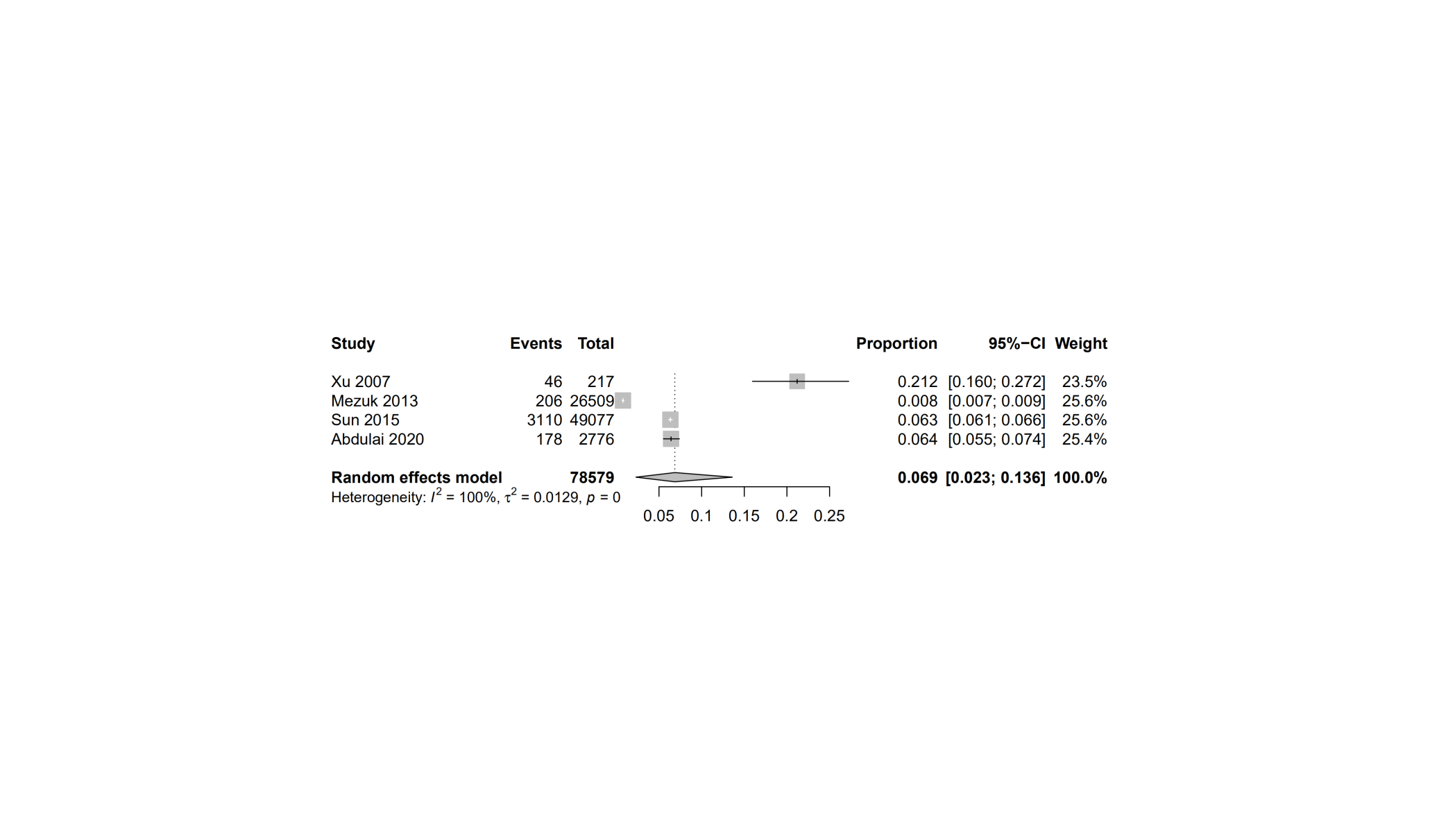


1. Moderate risk of bias


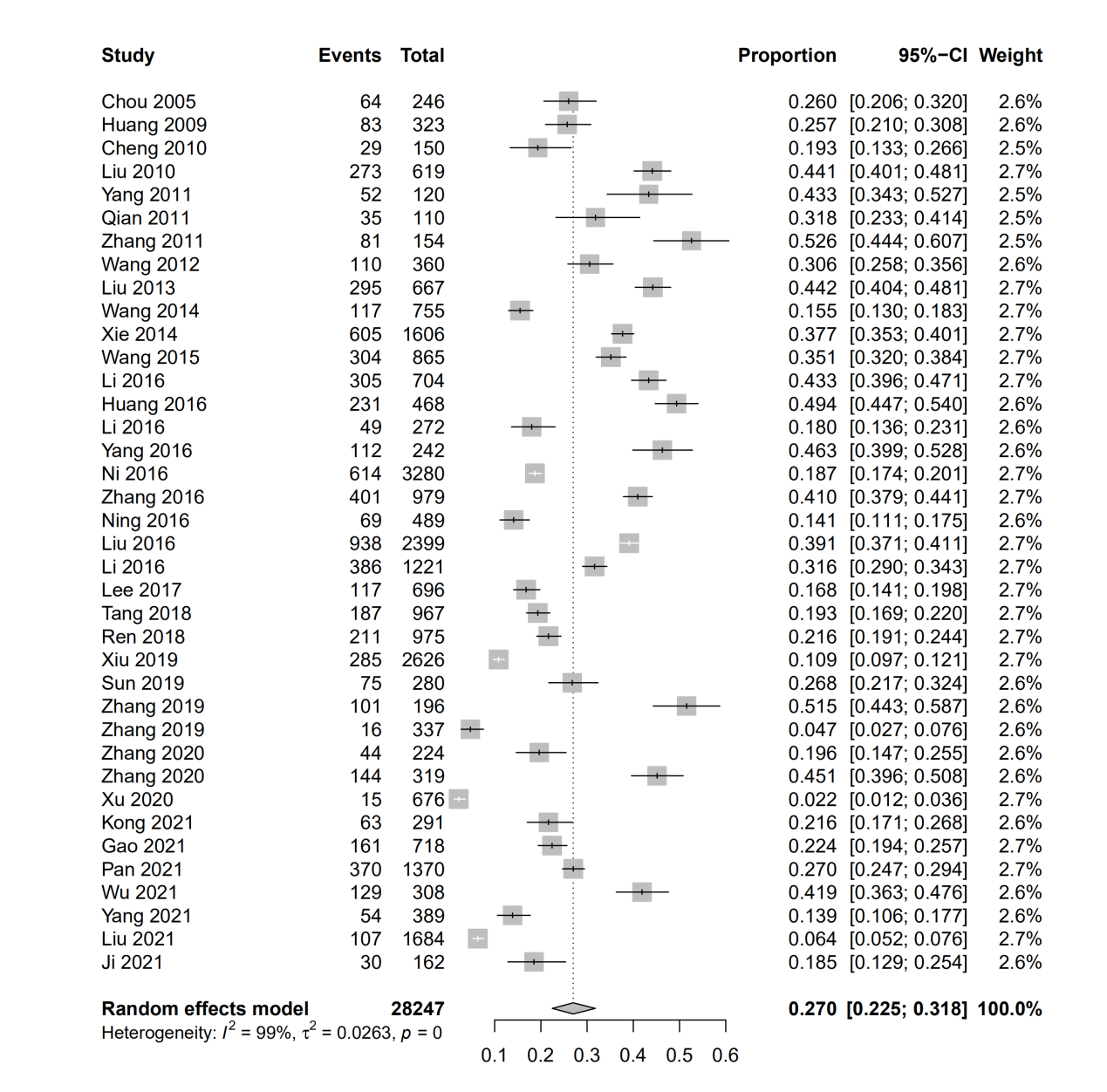


1. High risk of bias


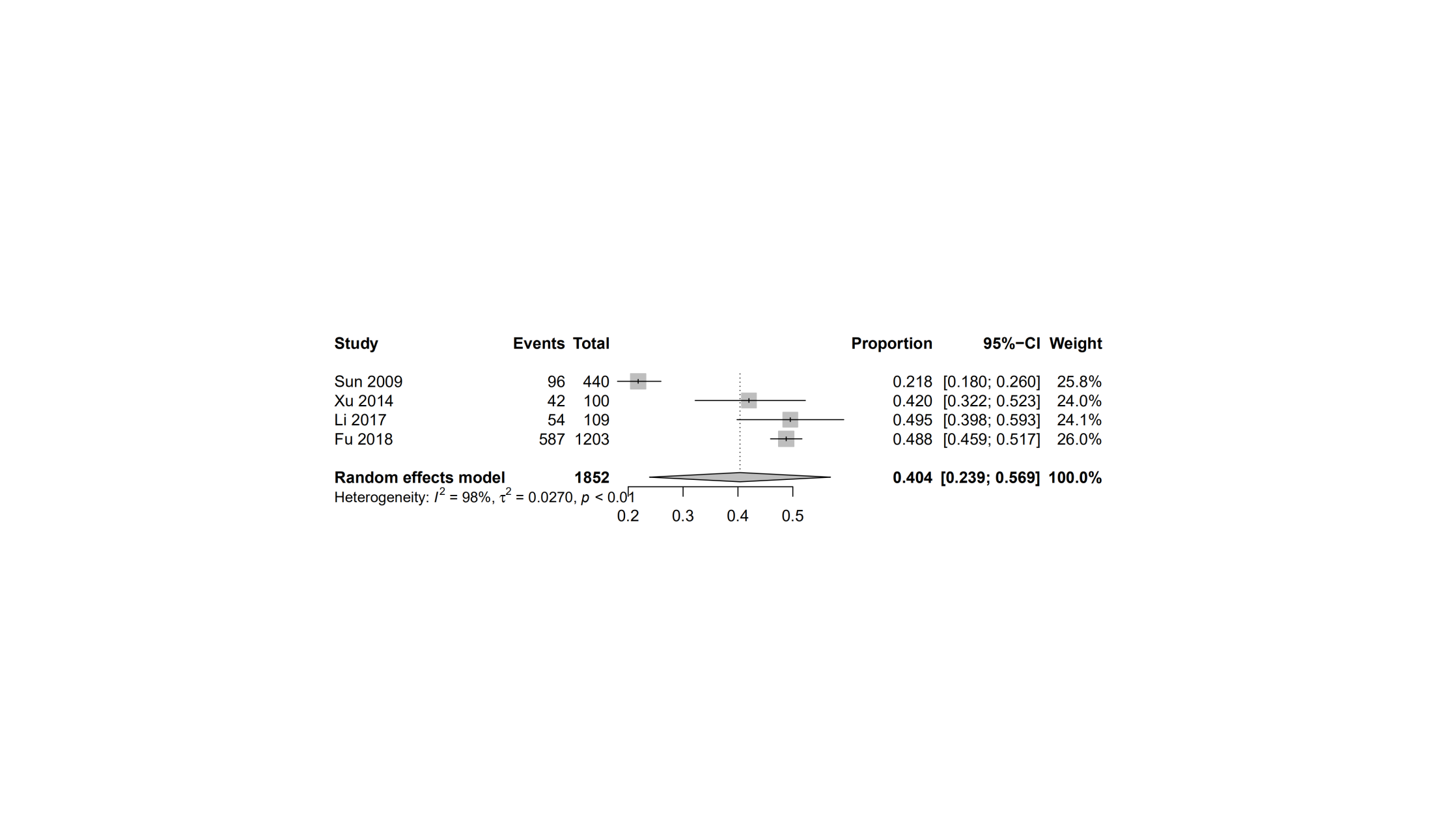

Supplement: Supplementary File 5 — Forest plot and funnel plot of subgroup analysis. [file Data_Sheet_5.docx]
